# Extracellular vesicles in the immune regulation of hematological malignancies: mechanisms, prognostic significance, and therapeutic opportunities

**DOI:** 10.3389/fcell.2026.1941887

**Published:** 2026-09-29

**Authors:** Tuya Wang, Jing Fu, Ye Huang, Jing Xu, Chun Fu

**Affiliations:** Department of Medicine, Hetao College, Bayannur, China

**Keywords:** extracellular vesicles, hematological malignancies, immune checkpoints, immune evasion, macrophage polarization, tumor immune microenvironment

## Abstract

The progressive cross-talk between tumor cells and the immune microenvironment, which is required for tumor growth, progression, and dissemination, has been well documented in solid cancers and is increasingly recognized in hematological malignancies. Extracellular vesicles (EVs) are now emerging as essential facilitators of immune-tumor crosstalk. In hematological cancers, EVs from leukemia, lymphoma, and myeloma cells carry oncogenic proteins, lipids, cytokines, and non-coding RNAs that reprogram immune cells and establish tumor-promoting immune states, in which tumor cells are protected from immune elimination and therapy-induced cell death. Evidence shows that tumor-derived EVs impair antitumor immunity, in part, by promoting macrophage M2 polarization, myeloid-derived suppressor cell (MDSC) expansion, dendritic cell maturation, T-cell exhaustion, and NK-cell cytotoxicity. Tumor cell-derived EV cargo, such as PD-L1, TGF-β, IL-10, and tumor-encoded miRNAs, creates highly immunosuppressive niches that promote disease progression and are associated with a dismal prognosis. On the other hand, immune-cell–derived or engineered EVs can activate CD4^+^ and CD8^+^ T cells, augment NK-cell killing, and serve as highly potent vehicles for antigen delivery or immune checkpoint modulation. By modifying the EV cargo, blocking EV uptake, or using EVs as vaccines or targeted delivery vehicles, it is now possible to restore antitumor immunity and reverse immunotherapy resistance. This review will provide an update on the current understanding of EVs in the regulation of hematological cancers, from mechanistic, translational, and therapeutic perspectives. We will also discuss the potential of using EVs as biomarkers and as next-generation immunotherapeutic agents.

## 1 Introduction

Hematologic malignancies, leukemia, multiple myeloma, non-Hodgkin lymphoma, and Hodgkin lymphoma are some of the most common cancers worldwide. The incidence of these malignancies is increasing; however, the number of deaths is decreasing (Pham et al., 2021). From 1990 to 2019, the annual number of cases doubled to more than 1.3 million, attributed to population growth and aging (Zhang et al., 2023). The treatment landscape for hematological malignancies is changing rapidly and now includes a variety of targeted and immune-based therapies in addition to conventional chemotherapy (Sochacka-Ćwikła et al., 2022). Precision medicine is revolutionizing the care of patients with hematologic cancers by tailoring treatment to the molecular and genetic profile of each patient, moving beyond the traditional “one-size-fits-all” approach (Morales-Martínez and Vega, 2022). Cancer cells need to communicate in a bidirectional manner with the surrounding immune and stromal cells to promote their survival, escape immune surveillance, and acquire resistance to therapy (Khalaf et al., 2021). Among the regulators of this complex crosstalk, extracellular vesicles (EVs), heterogeneous cell-derived membrane-bound particles including exosomes and microvesicles, have emerged as major players in tumor–immune interactions (Di Bella, 2022; Magalhães-Gama et al., 2024). EVs are abundantly released by hematological cancer cells and horizontally transfer their bioactive cargo of proteins, lipids, DNA, and non-coding RNAs to recipient cells (Song et al., 2025). Antitumor immunity can be inhibited by tumor-derived EVs delivering immune checkpoint molecules (e.g., PD-L1), pro-apoptotic ligands (e.g., FasL), or immunosuppressive cytokines and miRNAs that block T cell activation, dampen NK cell cytotoxicity, and induce expansion of regulatory T cells and myeloid-derived suppressor cells (Yim et al., 2020). In contrast, EVs from dendritic cells, NK cells, and cytotoxic T lymphocytes can stimulate antitumor responses by transferring antigen-MHC complexes, cytotoxic proteins, or immune-stimulatory RNAs to target cells (Wang et al., 2025).

EVs have been mechanistically described to play a role in cancer pathophysiology; more recently, their value as potential biomarkers and tools for therapy is being defined (Ciferri et al., 2021). Circulating EVs harboring immunomodulatory cargoes (PD-L1, miRNAs, cytokine transcripts, and other proteins) associate with disease burden and patient prognosis (Liu J. et al., 2022). In parallel, both engineered EVs as well as immune-cell–derived vesicles are under evaluation in multiple immune-oncology settings, from cancer vaccines to drug delivery and drug conjugates, as well as synergistic agents with CAR-T cells (Calvo and Izquierdo, 2022). These advances highlight the dual role of EVs as both facilitators of immune escape and promising therapeutic candidates.

In this review, we will summarize our current understanding of immune cell–EV interactions in hematological malignancies. In particular, we will focus on how EV signaling orchestrates the immune landscape (impact of EVs on immune subsets, including macrophages/monocytes, T cells, NK cells, DCs, B cells, and MDSCs), promotes tumor immune escape, and harbors prognostic and therapeutic implications. We will aim to cohesively integrate mechanistic and translational knowledge into a framework that will allow us to understand better EV-immune cell crosstalk and their therapeutic potential in hematological cancers.

## 2 Extracellular vesicle heterogeneity and nomenclature

EVs comprise a heterogeneous population of lipid-bilayer-delimited particles released by cells that cannot replicate independently. EV heterogeneity arises from differences in cellular origin, biogenesis, size, density, molecular composition and physiological context (Di Bella and Taverna, 2024). This heterogeneity is particularly relevant in hematological malignancies because plasma, bone marrow and conditioned-medium preparations may contain EVs released by malignant cells, immune cells, stromal cells, platelets and other host cells. These populations may carry distinct proteins, lipids and nucleic acids and can consequently exert different, or even opposing, immunological effects (Van Morckhoven et al., 2023).

In accordance with the Minimal Information for Studies of Extracellular Vesicles 2023 (MISEV2023) (Welsh et al., 2024), the generic term “EV” is preferred unless the subcellular origin of a vesicle population has been convincingly demonstrated. Exosomes are defined by their endosomal origin: they develop as intraluminal vesicles within multivesicular bodies and are released when these compartments fuse with the plasma membrane. Ectosomes arise through budding from the plasma membrane. The term “microvesicle” has often been used for plasma membrane-derived EVs, large EVs, or even EVs in general and is therefore discouraged because of its inconsistent meaning. Because most separation methods do not isolate vesicles according to their biogenesis and no universal molecular markers distinguish exosomes from ectosomes, biogenesis-based terminology should be used cautiously (Welsh et al., 2024).

Operational terms based on measurable characteristics are preferable when vesicle origin cannot be established. Small EVs are often described as particles below 200 nm, whereas large EVs are often described as particles above 200 nm. However, these boundaries are not absolute, measured diameter depends on the characterization method, and EV populations recovered by common separation procedures frequently have overlapping size distributions. Small EVs and exosomes are therefore not synonymous: small-EV preparations may contain endosome-derived exosomes, small plasma membrane-derived ectosomes, and other vesicular or non-vesicular particles. Similarly, the detection of CD9, CD63, CD81, ALIX, or TSG101 does not independently establish an exosomal origin because these proteins are not universal or exclusive markers of exosomes (Welsh et al., 2024).

EV heterogeneity is further complicated by the presence of apoptotic EVs and non-vesicular extracellular particles (NVEPs). Apoptotic bodies and other apoptotic EVs originate from cells undergoing programmed cell death and may overlap in size and molecular composition with EVs released by viable cells. NVEPs, including lipoproteins, ribonucleoprotein complexes, protein aggregates, and other cell-derived particles lacking a delimiting lipid bilayer, may also co-separate with EVs. This issue is especially relevant to blood-derived samples, in which lipoproteins, platelets, soluble proteins, and protein–nucleic acid complexes can confound the attribution of a biomarker or biological function specifically to EVs (Welsh et al., 2024).

Commonly employed techniques, including differential ultracentrifugation, polymer precipitation, size-exclusion chromatography, density-gradient centrifugation, and affinity capture, generate EV-containing preparations with different balances between recovery and specificity. None of these methods alone necessarily produces a pure, biogenetically uniform EV population. Therefore, differences in sample collection, pre-analytical processing, separation, storage, and characterization may alter the apparent abundance, cargo, and biological activity of EVs and may partly explain inconsistencies among studies. In this review, “EV” is used as the default term when a study did not demonstrate a specific biogenesis pathway. Subtype-specific terms reported by the original investigators are retained only when necessary and are qualified where the supporting experimental evidence is insufficient (Welsh et al., 2024). The MISEV2023-aligned definitions and recommended terminology adopted throughout this review are summarized in Table 1.

**TABLE 1:** Recommended terminology table based on MISEV2023 guidelines.

Term	Meaning under MISEV2023	Recommended use
Extracellular vesicle	Cell-released, lipid-bilayer-delimited, non-replicating particle	Preferred generic term
Small EV	Operational size category, often <200 nm	Acceptable if the size range and method are stated
Large EV	Operational size category, often >200 nm	Acceptable if the size range and method are stated
Exosome	EV originating from the endosomal system	Use only when origin is demonstrated
Ectosome	EV originating from the plasma membrane	Use only when origin is demonstrated
Microvesicle	Historically inconsistent, usually implying plasma membrane origin	Generally discouraged
Apoptotic EV/body	EV released during programmed cell death	Use when donor-cell condition or formation is supported
NVEP	Extracellular particle lacking a delimiting lipid bilayer	Recommended for non-vesicular particles
Extracellular particle	Umbrella term encompassing EVs and NVEPs	Use when vesicular nature is uncertain
EV preparation	Material enriched in EVs but potentially containing other particles	Appropriate for incompletely purified samples

## 3 Macrophages, EVs, and hematological malignancies

In the context of cancer, there exists a mutual crosstalk between EVs and macrophages. In the tumor microenvironment, cancer cells secrete EVs loaded with bioactive cargoes, including microRNAs, long noncoding RNAs, proteins, and lipids, which are then recognized and taken up by macrophages, leading to their polarization mainly towards an immunosuppressive and tumor-promoting M2-like phenotype. Subsequently, these EV-educated TAMs, by secreting cytokines, chemokines, growth factors, and matrix-remodeling enzymes, further promote angiogenesis, tumor growth, invasion, and metastasis as well as impair anti-tumor immunity (Pan et al., 2020). In turn, macrophage-derived EVs may reciprocally target tumor cells to either facilitate tumor cell proliferation, survival, and drug resistance by transferring oncogenic miRNAs and other signaling molecules or to suppress tumor progression. However, this is less well documented and usually occurs with EVs derived from classically activated M1-polarized macrophages. This bidirectional crosstalk between EVs and macrophages forms a dynamic communication loop through which cancer cells and TAMs reinforce each other to facilitate an immunosuppressive tumor microenvironment, and is also a potential therapeutic target to reprogram macrophages or block EV-mediated signaling in anti-tumor immunotherapy (Tang et al., 2022). This section aims to delineate the bidirectional crosstalk between macrophages and extracellular vesicles in hematological malignancies, highlighting how tumor- and macrophage-derived EVs reprogram immune function, remodel the tumor microenvironment, and influence disease progression, immune evasion, and therapeutic vulnerability (Figure 1).

**FIGURE 1:**
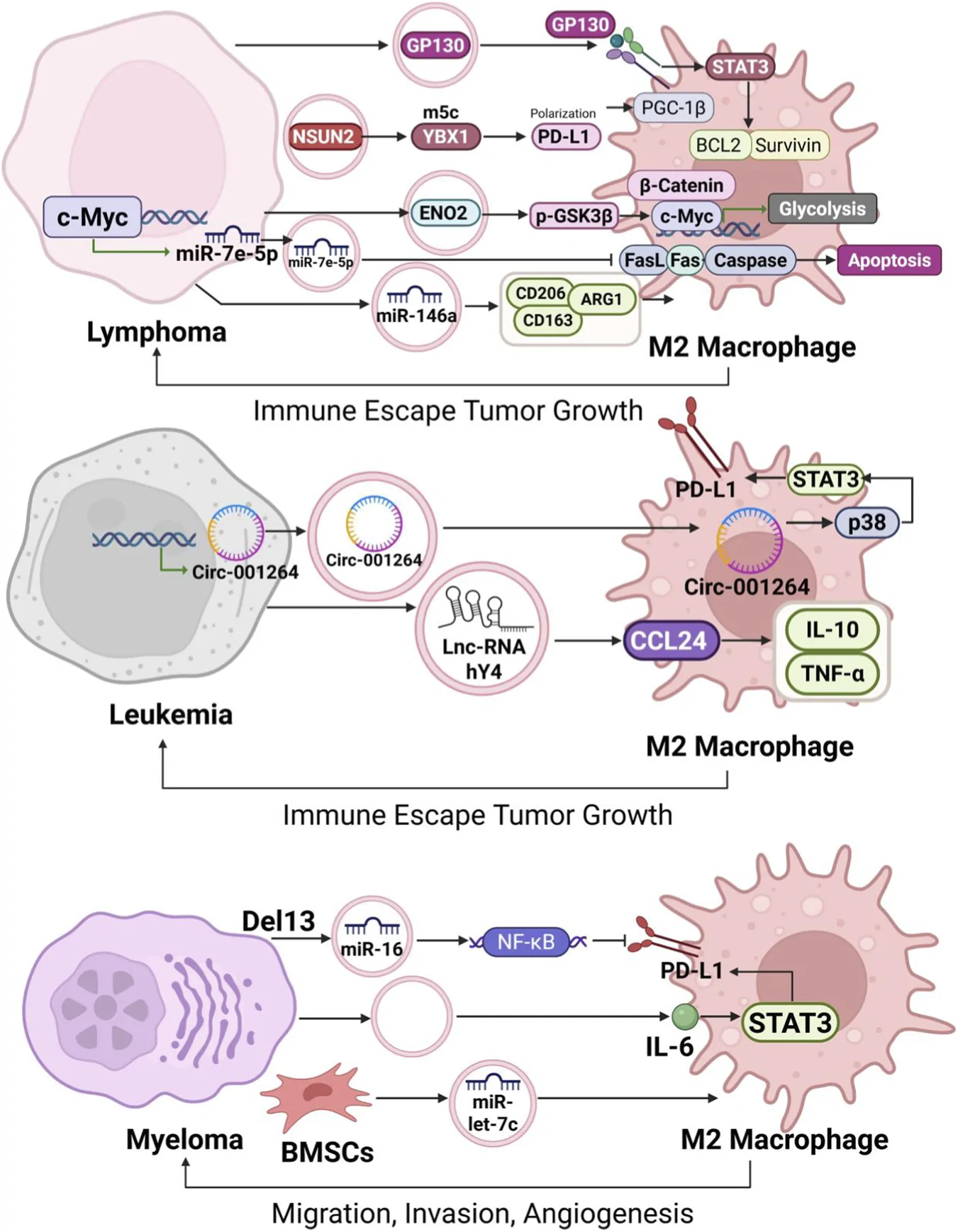
Crosstalk between cancer cell-derived EVs and macrophages in hematological malignancies. EVs released from lymphoma, leukemia, and multiple myeloma cells transfer diverse bioactive cargos, including proteins, miRNAs, circRNAs, and lncRNAs, to macrophages, reprogramming their metabolism, survival, and polarization toward an immunosuppressive M2 phenotype. These EV-mediated pathways, involving NSUN2/YBX1/PD-L1, GP130/STAT3, GSK3β/β-catenin/c-Myc, p38–STAT3, NF-κB, and IL-6 signaling, promote immune evasion, tumor growth, angiogenesis, and therapy resistance, thereby shaping a tumor-supportive microenvironment.

### 3.1 Crosstalk of cancer cell-derived EVs with macrophages

#### 3.1.1 Lymphoma cells

Diffuse large B-cell lymphoma (DLBCL) cell-derived EV preparations transferred NSUN2 to recipient tumor cells, where it promoted YBX1-dependent stabilization of PD-L1 mRNA through m5C modification. Increased PD-L1 expression was associated with CD8^+^ T-cell apoptosis, reduced TNF-α and IFN-γ secretion, CD206^+^ macrophage polarization, and tumor growth. PD-L1 silencing attenuated these effects, supporting the proposed NSUN2–YBX1–PD-L1 pathway. The particles were isolated by ultracentrifugation and characterized using TEM and EV-associated proteins without demonstrating endosomal origin. Moreover, the absence of particle quantification, purity controls, and patient-derived EV validation limits the clinical interpretation of these findings (Ling et al., 2025). Tumor-derived exosomal ENO2 contributes to the reprogramming of macrophage metabolism in the DLBCL tumor microenvironment. The uptake of DLBCL-released ENO2-enriched EVs into macrophages causes increased glycolysis in macrophages by the activation of GSK3β/β-catenin/c-Myc pathway. This leads to macrophages skewing towards the immunosuppressive M2-like phenotype through an upregulation of IL-10, TGF-β, and CD206 expression, and a suppression of M1 marker genes. M2-polarized tumor-associated macrophages (TAMs) then promoted tumor proliferation, migration, and invasion, as well as stimulated lymphoma cell immune evasion, leading to accelerated disease progression in DLBCL. However, its endosomal origin was not demonstrated, and the clinical findings were based on a relatively small cohort without independent validation (Shao et al., 2024). DLBCL-derived EVs can be phagocytosed by macrophages, which deliver GP130 and activate STAT3 signaling pathways. STAT3 upregulated anti-apoptotic proteins BCL2 and SURVIVIN and downregulated pro-apoptotic BAX, thereby enhancing macrophage survival. In addition, STAT3 activation polarized macrophages into an immunosuppressive, tumor-promoting M2-like phenotype, leading these EV-educated macrophages to secrete IL-10, IL-6, CD163, and CD206. The net effects of these mechanisms include immune evasion, drug resistance, and lymphoma progression. Notably, STAT3 was inhibited by GP130-blocking antibody SC144, which blocks M2 polarization. Therefore, GP130/STAT3 signaling activation can be considered a mechanism by which DLBCL-derived EVs reprogram the tumor microenvironment, and it is also a potential therapeutic target for this disease. The evidence was limited to cell models, and the precipitation-based isolation method with limited EV characterization did not establish exosomal origin or exclude non-vesicular (Ling et al., 2022). In a similar study, DLBCL–derived EVs also reprogrammed tumor-associated macrophages within the microenvironment, pushing them towards an M2, tumor-promoting phenotype. The mechanism involved EVs driving macrophages toward an M2 phenotype by upregulating oxidative phosphorylation, a process controlled by PGC-1β, a major transcriptional driver of mitochondrial biogenesis and function. PGC-1β levels were elevated by EVs, which met the metabolic requirements for M2 polarization and, in turn, led to increased secretion of immunosuppressive cytokines such as IL-10 and CCL-18 and decreased production of pro-inflammatory cytokines and chemokines. These EV-educated macrophages released various soluble factors that induced proliferation, migration, and invasion of lymphoma cells, all of which worked together to accelerate immune evasion and disease progression In contrast to the ENO2 study, which implicated increased glycolysis, this study linked M2 polarization to oxidative metabolism. However, the evidence was limited to *in vitro* models and requires *in vivo* and clinical confirmation (Liu et al., 2020). In pediatric ALK + anaplastic large cell lymphoma (ALCL), tumor cells released small extracellular vesicles (s-EVs) that were highly enriched in miR-146a-5p in relapsed patients. MiR-146a-5p was functionally important for the tumor microenvironment, as shown by functional studies that s-EVs could efficiently transfer miR-146a-5p to macrophages to increase their migration towards tumor cells and M2 polarization marked by CD163, CD206, and Arginase-1 expression. In return, these tumor-educated macrophages secreted pro-tumorigenic cytokines, including IL-8, which promoted ALCL cell migration, clonogenicity, and aggressiveness. High level of s-EVs–derived miR-146a-5p was associated with worse prognosis and lower progression-free survival, making it both a potential biomarker for relapse risk and a functional miRNA that reprogrammed macrophages to infiltrate and differentiate into M2 subtype to drive disease progression in pediatric ALK + ALCL. The clinical cohort strengthens the evidence; however, sEV treatment did not increase CD206 or ARG1, and the prognostic findings require independent validation (Garbin et al., 2023). The downregulation of miR-7e-5p, caused by c-MYC overexpression, is important for the transformation of indolent follicular lymphoma (FL) into its aggressive counterparts, high-grade FL and diffuse large B-cell lymphoma (DLBCL). FL cells normally transferred miR-7e-5p to macrophages via exosomes, which targeted and suppressed FasL expression, thereby maintaining macrophage immune surveillance. The downregulation of miR-7e-5p leads to upregulation of FasL expression in M1 macrophages, activating caspase signaling and promoting apoptosis, thereby reducing macrophage antitumor immunity and contributing to disease progression. In a clinical setting, lower levels of miR-7e-5p correlated with poorer clinical outcomes, suggesting that it could be a potential therapeutic target to preserve M1-like macrophages and delay FL transformation. (Lou et al., 2020). Unlike the preceding studies, disease progression resulted from loss of an immunoprotective EV cargo rather than enrichment of a tumor-promoting cargo. Clinical tissue findings supported the mechanism, but the small, heterogeneous cohorts and predominantly murine *in vitro* experiments require further validation. CD169^+^ macrophages play an important role in maintaining immune responses to tumor-derived apoptotic vesicles (ApoVs). CD169 is a type I transmembrane glycoprotein that was highly expressed on macrophages in the spleen and lymph nodes. As expected, CD169 macrophages preferentially captured sialic acid–rich ApoVs, which were derived from the apoptosis of EL4 lymphoma cells, and shuttled them into their intracellular compartments, where they were degraded and prevented excessive inflammation. However, in a CD169-deficient mouse model, the cytotoxic T-cell (CTL) response to ApoV-associated tumor antigens was significantly boosted, suggesting that CD169 normally worked to keep the CTL response against apoptotic tumor components at bay and promote tolerance. In addition, even though ApoVs pulsed with tumor antigens could activate CTL responses, unpulsed ApoVs from tumor cells could also suppress dendritic cell-induced immunity in a CD169-independent. These findings demonstrate context-dependent immune effects but are limited to an artificial murine ovalbumin model without human validation (Black et al., 2016). Collectively, these studies support the ability of lymphoma-derived EVs) to alter macrophage survival, metabolism, and polarization, although the quality of evidence varies. The NSUN2, ENO2, and GP130 studies provide stronger mechanistic evidence through cargo manipulation or pathway inhibition, whereas the PGC-1β findings remain largely limited to *in vitro* models. Notably, ENO2-containing EVs promoted glycolysis, while another study linked macrophage polarization to increased oxidative phosphorylation. This difference may reflect distinct lymphoma models or macrophage states, but direct comparative studies are lacking. Clinical associations reported for miR-146a-5p and miR-7e-5p are promising but require validation in larger independent cohorts. Most studies used ultracentrifugation or precipitation and EV-associated markers without demonstrating endosomal origin; therefore, “EVs,” rather than “exosomes,” is more appropriate. The apoptotic-vesicle study provides stronger *in vivo* evidence but used an artificial murine antigen model. Overall, reproducibility is limited by heterogeneous isolation methods, incomplete exclusion of non-vesicular components, and simplified M1/M2 classification.

#### 3.1.2 Leukemia cells

K562 chronic myeloid leukemia (CML) cell-derived EVs altered cytokine production and redox activity in bone marrow mesenchymal stromal cells and macrophages. In macrophages, EV treatment increased interleukin-10 and tumor necrosis factor-α while reducing inducible nitric oxide synthase expression, nitric oxide production, and reactive oxygen species, suggesting a tumor-supportive phenotype. However, the simultaneous increase in pro- and anti-inflammatory cytokines indicates a mixed activation state rather than classical M2 polarization. The study was limited to one leukemia cell line, used human stromal cells but murine macrophages, and did not identify the responsible EV cargo or provide *in vivo* confirmation. Moreover, endosomal origin was not demonstrated; therefore, “EVs” is more appropriate than “exosomes” (Jafarzadeh et al., 2019). B-cell receptor (BCR) stimulation increased EV release from primary chronic lymphocytic leukemia (CLL) cells and altered their RNA cargo, particularly miR-146a-5p, miR-132-3p, miR-155-5p, and hY4 RNA. These EVs were preferentially internalized by monocytes and induced morphological, transcriptional, and secretory changes resembling nurse-like cells (NLCs). EV-treated monocytes secreted leukemia-supporting cytokines, protected CLL cells from spontaneous apoptosis, and promoted the migration of CLL cells and other monocytes. RNA degradation reduced these effects, while hY4 gain- and loss-of-function experiments supported its contribution to CCL24 expression. The use of primary CLL samples, multiple functional assays, and validation in 25 samples strengthens the evidence. However, the experiments were performed *in vitro*, sequencing included only four samples, and NLC differentiation was inferred mainly from molecular and morphological similarities. Moreover, endosomal origin was not demonstrated; therefore, the term “EVs” remains appropriate (Dubois et al., 2025). Acute myeloid leukemia (AML) cell-derived EVs transferred circular RNA circ_001264 to macrophages, activating the miR-502-5p/RAF1/p38–STAT3 pathway and promoting an M2-like phenotype and programmed death-ligand 1 expression. The proposed mechanism was supported by gain- and loss-of-function experiments, RNA pull-down, reporter assays, patient samples, and an animal model. However, the clinical analysis was observational, the animal groups included only three mice, and the use of highly immunodeficient mice limits interpretation of immune-checkpoint therapy. A published corrigendum and unclear claims regarding “secreted” programmed death-ligand 1 also warrant cautious interpretation (Du et al., 2023).

#### 3.1.3 Multiple myeloma

Hypoxia increased heparanase (HPSE) in multiple myeloma (MM) cell-derived EVs, which enhanced RAW264.7 macrophage migration and invasion. An HPSE-neutralizing antibody reduced macrophage invasion, supporting a functional role for EV-associated HPSE. However, the study used an engineered myeloma cell line and evaluated migration rather than macrophage polarization. It also lacked patient samples and *in vivo* confirmation, while EV characterization was limited mainly to particle size and concentration (Tripathi et al., 2023). MM cell-derived EVs reprogram macrophages to an immunosuppressive, tumor-promoting M2-like phenotype. Using exosomes isolated from the U266B1 myeloma cell line, researchers showed that incubating resting macrophages (M0) with these vesicles resulted in upregulation of M2 markers (Arg-1, IL-10, and CD206) and increased IL-10 secretion. In contrast, M1 markers such as TNF-α and markers of ROS and nitric oxide production were downregulated. Nevertheless, the evidence was limited to two independent *in vitro* experiments using one myeloma and one monocytic cell line. No responsible EV cargo or signaling pathway was identified, and ultracentrifugation did not exclude co-isolated non-vesicular components. (Razi et al., 2023). Similarly, small EVs (SEVs) released from MM cells also reprogrammed non-polarized macrophages (M0) into an immunosuppressive tumor-supportive phenotype. MM-derived SEVs were efficiently taken up by macrophages and strongly increased PD-L1 and IL-6 expression at both mRNA and protein levels. Mechanistically, the SEVs activated the IL-6/STAT3 pathway, driving the induction of PD-L1 via a STAT3-dependent autocrine loop, and the TLR4/NF-κB pathway, particularly due to SEV-associated HSP70 (Pucci et al., 2021). Unlike the U266 study, these findings indicate a pro-inflammatory state rather than conventional M2 polarization. This difference may reflect variation in myeloma cell lines, EV cargo, exposure duration, or the limited M1/M2 classification. Pathway analysis strengthened the mechanism, but the study remained restricted to THP-1-derived macrophages without patient or *in vivo* validation. Bone marrow mesenchymal stem cells (BMSCs) can secrete EV-derived miR-let-7c, which is taken up by bone marrow macrophages, inducing them to polarize towards the M2 phenotype. M2 macrophages have pro-angiogenic activity and can secrete various pro-angiogenic factors that are able to promote proliferation, migration, and tube formation of vascular endothelial cells. In addition to indirectly modulating angiogenesis through M2 macrophage polarization, miR-let-7c can directly induce angiogenesis in myeloma vascular endothelial cells. These events together help to construct a pro-angiogenic niche that favors the progression of multiple myeloma. Primary patient-derived cells strengthen the biological relevance, but only seven patients were included. Moreover, miR-let-7c was mainly manipulated directly in recipient cells rather than specifically depleted from donor EVs, weakening attribution to EV-mediated transfer (Tian et al., 2021). EVs and their cargo are critical regulators of the crosstalk between MM cells and BM macrophages. MM cells, which generally have an intact chromosome 13, secrete EVs enriched in miR-16, which directly target the IKKα/β complex and suppress NF-κB activation, thereby impairing monocyte differentiation into tumor-supportive M2 macrophages and limiting the secretion of the pro-inflammatory cytokines IL-6, IL-8, and TNF-α. In contrast, MM cells with a chromosome 13 deletion (Del13) produce EVs with low miR-16, which sustain NF-κB activation, promote M2 macrophage polarization, and establish a more tolerogenic, tumor-supportive microenvironment. This miR-16 deficiency in EVs secreted by MM cells with Del13 also confers greater MM progression and drug resistance, and this finding may be therapeutically exploited by restoring miR-16 levels to reprogram the BM niche and sensitize myeloma cells to proteasome inhibitors such as bortezomib. Evidence was strengthened by multiple cell lines, primary patient samples, target-validation assays, and a miR-15a/16-1 knockout mouse model. However, several experiments used direct miR-16 transfection rather than EV-specific manipulation, and patient comparisons included small groups. (Khalife et al., 2019). These studies support EV-mediated regulation of macrophages in MM but do not show a uniform M2 response. U266-derived EVs promoted M2-like markers without increasing interleukin-6, whereas MM1.S-derived EVs increased interleukin-6 and other inflammatory mediators. Furthermore, miR-16 acted as an inhibitory EV cargo, demonstrating that EV effects depend on donor-cell genotype and cargo rather than being universally tumor-promoting. The HPSE study assessed macrophage migration rather than polarization, and the miR-let-7c study examined stromal-cell-derived EVs. Evidence is strongest for the miR-16 mechanism, although EV-specific causality remains incomplete. Differences in cell models, isolation methods, treatment doses, and polarization markers limit direct comparison and reproducibility. Since none of these studies demonstrated endosomal origin, “EVs” is more appropriate than “exosomes.”

### 3.2 Crosstalk of macrophage-derived EVs with cancer cells

EVs derived from nurse-like cells (NLCs) and M2-polarized macrophages are internalized by CLL B-cells, where they decrease apoptosis, promote proliferation, and increase resistance to the Bruton’s tyrosine kinase inhibitor Ibrutinib. Mechanistically, EVs upregulate the anti-apoptotic protein BCL-2 and the proliferation-inducing factor April, and increase levels of IGFBP-2, CD40, and p53, all known to be involved in survival signaling. Transcriptomic and proteomic studies identified oncogenic proteins carried by EVs and CLL-B-cell gene expression alterations that reprogrammed cells towards survival and drug resistance within the tumor microenvironment (Ikhlef et al., 2024). However, NLC EVs were pooled, some residual CLL-cell contamination was possible, and transcriptomic and protein analyses included only three patients. Moreover, the responsible EV cargo was not functionally established, and no *in vivo* validation was performed. The particles should therefore be described as EVs because ultracentrifugation, NTA, and EV-associated proteins did not demonstrate endosomal origin. M1 macrophage-derived EVs produced the opposite effect in murine AML cells, suppressing proliferation and inducing mitochondrial dysfunction and apoptosis through phosphoglycerate mutase family member 5 (PGAM5). PGAM5 knockdown weakened these effects *in vitro* and in mouse models, providing stronger mechanistic evidence. Nevertheless, the study used only RAW264.7 and C1498 cells, lacked human validation, and injected EV-pretreated leukemia cells rather than treating established leukemia with EVs. Differential ultracentrifugation, TEM, and EV-associated proteins confirmed an EV-enriched preparation but not endosomal origin (Li et al., 2024). Together, these studies indicate that macrophage-derived EV effects depend on macrophage phenotype: M2-like/NLC EVs support leukemia survival, whereas M1 EVs promote leukemia-cell death. Evidence for the PGAM5 mechanism is comparatively stronger because it includes knockdown and animal experiments, while the NLC findings are clinically relevant but remain preliminary. Both conclusions require validation using primary human cells, standardized EV characterization, and *in vivo* EV-treatment models.

### 3.3 Comparative analysis across hematological malignancies

Across lymphoma, leukemia and multiple myeloma, tumor- or stromal-cell-derived EVs commonly reprogram macrophage survival, recruitment, metabolism and cytokine production toward tumor-supportive functions. STAT3, NF-κB, IL-6/IL-10 and PD-L1 emerged as recurrent downstream mediators. However, the resulting macrophage states were not uniformly M2-like, and several studies reported mixed inflammatory phenotypes or protective EV cargo, indicating that the M1/M2 framework provides only a simplified description (Ling et al., 2025; Shao et al., 2024; Ling et al., 2022; Liu et al., 2020; Garbin et al., 2023; Lou et al., 2020; Black et al., 2016; Jafarzadeh et al., 2019; Dubois et al., 2025; Du et al., 2023; Tripathi et al., 2023; Razi et al., 2023; Pucci et al., 2021; Tian et al., 2021; Khalife et al., 2019). The apparent disease-specific patterns were broader metabolic and survival reprogramming in lymphoma, nurse-like-cell differentiation and checkpoint regulation in leukemia, and bone-marrow-, hypoxia- and genotype-dependent signaling in MM. These distinctions remain provisional because direct comparisons are unavailable. Evidence for the reciprocal macrophage-to-tumor EV pathway was concentrated in leukemia, where NLC/M2-derived EVs supported CLL survival, whereas M1-derived EVs suppressed AML cells (Ikhlef et al., 2024; Li et al., 2024). Overall, EV effects depend more on donor-cell state, cargo and recipient context than on malignancy type alone. Predominantly preclinical models and heterogeneous EV methods further limit conclusions regarding genuinely shared or disease-specific mechanisms.

## 4 NK cells, EVs, and hematological malignancies

EVs released by cancer cells (TDEs) can activate or suppress NK cells, but overall, they are immunosuppressive. Although EVs presenting heat shock proteins or cytokines can trigger NK cell cytotoxicity, most TDEs contain inhibitory signals (e.g., NKG2D ligands and TGF-β1) that downregulate activating receptors, decrease perforin/granzyme release, and hamper NK proliferation and migration. TDEs thus promote local and systemic tumor immune escape (Batista et al., 2021). In this subsection, we summarize the interaction of EVs and NK cells in various hematological malignancies (Figure 2).

**FIGURE 2:**
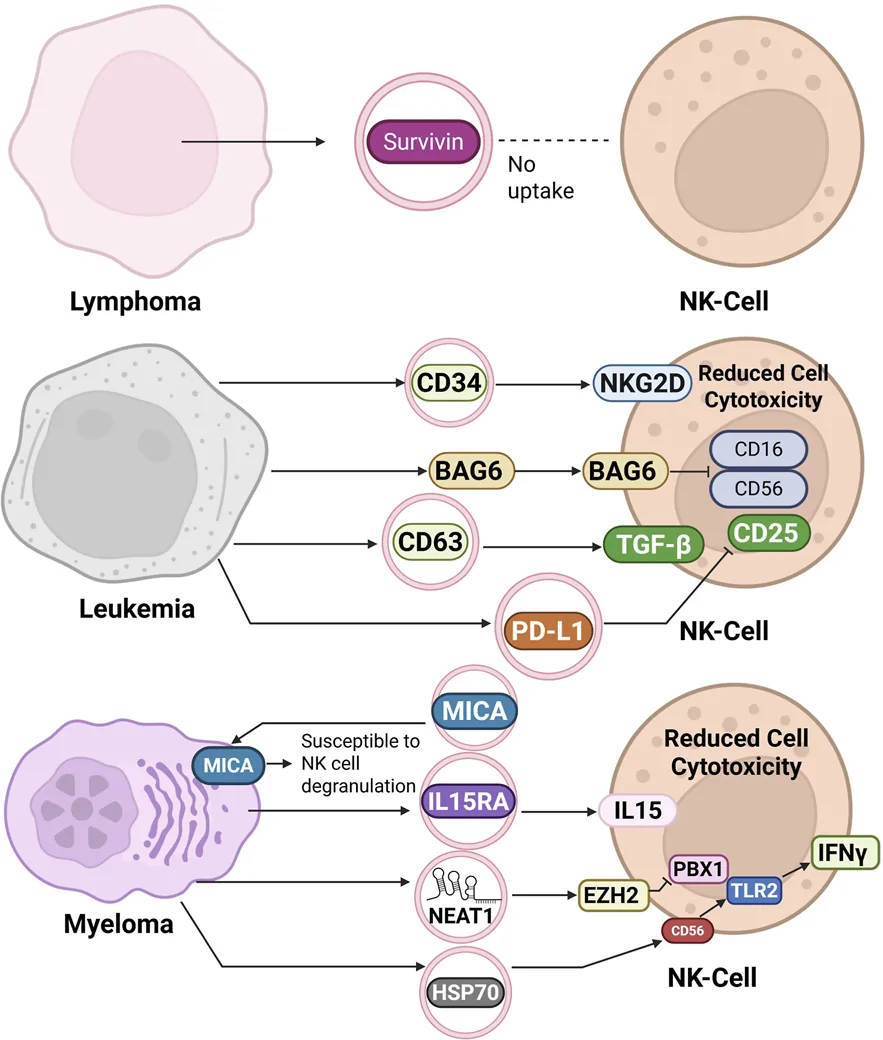
Cancer cell–derived EVs exert context-dependent but predominantly immunosuppressive effects on NK cells across lymphoma, leukemia, and multiple myeloma. In lymphoma, survivin-positive EVs show minimal uptake by NK cells and limited functional impact. In leukemia, EVs carrying CD34, BAG6, CD63, and PD-L1 suppress NK cell activation and cytotoxicity through downregulation of activating receptors such as NKG2D and NKp30, reduction of CD16/CD56 expression, and engagement of PD-1/PD-L1 and TGF-β signaling, collectively promoting immune escape. In multiple myeloma, EVs display dual effects: transfer of NKG2D ligands such as MICA enhances NK susceptibility to degranulation, whereas EV-associated lncRNA NEAT1 suppresses NK proliferation and effector function via the EZH2/PBX1 axis. Chemotherapy-induced EVs enriched in HSP70 or IL15/IL15RA complexes can selectively stimulate NK cytokine production or proliferation without fully restoring cytotoxic degranulation.

### 4.1 Lymphoma cells derived EVs affecting NK cells

Lymphoma-derived EVs from aggressive DLBCL and indolent follicular lymphoma cell lines contained survivin, Fas ligand, and other inhibitor-of-apoptosis proteins. However, exposure to these EVs did not significantly alter NK cell NKG2D expression, degranulation, cytotoxic protein levels, or killing activity. In contrast, recombinant survivin reduced NKG2D, perforin, granzyme B, TNF-α, and IFN-γ protein levels without changing their transcripts, degranulation, or cytotoxicity. Thus, the findings do not establish EV-associated survivin as an inhibitor of NK-cell function. The discrepancy may reflect insufficient survivin delivery, limited EV uptake by NK cells, or the absence of TGF-β and MICA/B from these EVs. Evidence remains limited using 2 cell lines, small donor samples, and a Fas-negative K562 cytotoxicity model that could not assess Fas ligand-mediated effects. Moreover, ExoQuick precipitation has low specificity and may co-isolate soluble proteins; therefore, the preparations should be described as EVs rather than exosomes because endosomal origin was not demonstrated (Ferguson Bennit et al., 2021).

### 4.2 Leukemia cells derived EVs affecting NK cells

In CLL, BCL2-associated athanogene 6 (BAG6) was predominantly released as a soluble protein rather than in EVs. Soluble BAG6 and NKG2D ligands increased with disease stage and reduced NK-cell cytotoxicity and CD16/CD56 expression. Conversely, BAG6-positive EVs from stressed non-CLL cells activated NK cells, while BAG6 depletion promoted immune escape *in vitro* and in mice. The study combined patient plasma, genetic manipulation, and animal experiments, providing relatively strong mechanistic evidence. However, only six patient samples underwent functional fractionation, and the activating EVs originated mainly from engineered non-CLL cells. Thus, the findings support an altered soluble-to-EV BAG6 balance rather than direct suppression by CLL-derived EVs (Reiners et al., 2013). In AML, immunoaffinity capture identified CD34-positive EVs carrying CD33, CD117, CD44, CD200, and CD105, resembling their parental blasts. Their abundance correlated with circulating CD34-positive blasts. The captured EVs reduced NKG2D expression on NK cells, whereas the CD34-negative fraction mainly reduced NKp46, suggesting that different EV populations affect distinct NK-cell receptors. Patient-derived samples and comparison with uncaptured EVs strengthen the evidence. However, CD34 capture excludes CD34-negative AML, may also recover EVs from normal progenitors, and receptor downregulation alone does not establish impaired NK-cell killing (Hong et al., 2014). THP-1-derived EVs carried PD-L1 and reduced NK-cell CD25 expression and cytotoxicity. Pretreatment with the anti-PD-L1 antibody atezolizumab or the PD-1/PD-L1 antagonist AUNP-12 partly restored NK-cell activity, supporting involvement of EV-associated PD-L1. This effect was observed in NKL cells and primary peripheral blood cells, but the evidence remains preclinical because only one AML cell line was used. Cytotoxicity was assessed indirectly by cell viability and counting, and genetic depletion of EV-associated PD-L1 was not performed. Patient-derived AML EVs and *in vivo* confirmation are therefore required (Wang D. et al., 2024). EVs from Jurkat T-cell acute lymphoblastic leukemia cells were internalized by NK92-MI cells and reduced their proliferation, cytotoxicity, perforin, and granzyme B levels. A PCR array identified 18 altered genes within the TGF-β/BMP pathway, including marked upregulation of MECOM and HIPK2. However, pathway activation was inferred from gene expression without TGF-β receptor blockade or depletion of the responsible EV cargo. Furthermore, the study relied entirely on cell lines, used only CD63 for protein characterization, and did not report EV-depleted serum during production. These limitations make the proposed TGF-β mechanism preliminary and prevent generalization to other ALL subtypes (Yu et al., 2021). Together, leukemia-derived EVs can alter NK-cell activity through distinct mechanisms, including receptor modulation, PD-L1 signaling, and possible TGF-β pathway activation. Evidence is strongest for the BAG6 soluble-versus-EV balance and for PD-L1 involvement because these studies included genetic or pharmacological interventions. The CD34-positive EV findings are clinically relevant but limited to a selected AML population, while the T-ALL mechanism requires further confirmation. Differences between studies likely reflect leukemia subtype, EV composition, recipient NK-cell model, and separation method.

### 4.3 Multiple myeloma cells derived EVs affecting NK cells

Genotoxic stress in MM cells induced by the chemotherapy drug melphalan is known to induce sEV release and modulate NK cells, but the interplay between these events remains unclear. Melphalan treatment further enriched MM cell sEVs for the heat shock protein 70 (HSP70) on their surface, which were then taken up by NK cells, leading to selective activation of IFNγ and cytokine production, but not cytotoxic degranulation, by an NK-TLR2/NF-κB signaling axis. The cytokine-producing CD56high NK cell subset particularly pronounced the response. Thus, the findings support selective cytokine activation rather than a complete antitumor NK response (Vulpis et al., 2017). In contrast, NEAT1-containing MM-derived EVs inhibited NK92-cell proliferation, promoted apoptosis, reduced NKG2D expression and suppressed TNF-α and IFN-γ production through the EZH2–PBX1 pathway. Knockdown and rescue experiments, together with xenograft findings, support a causal mechanism; however, the reliance on NK92 cells and limited functional evaluation of patient-derived EVs reduce the clinical strength of the evidence (Wang Q-M. et al., 2024). EVs also facilitate “cross-dressing” of MM cells with NKG2D ligands (NKG2DLs). MM cells take up NKG2DLs, including the MICA allelic variants MICA008, MICA019, and ULBPs, from EVs and express them on their surface to augment NK recognition. Both the GPI-anchored ligands MICA008, ULBP1, ULBP3, and the transmembrane ligands MICA019, ULBP2 were successfully transferred from EVs to MM cells in a clathrin- and dynamin-dependent endocytosis mechanism, with the GPI-anchored ligands being more efficiently transferred. Functionally, MM cells cross-dressed with these ligands were more susceptible to NK cell degranulation and lysis. MICA019 and ULBP1+ EVs were enriched in the plasma of the bone marrow of MM patients. This seemingly contradicts the established suppressive effects of EV-associated NKG2D ligands, but the recipient cell differed: ligand transfer to MM cells increased their immunogenicity, whereas direct and persistent ligand exposure of NK cells may downregulate NKG2D. Nevertheless, extensive use of ligand-overexpressing donor cells and heterogeneous 13,000 × g pellets limits generalizability (Vulpis et al., 2023). Senescent MM cells also released IL-15/IL-15Rα-containing sEV-enriched particles that supported NK-cell proliferation. However, the effect was modest without exogenous IL-15, and vesicles from drug-treated cells were not more active on an equal-dose basis than those from untreated cells. These results therefore support amplification of an existing IL-15 response rather than autonomous NK-cell activation (Borrelli et al., 2018). The discrepancies among these studies can be explained by differences in cargo, EV fraction, recipient cell and measured endpoint. NEAT1-containing EVs directly suppressed NK survival and activity, whereas HSP70-positive particles increased cytokine production without improving cytotoxicity, IL-15/IL-15Rα-positive particles promoted proliferation under cytokine-permissive conditions, and NKG2D-ligand-bearing mEVs acted indirectly by modifying MM cells. Therefore, cytokine production, proliferation and tumor-cell killing should not be interpreted as interchangeable indicators of NK activation.

Overall reproducibility remains limited. Three stimulatory studies used overlapping MM cell lines, experimental platforms and research settings, so they do not represent fully independent replication. The inhibitory NEAT1 pathway was reported using different models but remains a single mechanistic study. No study directly compared the different EV populations under standardized isolation, dosing and NK-cell assay conditions, leaving the dominant effect of MM-derived EVs unresolved.

### 4.4 Comparative analysis across hematological malignancies

Across lymphoma, leukemia and multiple myeloma, tumor-associated EVs influenced NK-cell activity by altering activating-receptor expression, proliferation, survival, cytokine secretion or cytotoxicity. The NKG2D axis was the most recurrent target, but its effects were not consistent across diseases. Direct exposure of NK cells to selected leukemia- or MM-derived preparations reduced NKG2D expression, whereas transfer of NKG2D ligands from EVs to MM cells increased tumor-cell recognition. These observations demonstrate that the same ligand system may promote either immune escape or immune recognition depending on the recipient cell, duration of exposure and EV context (Ferguson Bennit et al., 2021; Reiners et al., 2013; Hong et al., 2014; Wang D. et al., 2024; Yu et al., 2021; Vulpis et al., 2017; Wang Q-M. et al., 2024; Vulpis et al., 2023; Borrelli et al., 2018). The overall patterns also differed among malignancies. The available lymphoma study did not demonstrate meaningful suppression of NK-cell function despite the presence of potentially inhibitory EV cargo, leaving the biological importance of lymphoma-derived EVs unresolved (Ferguson Bennit et al., 2021). Leukemia-associated preparations generally produced more inhibitory effects through activating-receptor downregulation, PD-L1 signaling and possible TGF-β/BMP pathway modulation. However, the BAG6 study showed that NK regulation also depended on whether the immunoregulatory protein was soluble or EV-associated, rather than simply on its abundance (Reiners et al., 2013; Hong et al., 2014; Wang D. et al., 2024; Yu et al., 2021). MM displayed the greatest heterogeneity: some EV populations inhibited NK-cell survival and activity, whereas stress-associated preparations increased cytokine production or supported proliferation, and others acted indirectly by increasing MM-cell immunogenicity. These stimulatory responses did not consistently enhance cytotoxic degranulation or tumor-cell killing and therefore should not be interpreted as complete NK-cell activation (Vulpis et al., 2017; Wang Q-M. et al., 2024; Vulpis et al., 2023; Borrelli et al., 2018). Evidence strength remains uneven. The BAG6 and NEAT1 studies provide stronger mechanistic support through genetic manipulation and animal experiments, whereas the lymphoma findings and proposed T-ALL pathway remain preliminary. Patient-derived AML and MM samples increase clinical relevance, but most functional experiments relied on cell lines or selected EV fractions. Differences in EV isolation, dosing, recipient NK-cell model and assay endpoint further limit comparison. Accordingly, the available evidence supports cargo- and context-dependent NK regulation rather than a uniformly suppressive or stimulatory effect specific to any hematological malignancy.

## 5 CD4^+^ T cells

Tumor-derived EVs alter CD4^+^ T-cell responses to promote immune escape. By expressing FasL and galectin-9 on their surface, EVs can induce apoptosis of CD4^+^ Th1 cells, while the TGF-β contained in the vesicles and immunosuppressive miRNAs reprogram conventional CD4^+^ T cells into Tregs. CD4^+^ T-cell proliferation and cytokine secretion (IL-2, IFN-γ, IL-17) are also inhibited by tumor EVs, skewing T-cell responses away from antitumor Th1/Th17 immunity. Exosomal miRNAs (e.g., miR-214, miR-24-3p, miR-20a-5p) can also modify gene expression in different CD4^+^ subsets, reinforcing expression of immune inhibitory molecules, including TGF-β, IL-10, CD39, and CD73, to enhance Treg activity and extracellular adenosine (Czernek and Düchler, 2017). In this section, we discuss the interactions between CD4^+^ T cells and tumor-derived EVs in hematological malignancies (Figure 3).

**FIGURE 3:**
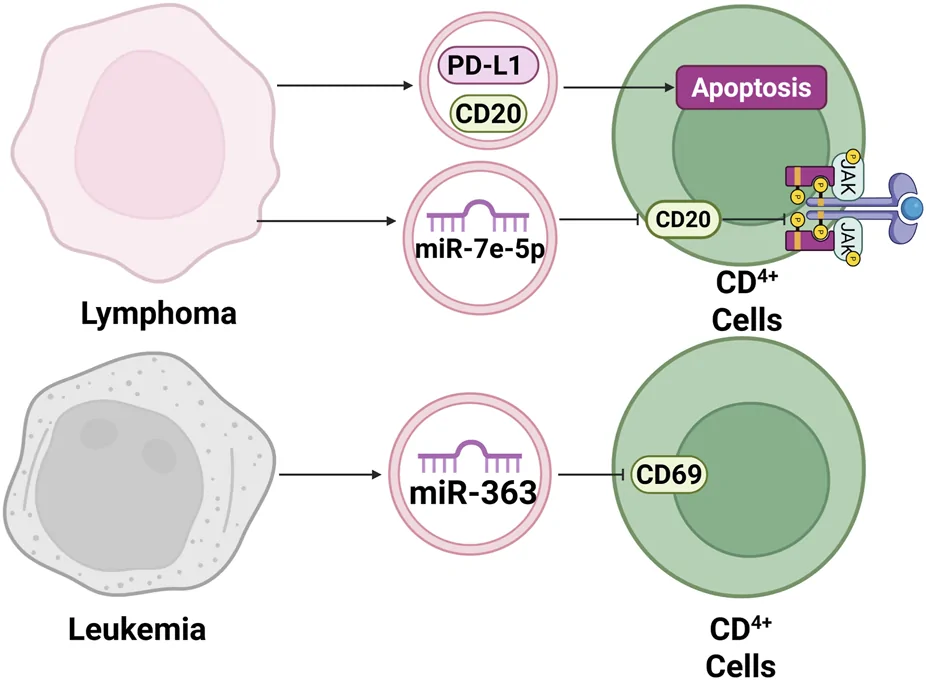
Tumor-derived EVs modulate CD4^+^ T-cell function in lymphoma and leukemia.In lymphoma, EVs enriched in CD20 and PD-L1 induce CD4^+^ T-cell apoptosis and suppress antitumor immunity, while EV-associated miRNAs contribute to immune escape. In leukemia, CLL-derived EVs selectively transfer miR-363 to CD4^+^ T cells, downregulating CD69 and reprogramming T-cell signaling and behavior toward tumor-supportive phenotypes.

### 5.1 Lymphoma

sEV-enriched preparations from DLBCL cell lines and EBV-transformed B cells contained CD20 and glycosylated PD-L1. The capacity of vesicular CD20 to bind anti-CD20 antibodies supports a potential decoy mechanism, whereas sEVs with high PD-L1 abundance induced apoptosis in activated CD4^+^ and CD8^+^ T cells. However, the functional experiments involved a limited number of cell models, and PD-L1 neutralization was not performed. Consequently, the T-cell apoptosis cannot be attributed exclusively to vesicular PD-L1 because other vesicular death signals may have contributed. In patient plasma, sEV concentrations were higher in DLBCL than in healthy controls, but CD20 was inconsistently detected and PD-L1 was abundant in both groups. These findings support a potential biological role for sEV-associated CD20 and PD-L1 but provide weak evidence for their disease specificity or biomarker value (Akil et al., 2024). In pediatric classical Hodgkin lymphoma (cHL), plasma EV-enriched miR-122-5p at diagnosis was associated with subsequent relapse and inferior 5-year event-free survival. The association remained significant after adjustment for established clinical risk factors, providing preliminary evidence that miR-122-5p may contribute prognostic information. Nevertheless, its diagnostic performance was limited, and the prognostic threshold was developed and tested in the same cohort. Substantial outlier exclusion and the absence of independent or prospective assay validation further restrict the reproducibility and generalizability of the reported estimates. Moreover, miR-122-5p was largely absent from cHL tissues and cell lines, indicating that it is probably a systemic response marker rather than a tumor-derived biomarker. Its proposed involvement in SOCS1–JAK/STAT signaling was based mainly on computational predictions and correlative immune measurements and therefore requires direct functional confirmation (Salzmann et al., 2024). The lymphoma evidence combines mechanistic cell-culture observations with small clinical cohorts, but neither study established that the analyzed preparations consisted exclusively of endosome-derived exosomes. The cellular evidence for an immunosuppressive function is biologically plausible but requires pathway-specific blocking experiments and independent replication. Plasma sEV concentration, CD20, PD-L1 and miR-122-5p should therefore be described as experimental candidates rather than clinically validated biomarkers because standardized thresholds, analytical sensitivity and specificity, external reproducibility, and prospective validation remain unavailable.

### 5.2 Leukemia

EVs released by CLL cells following CD40/IL-4 stimulation carry a unique miRNA cargo and are selectively enriched in miR-363 compared with the parental tumor cells. These EVs are preferentially internalized by autologous CD4^+^ T cells, in which miR-363 specifically targets the immunomodulatory receptor CD69, leading to its downregulation. Functionally, CLL-derived EVs reprogram T cells toward phenotypes characterized by enhanced motility, greater immunological synapse formation with CLL tumor cells, and higher proliferative capacity. Strikingly, knockdown of miR-363 in CLL cells abolished the ability of their EVs to reprogram CD4^+^ T-cell signaling, highlighting the key importance of this miRNA. These experiments used small numbers of patient samples and an artificial *ex vivo* stimulation system that models, but does not reproduce fully, the lymph-node microenvironment. Differential ultracentrifugation also produced a heterogeneous sEV-enriched fraction rather than a purified exosome population, and no animal or patient-level evidence demonstrated that this mechanism influences CLL progression *in vivo* (Smallwood et al., 2016). This study provides stronger mechanistic evidence than the lymphoma biomarker studies because it combines target validation with loss-of-function experiments in primary autologous cells. Its biological relevance nevertheless requires replication in larger cohorts and confirmation in animal models or patient tissues. miR-363 was investigated as a mediator of intercellular communication, not as a clinically validated biomarker.

### 5.3 Comparative analysis across hematological malignancies

Current evidence indicates malignancy-specific rather than shared EV-mediated regulation of CD4^+^ T cells. Lymphoma studies primarily suggest checkpoint-associated T-cell apoptosis and exploratory circulating biomarkers, whereas CLL-derived EVs produced miRNA-dependent changes in CD4^+^ T-cell activation, motility, proliferation and tumor-cell interactions (Akil et al., 2024; Salzmann et al., 2024; Smallwood et al., 2016). The CLL mechanism has stronger causal support because it was examined using primary autologous cells and loss-of-function experiments, while the lymphoma findings have greater clinical representation but weaker mechanistic attribution. No comparable MM evidence or independently reproduced common pathway was identified. Overall, small cohorts, predominantly *ex vivo* experiments and heterogeneous EV preparations prevent these findings from defining a general mechanism across hematological malignancies.

## 6 CD8^+^ T Cells

Tumor-derived EVs (TDEs) directly inhibit CD8^+^ T cells through surface-bound molecules (PD-L1, FasL, TRAIL, CD39/CD73, TβRII) and cargo (protein, miRNA, circRNA) that impair proliferation, activation, and cytotoxicity and promote exhaustion. Indirectly, TDEs can also drive CD8^+^ T cell dysfunction by reprogramming the TME, including by impairing dendritic cell function, polarizing M2 macrophages, expanding MDSC/Tregs, and inducing regulatory B cells. CD8^+^ T cell-derived EVs have both protumor and antitumor functions: some promote tumor immune evasion through effects on antigen presentation or cell invasion, while others have antitumor effects through delivery of cytotoxic miRNAs, stromal cell killing, or internalization of PD-L1 (Huang et al., 2024; Gholami, 2024). In this section, we discuss the interactions between CD8^+^ T cells and tumor-derived EVs in hematological malignancies (Figure 4).

**FIGURE 4:**
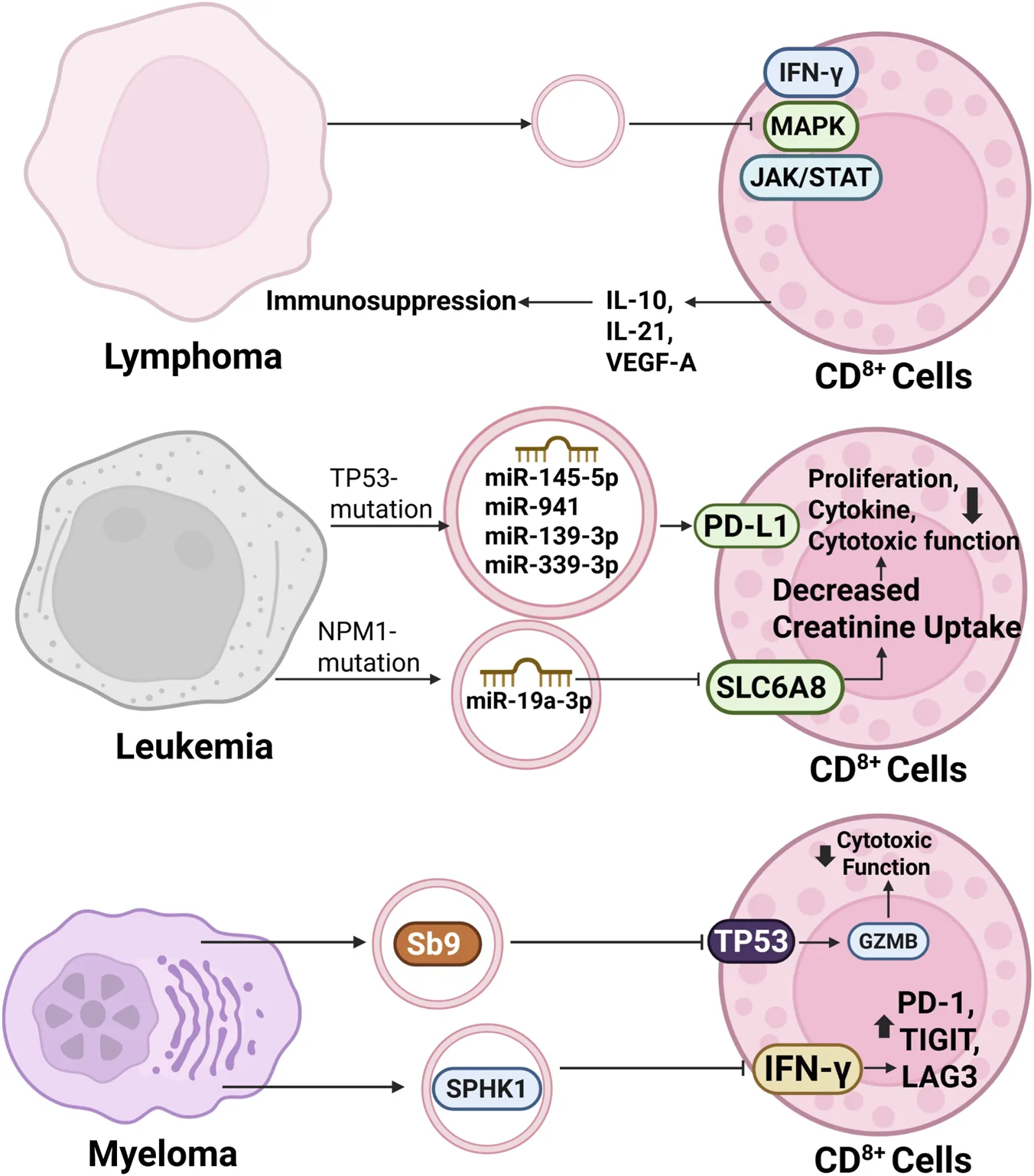
Tumor-derived EVs mediate CD8^+^ T-cell dysfunction in hematological malignancies. In lymphoma, EVs suppress CD8^+^ T-cell activation and cytotoxicity by inducing exhaustion and regulatory reprogramming through cytokines and inhibition of MAPK/JAK/STAT signaling. In leukemia, EVs from TP53- or NPM1-mutant cells deliver immunosuppressive miRNAs that enhance PD-L1 signaling or disrupt creatine metabolism via SLC6A8 repression, leading to impaired proliferation, cytokine production, and cytotoxic function. In multiple myeloma, EV-associated factors such as SPHK1/S1P signaling and Sb9 promote CD8^+^ T-cell exhaustion, TP53 destabilization, and reduced GZMB expression, collectively driving immune suppression and tumor immune escape.

### 6.1 Lymphoma

EVs from canine DLBCL inhibited CD8^+^ T-cell cytotoxicity via apoptosis, functional exhaustion, and regulatory reprogramming. EVs from the CLBL-1 cell line or plasma of dogs with DLBCL decreased T-cell viability and IFN-γ production, as well as increased expression of inhibitory checkpoints (CTLA-4, PD-1, PD-L1), and regulatory markers (FoxP3, TGF-β). In turn, this led to the expansion of CD8^+^ T cells with regulatory phenotypes that secreted IL-10, IL-21, and VEGF-A, fostering an immunosuppressive and pro-angiogenic milieu. Transcriptomic profiling of CD8^+^ T-cells exposed to EVs confirmed downregulation of MAPK and JAK-STAT signaling and cell cycle-associated genes, indicating inhibited proliferation and activation (Weng et al., 2023). The use of both cell-line- and plasma-derived EVs strengthens the biological interpretation. Nevertheless, the clinical component included only six dogs, whereas several *in vitro* assays used five to six samples and the exploratory cytokine analysis used only two replicates. Moreover, increased FoxP3, PD-1 and PD-L1 expression does not by itself establish conversion into stable regulatory CD8^+^ T cells. Direct suppressive assays and pathway-specific rescue experiments were not performed. The findings relevance to human DLBCL remains uncertain. The use of a polymer-based precipitation reagent for cell-culture EVs also raises concerns regarding co-isolated proteins and other non-vesicular components. Thus, the results require replication using human samples, more selective EV separation and functional validation of the proposed regulatory phenotype.

### 6.2 Leukemia

Among EV-enriched preparations from four acute leukemia cell lines, the Kasumi-1 preparation produced the largest reduction in the proportion of CD8^+^ T cells and increased Treg abundance and PD-1 expression in pooled healthy-donor PBMCs. This comparative design suggests that EV-mediated immune regulation varies among leukemia models. However, the cell lines differed in lineage, genetic background and TP53 status; therefore, the stronger effect of Kasumi-1 EVs cannot be attributed specifically to TP53 mutation. Moreover, the study measured immune-cell proportions and PD-1 expression but did not assess CD8^+^ T-cell proliferation, cytokine production, apoptosis or leukemia-cell killing. Sequencing identified differential miRNAs, including miR-145-5p, miR-941, miR-139-3p and miR-339-3p, while enrichment analyses implicated endocytosis, TGF-β signaling and extracellular-matrix pathways. These results were predictive rather than mechanistic: the candidate miRNAs and their targets were not validated experimentally, and neither clathrin-dependent uptake nor EV-associated PD-L1 enrichment was directly demonstrated. Polymer precipitation also produced an EV-enriched fraction that may have contained soluble proteins and other non-vesicular material. In the absence of patient samples, animal models, pathway-specific inhibition or independent replication, the findings provide preliminary cell-culture evidence rather than confirmation of an EV-mediated immune-evasion mechanism in TP53-mutant acute leukemia (Du et al., 2025). In NPM1-mutated AML, leukemic cells secrete sEVs enriched with miR-19a-3p through the NPM1mA/CTCF/PABPC1 axis. These vesicles are taken up by CD8^+^ T cells, where miR-19a-3p directly targets and represses SLC6A8 (creatine transporter), leading to decreased creatine uptake and ATP production. The resulting metabolic disruption hinders CD8^+^ T cell proliferation, cytokine production, and cytotoxic function, facilitating immune escape. Functionally, inhibiting sEV release or silencing miR-19a-3p restores CD8^+^ T cell function and prolongs survival in mice, suggesting sEV-associated miR-19a-3p as a driver of immune escape and potential therapeutic target in NPM1-mutated AML (Peng et al., 2021). Thus, both leukemia studies support a potential immunosuppressive effect of tumor-derived sEVs on CD8^+^ T cells, but the strength of evidence differs substantially. The first study was exploratory and relied on pooled healthy-donor PBMCs, heterogeneous leukemia cell lines and computational pathway predictions. In contrast, the latter combined primary AML samples, target validation, loss-of-function and rescue experiments, and a humanized mouse model, providing stronger evidence for the miR-19a-3p–SLC6A8 pathway. Nevertheless, neither study established exclusive exosomal origin, and both require independent confirmation in clinically characterized patient cohorts.

### 6.3 Multiple myeloma

EVs isolated from patients with multiple myeloma (MM) and from MM cell lines increased PD-1, TIGIT and LAG3 expression while reducing IFN-γ production in activated CD8^+^ T cells. EV-associated SPHK1 was implicated because pharmacological inhibition of SPHK1/S1P signaling and CRISPR-mediated SPHK1 knockout in donor MM cells attenuated checkpoint-marker induction. The combination of patient-derived EVs, pharmacological inhibition and genetic perturbation provides relatively strong mechanistic support. However, EVs were isolated by polymer precipitation, which can co-precipitate lipoproteins and soluble proteins, and recipient PBMCs were obtained from healthy donors rather than patients with MM. Moreover, increased checkpoint expression was used as a proxy for exhaustion without comprehensive assessment of persistent dysfunction, tumor-cell killing or reversibility. The results therefore support EV-mediated checkpoint regulation but do not fully establish terminal CD8^+^ T-cell exhaustion (Hagiwara et al., 2025). A separate study proposed a microbiota–lipid–EV pathway in which reduced Lachnospiraceae and increased phosphatidylcholine promote the release of SerpinB9-containing sEVs from MM cells. Transferred SerpinB9 reduced TP53 UFMylation and stability in CD8^+^ T cells, thereby decreasing granzyme B expression and cytotoxicity. The causal chain was supported by microbiome and metabolomic profiling, molecular perturbation experiments and *in vivo* models. However, the initial microbiome comparison included only 20 patients and 38 controls, and metagenomic sequencing was restricted to five participants per group. Treatment, diet, antibiotic exposure and other factors may also confound microbiome differences. In addition, membrane-affinity isolation and PKH67 labeling do not establish that the functional cargo was transferred exclusively through exosomes. The proposed pathway is therefore mechanistically compelling but requires independent replication in larger, clinically characterized cohorts (Yan et al., 2025).

### 6.4 Comparative analysis across hematological malignancies

Compared with the heterogeneous NK-cell findings, the CD8^+^ T-cell evidence shows greater directional consistency: EV preparations from lymphoma, leukemia and multiple myeloma generally reduced antitumor T-cell activity. Shared outcomes included decreased viability, proliferation, cytokine production or cytotoxicity, together with increased expression of inhibitory checkpoints. However, checkpoint expression alone does not establish stable or terminal exhaustion, and only some studies directly assessed tumor-cell killing or performed mechanistic rescue experiments (Weng et al., 2023; Du et al., 2025; Peng et al., 2021; Hagiwara et al., 2025; Yan et al., 2025).

The apparent mechanisms differed across malignancies. Lymphoma-derived EVs produced broad apoptotic, checkpoint and regulatory changes in CD8^+^ T cells, but the evidence was limited to a small canine DLBCL model without pathway-specific rescue (Weng et al., 2023). Leukemia studies ranged from exploratory changes in CD8^+^ T-cell abundance and PD-1 expression to a more clearly defined metabolic mechanism in NPM1-mutated AML, where EV-associated miR-19a-3p impaired creatine uptake and cellular energy production (Du et al., 2025; Peng et al., 2021). In MM, the reported effects involved both induction of multiple inhibitory receptors and suppression of granzyme-dependent cytotoxicity, linking EV activity to checkpoint regulation and loss of effector function (Hagiwara et al., 2025; Yan et al., 2025). Thus, the common biological outcome was CD8^+^ T-cell dysfunction, but no single EV cargo or signaling pathway was shared across all three malignancy groups.

Evidence is strongest for the NPM1-mutated AML and MM mechanisms because they incorporated primary patient material, genetic or pharmacological perturbation, functional rescue and animal experiments. The lymphoma study and the comparative acute-leukemia cell-line study remain more exploratory. Nevertheless, all proposed mechanisms require independent clinical validation. Differences in species, donor and recipient cells, EV isolation, exposure conditions and definitions of exhaustion prevent quantitative comparison across diseases. The current evidence therefore supports convergent CD8^+^ T-cell suppression through malignancy- and cargo-specific pathways rather than a universal EV-mediated mechanism.

## 7 Dendritic cells

Tumor-derived EVs (TEVs) impair dendritic cell (DC) activation, function, antigen presentation, and promote tolerogenic or myeloid-suppressive phenotypes, via molecules such as PD-L1, PGE_2_, HLA-G, and Galectin-9. DC-EVs, on the other hand, are highly immunostimulatory, as they contain MHC I/II–peptide complexes, co-stimulatory (CD80, CD86) and NK-activating ligands, which further activate T cells and NK cells. DC-EVs can directly activate tumor-specific cytotoxic T or NK cell responses, or can “cross-dress” by transferring their antigen cargo to other antigen-presenting cells, boosting immune activation (Schioppa et al., 2024). Figure 5 shows the molecular interactions between DCs and tumor derived EVs in hematological malignancies.

**FIGURE 5:**
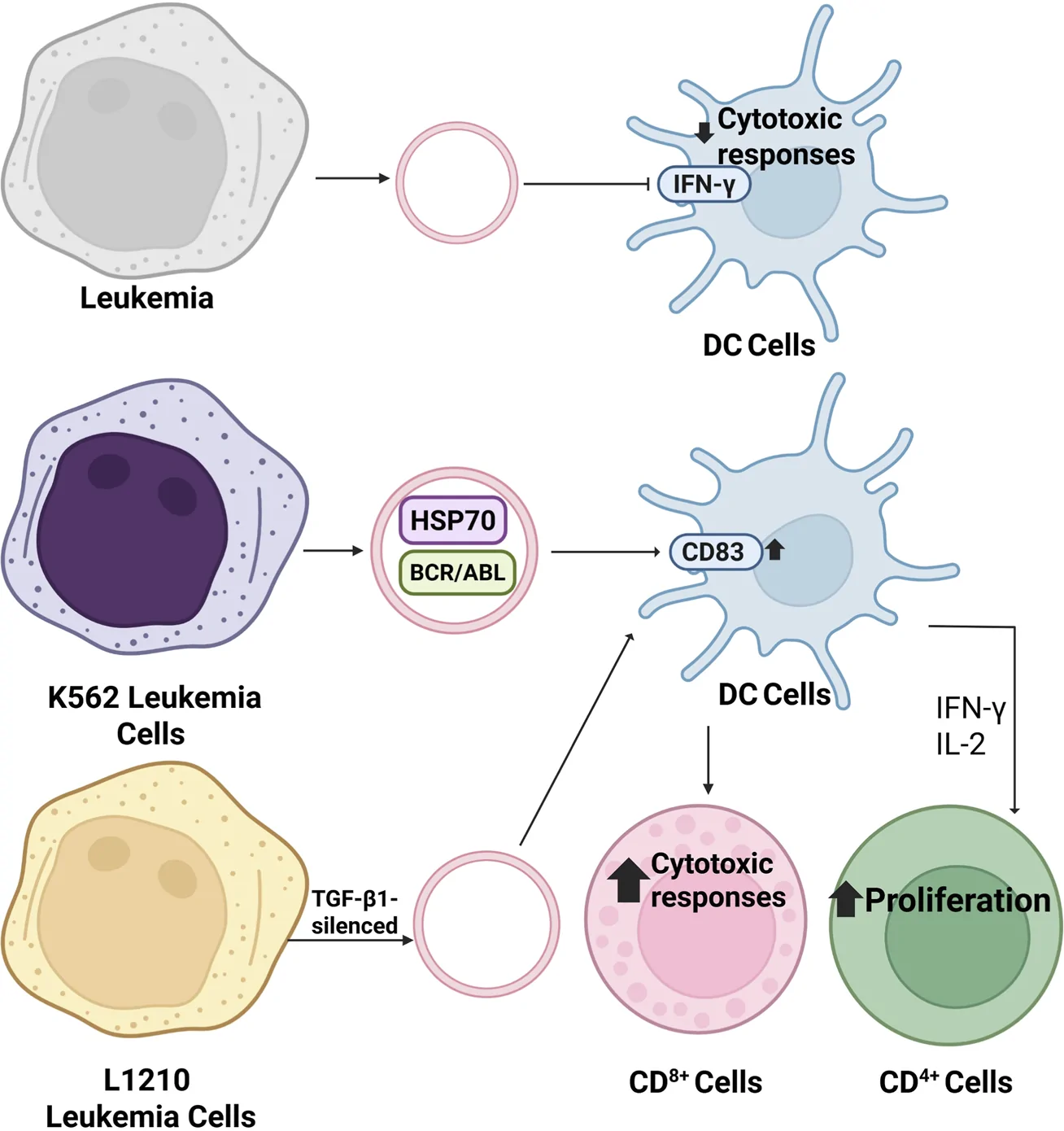
Tumor-derived EVs differentially regulate DC function in leukemia. Leukemia patient–derived EVs promote tolerogenic DCs and suppress IFN-γ–dependent cytotoxic responses, whereas leukemia cell–or engineered EVs carrying immunogenic cargo (e.g., HSP70, BCR-ABL, or TGF-β1-silenced EVs) drive DC maturation and enhance CD8^+^ and CD4^+^ T-cell activation and antileukemic immunity.

### 7.1 Leukemia

Serum EV-enriched preparations from patients with AML reduced DC-mediated cytotoxicity against K562 cells and, in selected experiments, decreased lymphocyte IFN-γ production. Conversely, K562 cell-derived EVs and cell lysates enhanced CD83 expression and cytotoxicity when applied to immature DCs. However, the patient cohort included only eight AML cases, and the IFN-γ analysis was restricted to two patients selected for their pronounced functional responses. Serum EVs were precipitated using ExoQuick and could have included vesicles from tumor, immune, endothelial and platelet sources, as well as non-vesicular proteins. Accordingly, these preparations cannot be classified as AML blast-derived exosomes. Moreover, increased CD86 expression after exposure to patient EVs was inconsistent with a conventional tolerogenic phenotype. The results therefore demonstrate suppression of DC-mediated cytotoxicity *in vitro* but do not establish stable tolerogenic DC reprogramming (Benites et al., 2019). The opposing effects of patient-serum and K562-derived EVs may reflect differences in vesicle source, cargo and experimental context. Circulating EVs represent a heterogeneous systemic mixture potentially enriched in immunosuppressive factors, whereas K562 preparations may carry abundant leukemia-associated antigens and HSP70. The use of K562 cells as both the EV source and cytotoxicity target may also have favored antigen-matched responses. Furthermore, K562 is a chronic myeloid leukemia-derived cell line and does not fully represent AML biology. These methodological differences prevent a direct conclusion that patient-derived and cultured leukemia-cell EVs have intrinsically opposite functions. EVs from leukemia-derived dendritic cells (DCleu) and mixed lymphocyte cultures (MLCs) were shown to actively boost antileukemic immune responses in AML. Cultures of both AML patient cells and healthy donor cells, with or without Kit M stimulation, found that EVs produced during Kit M treatment were more effective at T-cell activation and increased blast lysis. Profiling of the different EV populations showed that after DC culture, there was an increase in CD8^+^, CD2^+^, and thrombocyte-associated (CD41b^+^, CD62P^+^) EVs, as well as an increase in CD133^+^ progenitor/blast-related EVs in AML samples. However, the isolated EVs were not transferred into independent recipient cultures to determine whether they caused T-cell activation or blast killing. Surface markers detected by bead-based flow cytometry also cannot definitively identify the cellular origin of individual EVs. Thus, the study supports EVs as exploratory indicators of *ex vivo* immune activation, but not as demonstrated mediators of the antileukemic effect of Kit M (Li et al., 2025). Leukemia-cell sEV-enriched preparations were also evaluated as antigen sources for DC vaccination. K562-derived preparations contained BCR–ABL and HSP70 and were internalized by DCs, whereas functional vaccination experiments used L1210-derived preparations in a syngeneic mouse model. DCs pulsed with L1210 sEVs induced stronger leukemia-specific cytotoxicity, delayed tumor growth and prolonged survival compared with sEVs or unpulsed DCs alone. Protection against L1210 rechallenge, but not against unrelated P388 cells, supported antigen specificity. The use of differential ultracentrifugation, sucrose-density purification, functional assays and preventive and therapeutic animal models strengthens the evidence. Nevertheless, K562 is derived from chronic myeloid leukemia and L1210 is a murine lymphocytic leukemia model; therefore, these findings cannot be interpreted as direct evidence for an AML vaccine. The animal groups were also small, and no human patient-derived DC vaccination model was examined (Yao et al., 2014). The contribution of immunosuppressive cargo was investigated by silencing TGF-β1 in L1210 donor cells before collecting the sEV-enriched fraction. Compared with unmodified preparations, TGF-β1-depleted sEVs promoted DC maturation, increased IL-12p70 and TNF-α production, and enhanced antigen-specific CD4^+^ and CD8^+^ T-cell responses. In preventive and therapeutic mouse models, DC vaccination using these modified sEVs reduced tumor growth, prolonged survival, increased circulating CD4^+^ and CD8^+^ T cells and decreased Tregs. The inclusion of control-shRNA preparations, direct measurement of vesicular TGF-β1 and *in vivo* validation supports a causal contribution of TGF-β1. However, TGF-β1 was reduced rather than completely eliminated, and the mechanism through which vesicular TGF-β1 regulated DC-derived TGF-β1 was not established. The findings remain limited to an engineered murine system and require confirmation using human leukemia cells and patient-derived DCs (Huang et al., 2017a). Collectively, these studies show that leukemia-associated EVs can either suppress or enhance DC-dependent immunity. The most plausible explanation is that the net effect depends on EV origin, molecular cargo, DC maturation state and experimental use. Heterogeneous circulating EVs from patients suppressed DC-mediated cytotoxicity, whereas cell-line sEVs carrying leukemia-associated antigens stimulated DC vaccination responses. The enhanced activity obtained after TGF-β1 depletion further indicates that immunogenic and immunosuppressive cargos coexist within leukemia-cell EV preparations. Evidence is strongest for the TGF-β1 mechanism because it was examined through genetic modification and controlled animal experiments. Nevertheless, none of these approaches has undergone clinical validation, and differences in EV isolation, leukemia subtype and recipient-cell systems limit cross-study reproducibility.

### 7.2 Comparative analysis across hematological malignancies

The leukemia studies reveal a context-dependent relationship between EVs and DC-mediated immunity rather than a uniformly suppressive or stimulatory effect. Circulating EV-enriched preparations from patients impaired DC-mediated cytotoxicity, whereas leukemia-cell preparations used as antigen sources promoted DC maturation and antileukemic responses. These findings are not necessarily contradictory because the preparations differed in cellular origin, cargo composition, purity, recipient-cell state and experimental purpose. Patient-serum EVs represented heterogeneous systemic mixtures, while cell-line preparations were enriched for leukemia-associated antigens and stress proteins and were tested under vaccination-oriented conditions. Antigen matching may also have contributed to the stronger responses observed when K562 cells served as both the EV source and cytotoxicity target (Benites et al., 2019; Yao et al., 2014). The strength of evidence varied among the studies. Changes in EV phenotypes during Kit M–induced DC/DCleu generation correlated with T-cell activation and blast lysis, but EV transfer experiments were not performed; therefore, these vesicles are better interpreted as exploratory indicators of immune activation than as demonstrated mediators (Li et al., 2025). The vaccination studies provided stronger functional evidence because they assessed antigen-specific cytotoxicity, tumor growth, survival and rechallenge responses *in vivo*. In particular, the enhanced activity of DCs loaded with TGF-β1-depleted preparations supports coexistence of immunogenic and immunosuppressive cargo within leukemia-cell EV populations and provides causal evidence that TGF-β1 can restrict their immunotherapeutic activity (Yao et al., 2014; Huang et al., 2017a). Nevertheless, the strongest findings remain confined to engineered murine leukemia models, whereas the human evidence derives from small and heterogeneous cohorts. Differences in leukemia subtype, EV isolation, DC-generation protocols and functional endpoints further limit direct comparison. Collectively, the evidence supports two potential roles for leukemia-associated EVs: circulating EV mixtures may suppress DC-dependent immunity, while selected or modified leukemia-cell sEV preparations may serve as antigen sources for DC vaccination. Neither role has been clinically validated, and the preparations should generally be described as EV- or sEV-enriched rather than as purified exosomes.

## 8 Myeloid-derived suppressor cells

Researchers reviewed how tumor-derived extracellular vesicles (TD-EVs) foster an immunosuppressive tumor microenvironment by activating and expanding myeloid-derived suppressor cells (MDSCs). TD-EVs carry bioactive molecules such as PGE_2_, TGF-β, Hsp72, IL-10, and miRNAs (e.g., miR-21, miR-10a, miR-155) that enhance MDSC survival and suppressive function through STAT3, TLR2/MyD88, and NF-κB signaling. Tumor microenvironmental factors like hypoxia and acidity further promote TD-EVs release and cargo loading (Tian et al., 2019). Figure 6 shows the molecular interactions between MDSCs and tumor-derived EVs in hematological malignancies.

**FIGURE 6:**
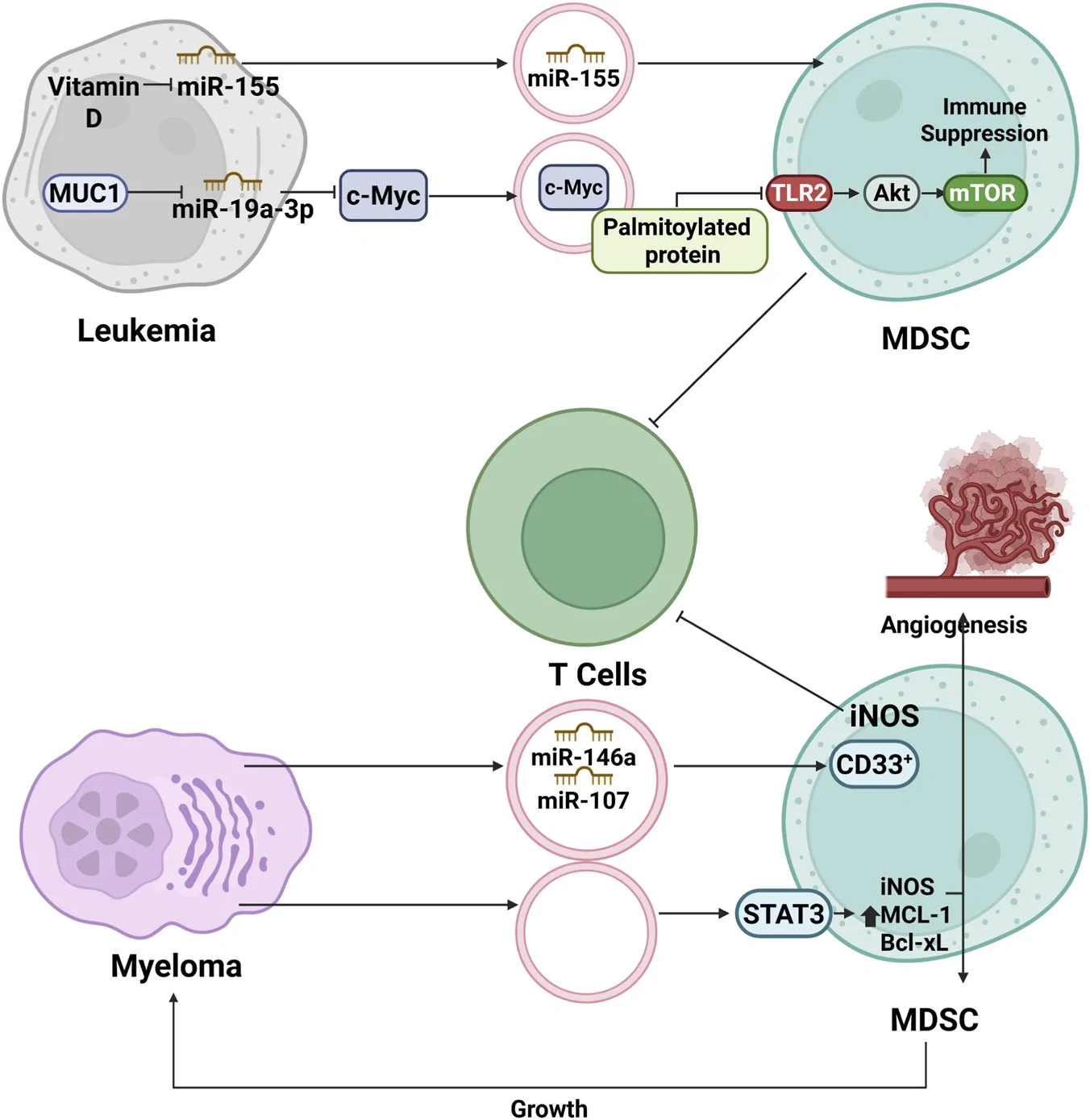
Tumor-derived EVs from leukemia and multiple myeloma promote expansion and immunosuppressive reprogramming of myeloid-derived suppressor cells via transfer of palmitoylated proteins and miRNAs, activating TLR2/Akt/mTOR and STAT pathways, suppressing T-cell responses, and supporting tumor growth and angiogenesis.

### 8.1 Leukemia

AML-cell sEV-enriched preparations induced the differentiation of healthy-donor monocytes into CD14^+^HLA-DR^low MDSC-like cells through surface palmitoylated proteins and TLR2/Akt/mTOR signaling. Proteinase K treatment, inhibition of donor-cell palmitoylation, TLR2 blockade and rapamycin treatment reduced MDSC differentiation and T-cell suppression, providing convergent evidence for a surface protein-dependent mechanism. Density-gradient fractionation and experiments with primary AML samples strengthened the attribution of this activity to EV-associated material. Nevertheless, most functional experiments used AML cell lines and small numbers of healthy donors, while primary patient-derived preparations were evaluated only in limited assays. Moreover, 2-bromopalmitate and rapamycin are not pathway-exclusive, and the specific palmitoylated TLR2 ligand was not identified. The findings therefore support a mechanistic role for EV-surface palmitoylated proteins *in vitro* but require independent replication and *in vivo* validation (Tohumeken et al., 2020). A separate AML study connected EV-mediated MDSC expansion to the MUC1/miR-34a/c-Myc pathway. MUC1 expression in AML cells was associated with reduced miR-34a and increased c-Myc in EV-enriched preparations, followed by increased cyclin D2 and cyclin E1 expression in recipient myeloid cells. MUC1 silencing reversed these effects and reduced the generation of suppressive myeloid cells, supporting a causal regulatory sequence. Patient samples and a murine AML model provided disease-related context, but the EV-specific mechanism was established predominantly in cell-line experiments. The small patient groups, limited vesicle characterization and use of a spin-column isolation procedure also make it difficult to exclude contributions from non-vesicular proteins or mixed EV populations. Direct *in vivo* manipulation of EV-associated MUC1, miR-34a or c-Myc was not performed (Pyzer et al., 2017). These AML studies converge on the conclusion that EV-associated material can reprogram myeloid progenitors, but they identify different initiating mechanisms. First study implicates palmitoylated surface proteins and TLR2, whereas the latter emphasizes MUC1-dependent regulation of miR-34a and c-Myc cargo. These mechanisms are not necessarily contradictory because surface receptor activation and transferred regulatory cargo could operate in parallel. However, differences in AML cell lines, recipient-cell populations, EV isolation procedures and MDSC definitions prevent determination of which pathway predominates in patients. In CLL, sEV-enriched preparations transferred miR-155 to healthy-donor monocytes and induced an HLA-DR^low, IDO-positive and glycolytically reprogrammed suppressive phenotype. The functional relevance of miR-155 was supported by antagomir experiments that prevented IDO induction and metabolic reprogramming. Pretreating CLL cells with the active vitamin D metabolite reduced NF-κB activity and cellular and EV-associated miR-155, thereby weakening MDSC induction without measurably changing particle release or uptake. These loss-of-function and donor-cell perturbation experiments provide reasonable mechanistic evidence, although the reported isolation procedure and EV characterization were insufficient to establish an exclusively endosomal vesicle population (Bruns et al., 2017). The clinical observations of this study should be interpreted more cautiously. Low circulating 25-hydroxyvitamin D was associated with increased CD14^+^HLA-DR^low cells in 39 untreated patients, but this observational relationship may be affected by disease severity, inflammation, nutritional status and other confounders. Moreover, the patient analysis measured circulating 25-hydroxyvitamin D, whereas the cell experiments used the active 1,25-dihydroxyvitamin D metabolite. The study therefore does not demonstrate that vitamin D supplementation suppresses MDSCs or improves clinical outcomes in CLL. The leukemia evidence consistently supports an immunosuppressive effect of EV-enriched preparations on myeloid cells, but the strongest support is mechanistic and preclinical. No study independently reproduced the complete proposed mechanism in a separate cohort or demonstrated that pathway inhibition improves leukemia control through an EV-dependent mechanism in patients. The absence of immunostimulatory findings in this subsection may also reflect the selection of studies focused specifically on MDSC induction rather than a universal property of leukemia-associated EVs.

### 8.2 Multiple myeloma

MM-cell EV-enriched preparations containing miR-106a-5p and miR-146a-5p promoted the differentiation of healthy-donor peripheral blood mononuclear cells into monocytic MDSC-like cells. Combined inhibition of both miRNAs reduced M-MDSC induction, whereas inhibition of either miRNA alone was insufficient, suggesting partly compensatory activity. CCL5 and MIF further enhanced the response, indicating that vesicular and soluble signals may cooperate. However, removing EVs from conditioned medium did not clearly eliminate M-MDSC induction, and direct co-culture produced stronger effects than isolated preparations. These findings weaken the interpretation that EV-associated miRNAs are independently sufficient or essential for the observed myeloid reprogramming. The clinical support for this miRNA mechanism was also limited. Although malignant plasma cells expressed the candidate miRNAs, their abundance was not consistently increased across patient groups, and circulating EV-associated miR-106a-5p and miR-146a-5p did not clearly distinguish patients from healthy controls. In addition, pathway enrichment analyses identified broad inflammatory programs but did not establish the direct molecular targets of either miRNA in M-MDSCs. The results therefore identify an experimental mechanism in selected MM cell lines rather than a validated patient-associated EV signature (Mizuhara et al., 2023). MM-cell sEV-enriched preparations also expanded MDSCs and enhanced their suppressive activity through STAT3 signaling. The preparations increased MDSC viability, CD11b expression and iNOS-dependent inhibition of T-cell proliferation. Repeated administration to naïve mice increased MDSC populations in the bone marrow and spleen, providing stronger physiological evidence than isolated cell-culture experiments. Protease treatment and reprecipitation controls reduced the likelihood that freely soluble proteins entirely explained the effects. Nevertheless, polymer precipitation does not separate sEVs from all proteins, lipoproteins or other vesicle populations, and the relatively high protein-normalized doses may not reproduce physiological exposure. The responsible STAT3-activating cargo also remained unidentified (Wang et al., 2016). Bone marrow stromal cell-derived EV-enriched preparations similarly increased MDSC survival and suppressive activity through STAT1 and STAT3 signaling, accompanied by increased Bcl-xL, Mcl-1 and nitric oxide production. Pharmacological inhibition reduced MDSC viability and T-cell suppression, while short-term administration in MM-bearing mice supported an effect within the tumor-associated bone marrow environment. However, the inhibitors also affected MDSCs in the absence of EV treatment, making it difficult to separate pathway dependence of basal MDSC survival from an EV-specific mechanism. The animal experiments assessed short-term immune changes rather than tumor growth or survival, and no human stromal-cell or patient-derived EV preparations were evaluated (Wang et al., 2015). Studies (Wang et al., 2016) and (Wang et al., 2015) both implicate STAT signaling but examine biologically distinct EV sources. Study (Wang et al., 2016) used MM-cell-derived preparations, whereas study (Wang et al., 2015) investigated bone marrow stromal cell-derived preparations. The latter should therefore not be described as tumor-derived EVs, although stromal EVs may contribute to a tumor-supportive microenvironment. This distinction is important because tumor and stromal EV populations may differ in cargo, abundance and therapeutic accessibility. MM studies consistently report MDSC-promoting activity, but the evidence varies considerably. Study (Mizuhara et al., 2023) provides miRNA perturbation data but lacks animal validation and shows limited agreement between cell-line findings and patient samples. Studies (Wang et al., 2016) and (Wang et al., 2015) add functional and *in vivo* evidence, with STAT3 emerging as a recurrent pathway, although both rely on polymer-based EV enrichment and predominantly murine systems. The convergence on STAT signaling may indicate a shared downstream response rather than a common EV cargo. None of the reported miRNAs, signaling components or EV populations has been prospectively validated as a clinical biomarker or therapeutic target in MM.

### 8.3 Comparative analysis across hematological malignancies

Across leukemia and multiple myeloma, the findings converge on a predominantly immunosuppressive effect: EV-enriched preparations promote the generation, survival or suppressive activity of MDSC-like populations, ultimately weakening T-cell responses. This directional consistency is stronger than that observed for several other immune-cell populations. Nevertheless, the absence of immunostimulatory findings may partly reflect the selection of studies specifically designed to investigate MDSC induction rather than a universal property of tumor-associated EVs (Tohumeken et al., 2020; Pyzer et al., 2017; Bruns et al., 2017; Mizuhara et al., 2023; Wang et al., 2016; Wang et al., 2015). The initiating mechanisms appear disease- and source-dependent. In AML, EV-associated material acted through either surface palmitoylated proteins engaging TLR2/Akt/mTOR signaling or MUC1-dependent regulation of miR-34a and c-Myc cargo. In CLL, transferred miR-155 promoted an IDO-positive, glycolytically reprogrammed suppressive phenotype through an NF-κB-associated pathway. MM studies instead implicated cooperative miRNA and soluble-factor signaling or activation of STAT pathways that enhanced MDSC viability and suppressive function. These mechanisms need not be mutually exclusive: receptor engagement, transferred regulatory cargo and soluble mediators may operate simultaneously or sequentially. The recurrence of STAT3 in MM may represent convergence on a common downstream MDSC-survival program rather than evidence for a shared vesicular cargo. The evidence also differs in strength. Leukemia studies generally provide more cargo- or pathway-specific perturbation, including proteinase K treatment, TLR2 blockade, MUC1 silencing and miR-155 antagomirs, but remain dominated by cell-line experiments and small donor or patient cohorts. MM studies provide greater *in vivo* support for MDSC expansion within bone marrow and splenic compartments, although their reliance on polymer-enriched preparations and predominantly murine systems weakens EV-specific and clinical attribution. Importantly, stromal-cell-derived EVs in MM should be distinguished from tumor-cell-derived EVs, even if both support the same suppressive microenvironment. The studies support functional convergence on MDSC-mediated immune suppression through heterogeneous upstream mechanisms. However, none has established which pathway predominates in patients, demonstrated improved clinical outcomes after EV-specific pathway inhibition, or prospectively validated the proposed cargos as biomarkers or therapeutic targets. Differences in EV source, separation method, recipient-cell definition and experimental exposure therefore remain major barriers to cross-study comparison and clinical translation.

## 9 Monocytes

EVs are key modulators of monocyte function. After uptake, EVs can reprogram monocytes to an immunosuppressive, pro-tumoral phenotype. EVs transfer oncogenic proteins, lipids, and RNAs, which activate signaling pathways such as TLR2/4, PI3K/Akt, and STAT3. This causes monocytes to secrete immunosuppressive factors such as IL-6, IL-10, and TNF-α, and express immune checkpoint molecules such as PD-L1 and CD14, and the scavenger receptor CD206. The net result is the differentiation of monocytes into tumor-associated macrophages or M-MDSCs. This enhances tumor immune evasion, angiogenesis, and progression. Therefore, EVs are key mediators of tumor–monocyte crosstalk (Almeida et al., 2024). Figure 7 shows the molecular interactions between monocytes and tumor-derived EVs in hematological malignancies.

**FIGURE 7:**
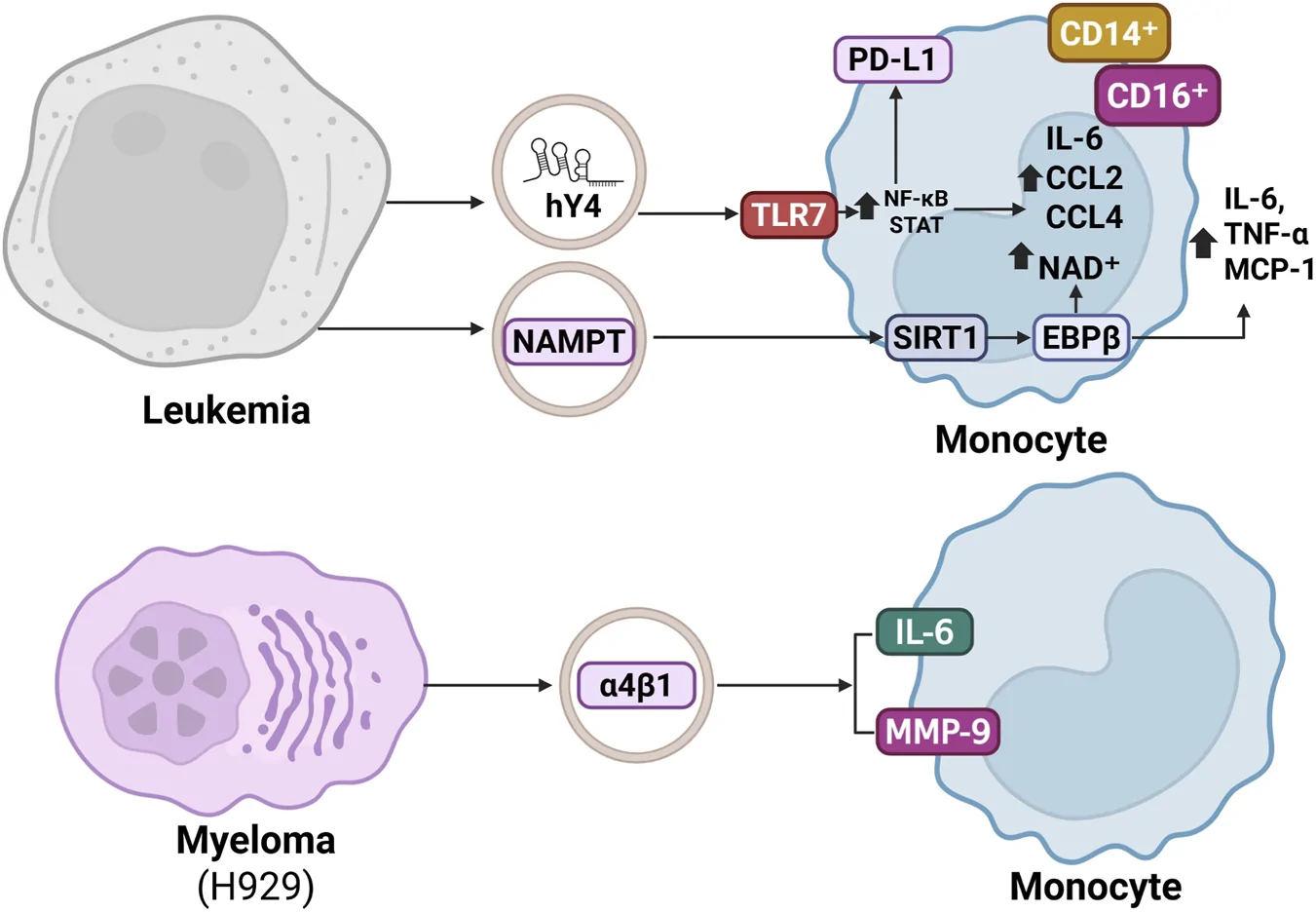
Leukemia- and myeloma-derived extracellular vesicles reprogram monocytes toward a proinflammatory and immunosuppressive phenotype by delivering ligands such as hY4, NAMPT, and α4β1, activating TLR7/NF-κB/STAT and SIRT1–EBPβ signaling, inducing PD-L1 expression, inflammatory cytokine production, and matrix remodeling factors.

### 9.1 Leukemia

CLL-cell EV-enriched preparations transferred the noncoding Y RNA hY4 to primary monocytes, inducing NF-κB- and STAT-associated inflammatory responses, including CCL2, CCL4 and IL-6 production and increased surface PD-L1. Synthetic hY4 reproduced several of these effects, while TLR7-deficient murine myeloid cells failed to respond, supporting endosomal TLR7 as a relevant sensor. Plasma EV preparations from patients also contained increased hY4, but the functional experiments predominantly used EVs from the MEC-1 cell line. Moreover, selective depletion of hY4 from donor-cell EVs was not performed. The results therefore show that hY4 is sufficient to activate TLR7 but do not establish that it is the only or principal mediator of the complete EV-induced monocyte response (Haderk et al., 2017). The study combined patient samples, primary monocytes, genetic TLR7 deficiency and an Eμ-TCL1 mouse model, providing evidence across several experimental systems. Nevertheless, circulating EV preparations were heterogeneous and could have originated from tumor, immune, endothelial or platelet populations. The *in vivo* experiments used chloroquine to inhibit endosomal TLR signaling, but chloroquine has broad cellular effects and can act directly on malignant cells. Furthermore, RNY4 is absent in mice, so the reduced leukemia burden after chloroquine treatment cannot validate the human hY4 mechanism *in vivo*. Increased PD-L1 suggests immunoregulatory potential, but direct suppression of T-cell function by the EV-treated monocytes was not examined. ER stress provided a second mechanism through which CLL-cell EV-enriched preparations altered monocytes. Preparations from tunicamycin-treated MEC-1 and HG3 cells contained increased NAMPT and enhanced monocyte NAD^+^ production, SIRT1–C/EBPβ signaling, survival and acquisition of a CD16-positive phenotype. NAMPT knockdown in donor cells prevented the increases in monocyte NAD^+^, SIRT1, C/EBPβ, survival and CD16 expression, while SIRT1 inhibition produced similar effects. Exclusion of tunicamycin carryover by mass spectrometry and the use of NAMPT loss-of-function experiments strengthen the proposed metabolic mechanism. The clinical and immunological implications remain uncertain. Although ER-stress markers were examined in 26 patients and were associated with more aggressive CLL, patient-derived EVs were not used to validate the NAMPT mechanism. Most functional experiments consisted of three replicates involving cell-line EVs and healthy-donor monocytes, with no animal validation. In addition, CD14 and CD16 expression alone cannot reliably distinguish intermediate from non-classical monocytes; therefore, the findings support a shift toward a CD16-positive phenotype rather than definitive differentiation into a particular monocyte subset. Increased IL-6, TNF-α and MCP-1 demonstrates inflammatory activation but does not by itself establish immunosuppressive or tumor-promoting function (Ni et al., 2023). The hY4-TLR7 and NAMPT–NAD^+^–SIRT1 pathways are complementary rather than contradictory. Haderk et al. examined RNA-mediated innate sensing under standard CLL-cell conditions, whereas Ni et al. investigated metabolic cargo released during experimentally induced ER stress. Both studies reported inflammatory cytokine production, while only the hY4 study demonstrated PD-L1 upregulation. This coexistence of inflammation and checkpoint expression illustrates how tumor-associated EVs may generate an inflammatory yet potentially immunosuppressive monocyte state. However, functional T-cell suppression and subsequent macrophage differentiation were not directly evaluated in either study. Evidence for EV-mediated monocyte reprogramming in CLL is supported by pathway-interference experiments but remains predominantly preclinical. Haderk et al. (Haderk et al., 2017) provided broader evidence through patient EV profiling, primary-cell experiments and genetic TLR7 studies, but the specific contribution of hY4 was not validated through selective depletion from EVs or in an hY4-compatible animal model. Ni et al. (Ni et al., 2023) provided a stronger donor-cell loss-of-function analysis for NAMPT, but the mechanism was restricted to an artificial ER-stress model and lacked patient-EV and *in vivo* confirmation. Neither pathway has been independently reproduced, clinically validated or shown to predict patient outcomes.

### 9.2 Multiple myeloma

Monocytes rapidly and efficiently internalize EVs released by MM cells via energy-dependent, receptor-mediated uptake, in a cell line-dependent manner. H929-derived EVs showed the greatest uptake and induced the strongest IL-6 and MMP-9 responses, whereas U266 and MM.1S EVs had weaker or negligible effects. Reduced uptake at 4 °C supported an energy-dependent process but did not establish receptor-mediated endocytosis. H929 EV–exposed THP-1 conditioned medium also enhanced H929-cell proliferation and migration; however, the roles of IL-6 and MMP-9 were not confirmed by neutralization, and residual EVs may have directly affected the myeloma cells. Proteomic analysis identified H929 EV enrichment in spliceosome-associated proteins, exocytic regulators, ITGA4, and ITGB1. Although rigorous EV purification and characterization strengthened the study, these associations remained correlative because no cargo-depletion, uptake-blocking, or rescue experiments were performed. Overall, Sheridan et al. provide technically strong evidence of cell line–dependent MM EV uptake and monocyte activation, but reliance on 3 MM cell lines and THP-1 cells limits generalizability. Thus, IL-6, MMP-9, α4β1 integrin, and spliceosome-associated proteins remain experimental candidates rather than validated mediators or biomarkers (Sheridan et al., 2024).

### 9.3 Comparative analysis across hematological malignancies

Across CLL and MM, EV-enriched preparations were readily internalized by monocytes and induced inflammatory or phenotypic reprogramming. Increased IL-6 production was a shared outcome, accompanied by chemokine and checkpoint responses in CLL and MMP-9 production in MM. However, inflammatory cytokine release should not be considered equivalent to immunosuppressive differentiation. Neither disease group provided direct evidence that EV-treated monocytes developed a stable tumor-associated macrophage or M-MDSC phenotype, suppressed T-cell function, or promoted disease progression in patients (Haderk et al., 2017; Ni et al., 2023; Sheridan et al., 2024). The CLL studies provide greater mechanistic depth and identify two complementary forms of EV-mediated signaling. The hY4–TLR7 pathway represents RNA-mediated innate sensing that activates NF-κB/STAT-associated inflammation and increases PD-L1, whereas the NAMPT–NAD^+^–SIRT1–C/EBPβ pathway links donor-cell ER stress to metabolic adaptation, monocyte survival and acquisition of a CD16-positive phenotype. These mechanisms are not contradictory because they were examined under different donor-cell conditions and may coexist within the CLL microenvironment. Nevertheless, the hY4 study did not selectively deplete hY4 from EVs, and its mouse experiments could not directly reproduce the human RNA mechanism. Conversely, NAMPT loss-of-function provided stronger cargo-specific evidence, but the pathway was demonstrated mainly in an experimentally induced ER-stress model without patient-derived EV or animal validation (Haderk et al., 2017; Ni et al., 2023). The MM evidence demonstrates substantial donor-cell heterogeneity: H929-derived preparations were taken up more efficiently and produced stronger monocyte responses than preparations from U266 or MM.1S cells. This variation suggests that EV abundance alone may not determine biological activity and that cargo composition or uptake efficiency is also important. Although EV purification and characterization were comparatively rigorous, the proposed roles of IL-6, MMP-9, integrins and spliceosome-associated proteins remain unconfirmed because neutralization, cargo-depletion and rescue experiments were not performed (Sheridan et al., 2024). Overall, CLL evidence is stronger for pathway-specific monocyte reprogramming, whereas MM evidence more clearly illustrates cell line-dependent variation in EV uptake and activity. Across both malignancies, the principal unresolved issue is whether the observed inflammatory states become functionally immunosuppressive and tumor-supportive *in vivo*. Independent validation using primary patient-derived EVs, physiologically relevant doses relevant doses and direct immune-suppression assays is therefore required before these cargos or responses can be considered therapeutic targets or biomarkers.

## 10 B Cells

EVs can also affect the B-cell compartment to promote cancer progression. They regulate the expansion of the immunosuppressive regulatory B cells (Bregs) and the activity of antitumor B cells (Zhang et al., 2015). In certain situations, EVs can be antigenic decoys. For instance, PDAC- or B-cell lymphoma-derived EVs presenting tumor antigens or CD20 can bind antibodies, forming immune complexes that shield tumor cells from complement- or rituximab-mediated cytotoxicity. On the other hand, B cell-derived EVs can also attenuate antitumor immune responses by hydrolyzing extracellular ATP into adenosine through CD39/CD73 and restraining post-chemotherapy T-cell reactivity (Kugeratski and Kalluri, 2021; Kato et al., 2020). Figure 8 shows the molecular interactions between B cells and tumor-derived EVs in hematological malignancies.

**FIGURE 8:**
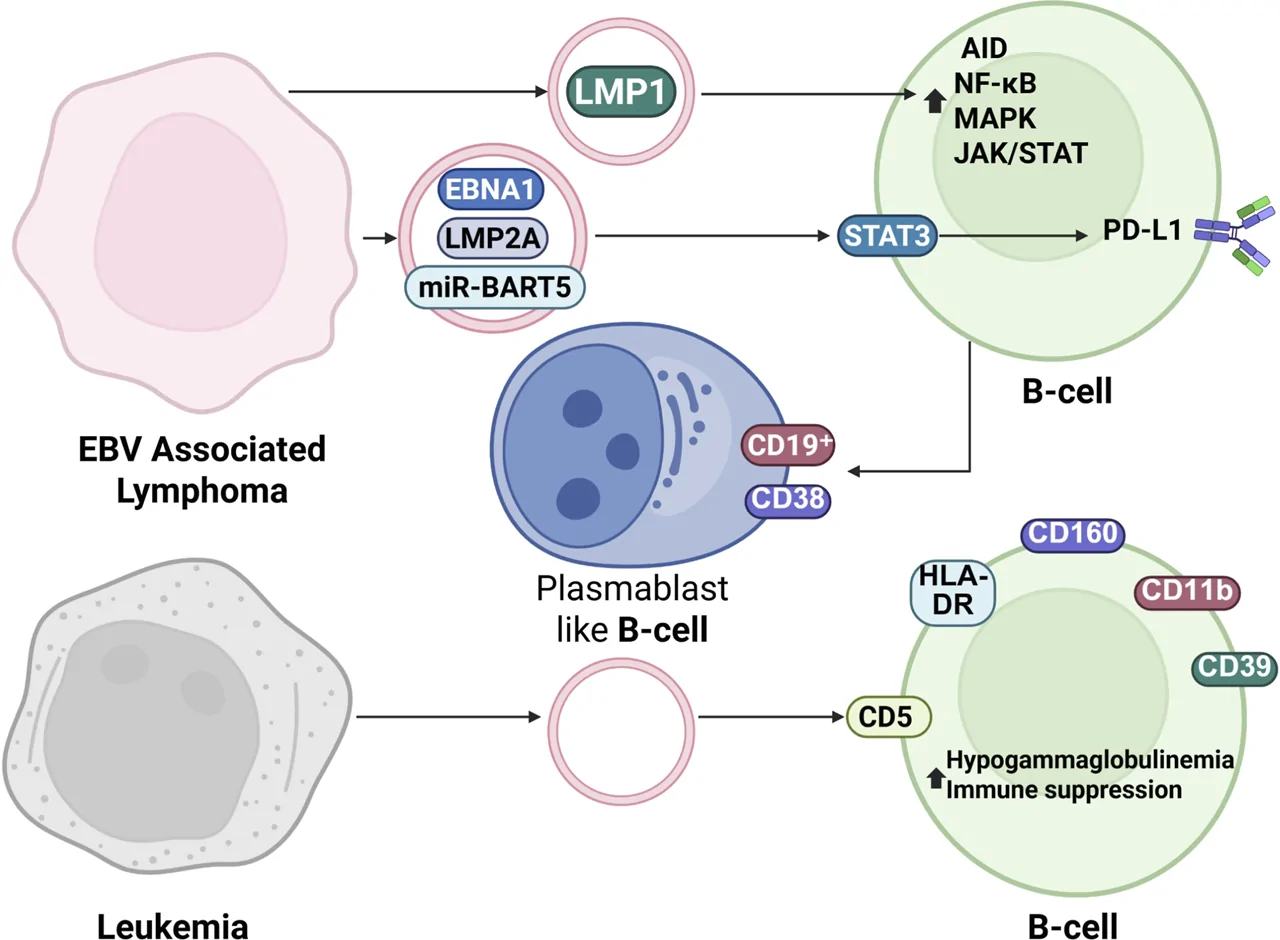
Tumor-derived extracellular vesicles modulate B-cell function in hematological malignancies by delivering viral and tumor-associated cargos that activate NF-κB/MAPK/JAK/STAT signaling, induce PD-L1 and IL-10 expression, drive plasmablast-like or regulatory B-cell phenotypes, and promote immune suppression and hypogammaglobulinemia.

### 10.1 Lymphoma

Mantle cell lymphoma (MCL) -cell sEV-enriched preparations were preferentially internalized by malignant and non-malignant B cells, whereas uptake by T cells, NK cells and stromal cells was limited. Experiments involving two MCL cell lines, primary MCL-cell cultures and pathway perturbation supported cholesterol- and lipid-raft-dependent uptake, while monocytes appeared to internalize the preparations through phagocytosis. However, primary-cell experiments involved samples from only three patients, and serum analyses included three additional cases. Lipophilic dyes and pharmacological uptake inhibitors may produce nonspecific effects, while serum precipitation cannot exclude platelet-, immune-cell- or endothelial-derived EVs and non-vesicular material. Thus, Hazan-Halevy et al. (2015) provide reasonable evidence for preferential B-cell uptake *in vitro*, but the responsible targeting ligand remains unidentified. The proposed use of MCL EVs to deliver cyclin D1-directed siRNA was not experimentally tested and should be presented as a potential application rather than an established therapeutic strategy (Hazan-Halevy et al., 2015). EBV-associated B-cell sEV-enriched preparations produced a different functional response. Preparations from DG75 Burkitt lymphoma sublines were internalized by primary human B cells and induced dose-dependent proliferation, AID expression and molecular indicators of IgG1 class-switch recombination. LMP1-containing preparations produced the most pronounced CD19^+^CD38^high^CD20^low^ plasmablast-like phenotype. Nevertheless, LMP1-negative preparations also stimulated proliferation, and the difference between LMP1-positive and control preparations showed only a statistical trend. Therefore, the response cannot be attributed exclusively to vesicular LMP1. Moreover, functional experiments involved four donors, class-switch transcripts were examined in only two donors, and immunoglobulin secretion was not demonstrated. Stronger proliferation in PBMC cultures than in purified B cells also indicates contributions from accessory cells and soluble mediators. These data support an immunostimulatory effect *in vitro*, but do not establish T-cell-independent humoral activation or a role in autoantibody production in patients (Gutzeit et al., 2014). EBV-positive B-cell sEV-enriched preparations were also reported to activate STAT3 and increase IL-10 and PD-L1 in Hut78 and H9 T-cell lymphoma lines. A STAT3 inhibitor reduced these responses, while tissue analysis showed increased IL-10 and PD-L1 in 32 cases of EBV-associated T/NK-cell lymphoproliferative disease compared with non-EBV control tissues. However, phosphorylated STAT3 was not itself significantly increased in the patient tissues, and the *in vitro* recipient cells were cutaneous T-cell lymphoma lines rather than patient-derived EBV-positive T/NK cells. The individual roles of EBNA1, LMP2A, miR-BART5 and miR-BART8-3p were not tested through cargo-specific perturbation. Chen et al. (2024) also did not directly examine cytotoxic T-cell or macrophage function, IFN-γ or TNF suppression, or disease progression *in vivo*. The findings therefore support an experimental STAT3–IL-10–PD-L1 response but not the broader conclusion that these EVs establish immune tolerance or maintain EBV-T/NK disease (Chen et al., 2024). Lymphoma-associated EV preparations exhibit both immunostimulatory and immunosuppressive activities, but these findings are not necessarily contradictory. Gutzeit et al. (Gutzeit et al., 2014) observed activation of normal B cells, whereas Chen et al. (Chen et al., 2024) examined checkpoint-related signaling in malignant T-cell lines. Differences in recipient-cell identity, EBV status, cargo composition, isolation method and EV dose could therefore explain the divergent outcomes. Evidence is strongest for preferential MCL-EV uptake by B cells because this was reproduced across cell lines and limited primary samples. Evidence for LMP1-dependent B-cell activation is moderate because appropriate control preparations were included, although LMP1 was not solely responsible. Evidence for STAT3-mediated immune escape is weaker because it relies on polymer-enriched preparations, pharmacological inhibition and indirect tissue associations. None of these findings has been clinically validated, and the proposed therapeutic-delivery application has not been evaluated in animals or patients.

### 10.2 Leukemia

In CLL, leukemia cell-derived sEVs significantly impact B-cell behavior and immune dysregulation. These sEVs are internalized by normal CD19^+^ B cells. The leukemic EVs induce the *de novo* expression of CD5 (a feature of the leukemic B cell phenotype) and the activation of several TNF signaling–related genes (TNF, TNFRSF18, LRP1B, RBP1). CLL sEVs also modulate surface immune checkpoint molecules by upregulating CD160, CD11b, CD39, and HLA-DR, while downregulating granzyme A and CD45RA. Functionally, this reprograms normal B cells toward an exhausted or tolerogenic phenotype that suppresses effective humoral immunity. CLL sEVs also induce apoptosis of autologous CLL cells and normal B cells, which could contribute to the hypogammaglobulinemia and immune suppression that is characteristic of CLL. However, the key B-cell transcriptional and protein analyses involved only three to four healthy donors, despite the larger overall patient cohort. Changes in surface-marker expression do not establish an exhausted or tolerogenic B-cell phenotype because antibody production, antigen presentation and B-cell receptor responses were not examined. Likewise, a contribution to hypogammaglobulinemia remains speculative because immunoglobulin levels were not linked prospectively to EV abundance or activity (Veletic et al., 2025). In a canine system, EV preparations from CLB70 leukemia and CLBL-1 lymphoma cell lines were rapidly taken up by activated T and B lymphocytes. Both preparations decreased the relative proportion of B cells without altering measured B-cell proliferation, while most memory-phenotype and cytokine effects occurred in T-cell subsets. In particular, reduced IL-17 was restricted to CD8^+^ T cells and should not be presented as evidence of direct B-cell suppression. Although researchers examined seven biological replicates using two EV doses, the PBMCs were strongly activated with concanavalin A and IL-2, which may not reproduce physiological immune conditions. Because absolute B-cell counts, apoptosis and B-cell effector functions were not assessed, a reduced percentage of B cells cannot be distinguished from reciprocal expansion or survival of T cells (Zmigrodzka et al., 2022). Evidence for leukemia-EV effects on B cells remains primarily *ex vivo*. Veletic et al. (Veletic et al., 2025) provide the stronger evidence because primary CLL preparations induced reproducible molecular and apoptotic responses, although the functional sample sizes were small and the proposed relationship with hypogammaglobulinemia was not tested. The canine study provides comparative evidence but has limited B-cell-specific mechanistic value and cannot be directly extrapolated to human leukemia (Zmigrodzka et al., 2022). Neither study demonstrates stable B-cell exhaustion, impaired antibody production or an EV-dependent clinical phenotype. No candidate EV biomarker was evaluated for sensitivity, specificity, assay reproducibility or prospective performance.

### 10.3 Comparative analysis across hematological malignancies

Across lymphoma and leukemia, EV-associated effects on the B-cell compartment were heterogeneous rather than uniformly immunosuppressive. Reported responses included preferential EV uptake, B-cell proliferation and differentiation, phenotypic reprogramming, and apoptosis. These outcomes are not necessarily contradictory because the studies examined different EV donors, recipient populations and biological endpoints. In particular, evidence that EVs enter B cells should be distinguished from evidence that they produce durable changes in B-cell function.

Lymphoma studies illustrate this context dependence most clearly. MCL-cell preparations displayed preferential tropism toward malignant and non-malignant B cells, but the targeting ligand and biological consequences of uptake were not identified (Hazan-Halevy et al., 2015). EBV-associated preparations instead stimulated normal B-cell proliferation and molecular features of plasmablast differentiation and class-switch recombination. Although LMP1 enhanced these responses, LMP1-negative preparations were also active, indicating that multiple vesicular or co-isolated signals probably contributed (Gutzeit et al., 2014). By contrast, the STAT3–IL-10–PD-L1 study examined malignant T-cell recipients exposed to EVs released by EBV-positive B cells (Chen et al., 2024). It therefore addresses the immunoregulatory activity of B-cell-derived EVs rather than a direct effect on the B-cell compartment and should be interpreted separately from the uptake and B-cell activation studies.

In leukemia, primary CLL-cell sEV-enriched preparations induced broader molecular changes and apoptosis in normal B cells, but the resulting phenotype cannot yet be classified as stable exhaustion or immune tolerance. The simultaneous apoptosis of normal and autologous malignant B cells also complicates a uniformly tumor-promoting interpretation. Possible contributions to hypogammaglobulinemia remain speculative because antibody production, B-cell receptor function and patient immunoglobulin levels were not directly linked to EV exposure (Veletic et al., 2025). The canine study provides weaker B-cell-specific evidence because it reported only a reduced relative B-cell proportion without demonstrating impaired proliferation, apoptosis or effector function (Zmigrodzka et al., 2022).

Thus, evidence is strongest for preferential MCL-EV uptake and for *ex vivo* molecular and apoptotic responses to primary CLL preparations. Evidence for LMP1-specific B-cell activation is moderate, whereas the proposed STAT3-mediated immune-escape mechanism and canine B-cell suppression remain less direct. No study has demonstrated persistent impairment of humoral immunity, an EV-dependent clinical phenotype or prospective biomarker performance. Likewise, the proposed use of MCL EVs for targeted therapeutic delivery remains hypothetical. Future studies should distinguish EV uptake from functional reprogramming and directly assess antibody production, antigen presentation, B-cell receptor signaling and interactions with T cells.

## 11 Tumor microenvironment interaction

Extracellular vesicles suppress anti-tumor immunity in the tumor microenvironment. Tumor-derived EVs contain immunosuppressive proteins (PD-L1, Fas-L, TGF-β, galectins) and miRNAs (e.g., miR-214) that contribute to the induction of T-cell apoptosis, expansion of regulatory T cells, and suppression of cytotoxic CD8^+^ and NK-cell functions. They also impair dendritic cell differentiation and antigen presentation and lead to the accumulation and activation of myeloid-derived suppressor cells (MDSCs) and M2-polarized macrophages by triggering STAT3 and PI3K/AKT signaling. Altogether, these EV-mediated interactions lead to a shifted immune balance in the TME, to the dampening of antitumor immunity, and to the development of resistance to immune checkpoint blockade therapy that targets PD-1/PD-L1 (Tao and Guo, 2020).

### 11.1 Lymphoma

Cell-line sEV-enriched preparations containing PD-L1 inhibited the proliferation of H9 T cells *in vitro*, and genetic alteration of PD-L1 expression in donor DLBCL cells supported a contribution of vesicular PD-L1. Plasma EV-associated PD-L1 was also higher in 26 patients with gastric DLBCL than in 22 healthy controls and was associated with advanced Lugano stage, higher IPI scores and the non-GCB subtype. These clinical associations are potentially relevant but remain cross-sectional. The study did not demonstrate an association with patient survival, treatment response or resistance to PD-1/PD-L1 blockade. Therefore, plasma EV-associated PD-L1 should be regarded as a promising experimental biomarker rather than a clinically validated prognostic or predictive biomarker (Zeng et al., 2023). Several methodological features limit this interpretation. Functional experiments used an immortalized T-cell line rather than primary patient T cells, while plasma EVs were isolated by polymer precipitation and could have included non-vesicular PD-L1 and EVs from immune, platelet and endothelial sources. In addition, tumor-growth experiments compared EV preparations with different PD-L1 expression in NOD-SCID mice. Because these mice lack functional adaptive immunity, increased tumor growth cannot be interpreted as evidence of T-cell suppression *in vivo*. The small, single-center cohort, absence of an independent validation group and lack of reported sensitivity, specificity or predefined cutoffs further restrict the proposed biomarker application (Zeng et al., 2023).

### 11.2 Leukemia

Zannoni et al. (2019) identified a procoagulant microenvironment driven by tissue factor (TF)-bearing sEVs released from clonal monocytes and mesenchymal stromal cells (MSCs) in chronic myelomonocytic leukemia (CMML). The authors used thrombin generation and fibrinography assays to demonstrate that CMML MSCs and monocytes secrete sEVs, vesicles with diameters of ∼80–200 nm, that induce strong thrombin generation and antifibrinolytic activity, whereas healthy controls do not. They also showed that the procoagulant activity is abolished by blocking TF or by using factor VII–deficient plasma and confirmed the presence of TF on sEV membranes by electron microscopy and Western blotting. Functionally, CMML monocyte-derived sEVs were able to transfer TF to healthy MSCs and induce an increase in their procoagulant activity, while CMML MSC sEVs were able to transfer TF to healthy hematopoietic stem cells (HSCs) and increase their thrombin generation and proliferation. Nevertheless, the evidence was obtained largely from small *ex vivo* primary-cell experiments, without animal validation or prospective relationships with thrombosis in patients (Zannoni et al., 2019). AML-cell EV preparations inhibited mitogen-induced T-cell proliferation and reduced NK-cell cytotoxicity against K562 targets. Evidence was strengthened by the use of four AML cell lines, comparisons with donor cells and EV-depleted soluble fractions, and extensive particle characterization using imaging, particle counting and proteomics. However, the experiments involved activated healthy-donor PBMCs and relatively high particle-to-cell ratios rather than purified immune populations under physiological exposure conditions. The magnitude of NK-cell inhibition was moderate, varied among AML cell lines and was not linked to a specific EV cargo or signaling pathway. No animal model or direct assessment of leukemia control was included. However, the very small patient sample and use of third-party immune cells prevent conclusions regarding interpatient variability or autologous immune suppression. The study provides proof-of-concept evidence for immunomodulatory activity but not a clinically validated immune assay or biomarker (Binder et al., 2021). K562-cell sEV-enriched preparations also altered gene expression, cytokine production and redox measurements in cord-blood-derived T cells. Increased FOXP3, PD-1, IL-10, IL-6 and IL-17, together with decreased CD3D, NFATC3, IL-1β and intracellular reactive oxygen species, was interpreted as regulatory and Th17-like reprogramming. However, marker expression alone does not demonstrate differentiation into functional Tregs or suppressive IL-17-producing T cells. No suppression assay, tumor-killing assay or lineage-resolved flow-cytometric analysis was performed. IL-6 and IL-17 may also promote inflammation, making the biological direction of the response less straightforward than a uniformly immunosuppressive interpretation. This evidence is further limited by the use of a single blast-crisis CML-derived cell line, cord-blood T cells and high protein-normalized EV doses. Only three independent experiments were reported, and biological donor variability was unclear. Differential ultracentrifugation produced a 50–200-nm preparation without density purification or negative-marker analysis, so contributions from mixed EV populations and protein aggregates cannot be excluded. These findings remain exploratory cell-culture observations and have not been reproduced using adult patients with CML (Jafarzadeh et al., 2021).

### 11.3 Multiple myeloma

MM-derived EVs reprogram the BM immune microenvironment to favor immunosuppression. The immune cell phenotype of MOPC315.BM-mice carrying MM is bimodal but with a prevailing suppressive profile, as they show a higher expression of PD-1 and CTLA-4 on CD4^+^, CD8^+^, γδ T, and NK cells, decreased CD25 and CD27 and higher transcription of the cytotoxic (Gzmb, Prf1) and the immunoregulatory (Il10, Havcr2) factors, suggesting the coexistence of activated and exhausted immune cells. Conditioning mice with MM-derived EVs before tumor cell implantation recapitulates this immunophenotype, with increased PD-1^+^ and CTLA-4^+^ CD4^+^ T cells and decreased CD27 expression. These data show that MM-derived EVs alone are sufficient to induce an immunosuppressive state. A proteomic approach identified a total of 4,169 EV proteins, of which the LAC2/3, LV2B, and RHEB pathways were enriched, while NDUC2 and ATP2A1/3 were downregulated, suggesting a role for EV cargo in the modulation of NF-κB, mTOR, and apoptotic pathways. The study benefited from an immunocompetent model and density-gradient EV enrichment, but several limitations reduce reproducibility. EVs were repeatedly administered according to protein mass, and the resulting exposure may not reflect endogenous bone-marrow concentrations. Some control animals from different experimental schedules were pooled, while EV-conditioned groups were comparatively small. Proteomic analysis identified 4,169 proteins, but only small numbers of biological samples were used for several comparisons. Changes involving RHEB, NDUC2, ATP2A proteins and immunoglobulin-related proteins are therefore hypothesis-generating and do not establish that these cargos caused the immune phenotype. Bone-marrow EV preparations were also not demonstrated to originate exclusively from malignant plasma cells (Lopes et al., 2022). MM-cell EV-enriched preparations collected from IL-32γ-overexpressing H929 and JJN3 cells increased PD-L1 expression in healthy-donor-derived macrophages. Additional experiments showed that recombinant IL-32γ induced PR3-dependent PFKFB3 expression, glycolytic reprogramming and JAK1-associated PD-L1 upregulation. IL-32γ-treated macrophages suppressed granzyme B, IFN-γ and TNF-α production by autologous CD8^+^ T cells, and PD-L1 blockade partially restored T-cell function. The use of PR3, PFKFB3 and JAK1 perturbations, together with a functional T-cell assay, provides evidence for a biologically coherent macrophage-mediated pathway. Nevertheless, most pathway experiments used recombinant soluble IL-32γ rather than isolated EVs. The study did not demonstrate that depletion of IL-32γ specifically from otherwise comparable EV preparations abolished the macrophage response, and the EV isolation and characterization procedures were insufficiently reported in the supplied article for independent reproduction. Tissue analysis of 16 paired newly diagnosed and relapsed/refractory MM samples showed higher IL-32 and PD-L1 staining and colocalization with CD68^+^ macrophages, but this retrospective observation does not establish that EV-associated IL-32γ caused these changes. It also does not demonstrate treatment resistance or identify a clinically effective target (Liu Y. et al., 2022).

### 11.4 Comparative analysis across hematological malignancies

Across lymphoma, leukemia and MM, EV-associated material generally produced tumor-supportive microenvironmental changes, although the affected processes differed. In lymphoma, EV-associated PD-L1 inhibited T-cell proliferation and correlated with adverse clinical features, but its effects on survival, treatment response and checkpoint-blockade resistance were not examined (Zeng et al., 2023). Leukemia studies were more heterogeneous: CMML-derived TF-bearing sEVs promoted coagulation and stromal–hematopoietic communication, whereas AML- and K562-derived preparations altered T- and NK-cell responses without identifying a dominant immune-suppressive cargo (Zannoni et al., 2019; Binder et al., 2021; Jafarzadeh et al., 2021). MM provided stronger system-level evidence because EV conditioning reproduced several tumor-associated immune changes in immunocompetent mice. The proposed IL-32γ–PFKFB3–JAK1–PD-L1 pathway also linked macrophage reprogramming to impaired CD8^+^ T-cell function, although much of its mechanistic validation used soluble IL-32γ rather than EV-associated cargo (Lopes et al., 2022; Liu Y. et al., 2022).

The strongest causal evidence concerns TF-dependent coagulation in CMML, whereas the MM model provides the most convincing evidence for broader immune-microenvironmental remodeling. EV-specific immune-evasion mechanisms remain less definitive across all three malignancies. Common limitations include cell-line dependence, nonphysiological EV doses, heterogeneous circulating preparations and limited cargo-specific perturbation. Moreover, checkpoint expression and inflammatory cytokine changes do not by themselves establish durable immune suppression. None of the studies demonstrated clinical resistance to PD-1/PD-L1 blockade or prospectively validated an EV-associated biomarker. The evidence therefore supports context-dependent EV-mediated remodeling rather than a universal mechanism of immune escape.

## 12 EVs in anti-tumor response and prognosis

EVs can participate in anti-tumor responses by shuttling tumor antigens and immune-activating signals to antigen-presenting cells, thereby promoting cytotoxic T cell and innate immune surveillance. The molecular composition of circulating EVs mirrors tumor-immune crosstalk and treatment responses, positioning EVs as promising prognostic tools. Broadly, immunostimulatory EV profiles are associated with effective anti-tumor immunity and favorable clinical outcomes. In contrast, EV signatures with an immunosuppressive signature are linked to tumor progression and poor prognosis (Wang et al., 2022).

### 12.1 Lymphoma

In a prospective single-center cohort of 30 patients with relapsed or refractory extranodal NK/T-cell lymphoma, low pretreatment concentrations of soluble and serum EV-associated PD-L1 were associated with response to pembrolizumab and longer time to progression. Four patients achieved complete response, seven achieved partial response and 19 developed progressive disease. Serial measurements also suggested that increasing PD-L1 concentrations accompanied progression, whereas concentrations remained comparatively low in responders. However, only soluble PD-L1 was significantly associated with overall survival; the association between EV-associated PD-L1 and overall survival did not reach statistical significance. Therefore, the two analytes should not be described as having equivalent prognostic performance (Kim et al., 2022). The biomarker findings require cautious interpretation. Serum EVs were isolated by ExoQuick precipitation and could have included EVs from tumor, immune, platelet and endothelial cells, together with lipoproteins and soluble proteins. EV-associated PD-L1 was not significantly correlated with tissue PD-L1 expression, and soluble and EV-associated PD-L1 were strongly correlated with each other, leaving their independent predictive contributions unresolved. Five patients had previously received avelumab, and most of these heavily pretreated, resistant cases had high PD-L1 concentrations, potentially confounding the association. Thresholds were selected within the same small cohort, and sensitivity, specificity, calibration and added value over established clinical predictors were not reported. Thus, EV-associated PD-L1 remains an exploratory treatment-response biomarker rather than a clinically validated predicto (Kim et al., 2022). In a syngeneic murine T-cell lymphoma model, ascites-derived sEV-enriched preparations induced antibodies, tumor-reactive IFN-γ-producing CD4^+^ and CD8^+^ T cells and protection against subsequent LBC lymphoma challenge. Approximately 60% of immunized mice remained tumor-free, and surviving animals resisted homologous rechallenge but not an unrelated mammary tumor, supporting antigen specificity and immune memory. Isolation by ultracentrifugation, sucrose-density purification and detection of ALIX, TSG101, CD9, CD63 and CD81 strengthened the characterization. Nevertheless, the preparations originated from malignant ascites and could have included EVs from nonmalignant cells. CD24 and CD8 enrichment supported, but did not conclusively establish, lymphoma-cell origin. Surface HSP90 and CD24 were proposed as contributors to immunogenicity, but neither molecule was blocked, depleted or genetically manipulated. The study therefore demonstrated protective immunogenicity of the complete preparation rather than causality for a CD24/HSP90 mechanism. In addition, vaccination was prophylactic and was performed before tumor challenge. This design does not establish therapeutic activity against an existing lymphoma, and the results remain limited to a single transplantable murine model (Menay et al., 2017). A subsequent study using the same LBC lymphoma system found that sublethal cyclophosphamide exposure increased EV yield without eliminating immunogenicity. Both untreated and cyclophosphamide-exposed lymphoma-cell sEV preparations induced tumor-specific antibodies, IFN-γ-producing T cells and *ex vivo* cytotoxicity, protecting approximately 70%–90% of mice from subsequent tumor challenge. However, the preparations did not consistently outperform untreated EVs, irradiated lymphoma cells or tumor-cell lysates. The principal finding was therefore increased EV production rather than enhanced vaccine potency (Cocozza et al., 2019). The studies by Menay et al. (Menay et al., 2017) and Cocozza et al. (Cocozza et al., 2019) should not be treated as independent replication because they were conducted by an overlapping research group using the same murine lymphoma model. Both employed prophylactic immunization, and neither evaluated established tumors, clinically scalable manufacturing, product consistency or toxicity. Cyclophosphamide exposure may also alter EV cargo in ways not captured by the limited marker panel. Although apoptotic-body contamination was reduced by selecting a treatment that maintained greater than 80% cell viability and by filtration and ultracentrifugation, it was not definitively excluded. The strongest clinical evidence in this subsection is the longitudinal association between circulating PD-L1 and pembrolizumab response, but the small cohort, data-derived thresholds and nonspecific serum EV isolation prevent clinical validation. Evidence for lymphoma-EV immunogenicity is reproducible within the LBC mouse system but comes from two overlapping studies rather than independent laboratories. The apparent contrast between immunosuppressive PD-L1-bearing EVs and immunogenic lymphoma EV vaccines is readily explained by differences in lymphoma subtype, EV source and mode of exposure. Circulating EVs were studied as components of an active disease environment, whereas purified antigen-bearing preparations were deliberately administered as prophylactic vaccines.

### 12.2 Leukemia

A study by Gargiulo et al. (2023) provided strong preclinical evidence that tissue-derived sEV-enriched preparations promote CLL progression. Preparations from leukemic spleens carried checkpoint ligands, including PD-L1, galectin-9 and VISTA, together with miR-150, miR-155 and miR-378a. They impaired CD8^+^ T-cell metabolism and cytotoxicity, while checkpoint blockade and miRNA antagomirs produced partial functional recovery. Genetic deletion of Rab27a/b delayed leukemia and preserved T-cell activity; EV reintroduction or CD8^+^ T-cell depletion restored disease progression. These complementary perturbations support EV involvement, although splenic preparations contained vesicles from malignant and non-malignant cells. Rab27 deletion can also affect cellular processes beyond EV release and alter the leukemic-cell transcriptome, preventing complete attribution of the phenotype to EVs alone. The accompanying patient analysis showed associations between high cellular expression of EV-biogenesis genes, particularly RAB27A and RAB31, and poor survival. However, the analysis measured gene expression rather than circulating EV abundance or cargo. Candidate signatures were selected and assessed in the same 144-patient cohort without external validation, increasing the risk of overfitting. These signatures therefore remain exploratory prognostic markers (Gargiulo et al., 2023). Conversely, C1498 AML-cell EV preparations stimulated murine splenocyte and CD8^+^ T-cell proliferation, cytokine production and *ex vivo* cytotoxicity against leukemia cells (Pando et al., 2021). Detection of MHC-related proteins was consistent with antigen delivery but did not establish direct EV-mediated antigen presentation. Moreover, increased PD-1, TIGIT and LAG3 indicated that activation was accompanied by checkpoint expression. Because tumor challenge, leukemia burden and survival were not evaluated, the study demonstrates immune stimulation rather than protective antileukemic efficacy (Pando et al., 2021). In CML, monocytic MDSCs were elevated at diagnosis and decreased more substantially during dasatinib treatment than during imatinib or nilotinib treatment. Higher residual M-MDSC levels at 12 months were associated with failure to achieve major molecular response within the small dasatinib subgroup. Serum and EV-enriched preparations from patients also induced M-MDSC-like cells from healthy monocytes. However, treatment allocation was not randomized, and dasatinib can directly affect immune cells independently of EVs. The EV experiments used preparations from only five patients and did not establish their leukemic origin or necessity relative to other serum factors. Thus, the clinically evaluated marker was M-MDSC frequency, not an EV biomarker (Giallongo et al., 2018). Together, leukemia-associated EVs display both immunosuppressive and immunostimulatory effects. Evidence for CLL immune suppression is strongest because it combines cargo interference, EV-release perturbation and *in vivo* disease models. Evidence for AML immune activation is limited to cell culture and *ex vivo* assays. The CML study provides patient observations but weak EV-specific causality. None of the proposed EV signatures has undergone standardized, prospective or independent clinical validation.

### 12.3 Multiple myeloma

MM-cell EV-enriched preparations produced heterogeneous effects on T-cell subsets. Preparations from OPM2 and U266B1 cells increased apoptosis and reduced proliferation in healthy-donor CD4^+^ T cells. They promoted survival of CD8^+^ T cells but reduced perforin expression and enhanced Treg survival and IL-10 production. In Tregs from patients with MM, however, EV exposure reduced viability and TGF-β secretion (Shao et al., 2020). These divergent responses argue against a uniformly immunosuppressive effect. Although the study included 45 patients and 45 controls, the principal functional experiments involved approximately 15 individuals per group. Polymer isolation and high protein-normalized doses also limit physiological interpretation. Cytotoxic killing, suppressive activity, responsible cargo and *in vivo* consequences were not directly evaluated (Shao et al., 2020). Hypoxia increased the release of EV-enriched material and EV-associated miR-1305 from RPMI 8226 myeloma cells. In THP-1-derived macrophage-like cells, the preparations increased TGF-β and CCL2, consistent with, but insufficient to establish, a tumor-supportive polarization state. Transwell and fluorescent-uptake experiments did not exclude soluble mediators or dye-associated artifacts, and the pathway was examined using only one myeloma and one monocyte cell line.

Serum EV-associated miR-1305 was assessed retrospectively in 42 selected patients. High levels showed only a nonsignificant trend toward shorter overall survival in the full cohort, with a significant association restricted to 26 patients receiving bortezomib-based therapy. No multivariable adjustment, external cohort, standardized threshold, sensitivity or specificity analysis was provided. Serum polymer precipitation also recovered heterogeneous circulating material rather than confirmed MM-cell EVs. Consequently, miR-1305 should be described as an exploratory prognostic candidate rather than a validated biomarker (Lee et al., 2021). Therefore, MM-cell EV preparations can alter T-cell and macrophage phenotypes, but the direction of the response varies across recipient populations. Evidence remains largely cell-line-based and is weakened by polymer enrichment, high experimental doses and limited functional validation. The clinical association of EV-associated miR-1305 is subgroup-dependent and requires prospective replication using standardized isolation and quantitative assays.

### 12.4 Comparative analysis across hematological malignancies

Across hematological malignancies, EVs displayed context-dependent immunostimulatory and immunosuppressive activities. In lymphoma, low circulating EV-associated PD-L1 was associated with pembrolizumab response, whereas antigen-bearing EV preparations induced protective immunity in mice (Kim et al., 2022; Menay et al., 2017; Cocozza et al., 2019). These findings are not contradictory: circulating EVs were measured during active disease, while purified preparations were administered as prophylactic vaccines. However, the two vaccine studies used the same model and overlapping investigators and therefore do not constitute independent replication. In leukemia, evidence was strongest for EV-mediated immune suppression in CLL because EV-release and cargo perturbations were combined with *in vivo* disease models (Gargiulo et al., 2023). AML EVs stimulated immune responses *ex vivo* but were not shown to control leukemia, while the CML study evaluated M-MDSC frequency rather than a validated EV biomarker (Pando et al., 2021; Giallongo et al., 2018). MM findings were more heterogeneous, with recipient-dependent effects on T cells and macrophages and no clearly dominant biological direction (Shao et al., 2020; Lee et al., 2021). Clinical translation remains limited across all three malignancies. EV-associated PD-L1 and miR-1305 were evaluated in small cohorts using data-derived thresholds, and neither showed independently validated prognostic or predictive performance. Heterogeneous serum preparations, uncertain cellular origin and polymer-based enrichment further limit biomarker specificity. Likewise, immunogenic EV preparations have shown prophylactic activity only in selected murine models, not therapeutic efficacy against established disease. Current evidence therefore supports EVs as experimental mediators and candidate response markers, but not as validated prognostic assays or clinically established cancer vaccines.

## 13 EVs in regulating immune cells: Emerging therapy strategies

EVs modulating immune cells can be exploited as new cancer therapeutics. EVs can have either immunosuppressive or immunostimulatory effects, depending on their cargo composition and cell of origin. Engineering tumor-derived EVs to present tumor-associated antigens (TAAs), heat shock proteins, or to express immunostimulatory miRNAs can stimulate dendritic cells and T cells, boost antitumor immunity, and be used as a vaccine candidate. Blocking immunosuppressive EVs that express PD-L1, TGF-β, or FasL may help to reverse resistance to immune checkpoint blockade. Immune-cell-derived EVs, for instance, from CAR-T or NK cells, can preserve their cytotoxicity and be exploited as cell-free immunotherapy with less toxicity (Mittal et al., 2020). Figure 9 and Table 2 summarize emerging EV-based immunotherapeutic strategies in hematological malignancies, illustrating how engineered, immune cell-derived, vaccine-based, and natural product-modulated extracellular vesicles can be leveraged to reprogram EV–immune cell interactions and enhance antitumor immunity while limiting immune suppression.

**FIGURE 9:**
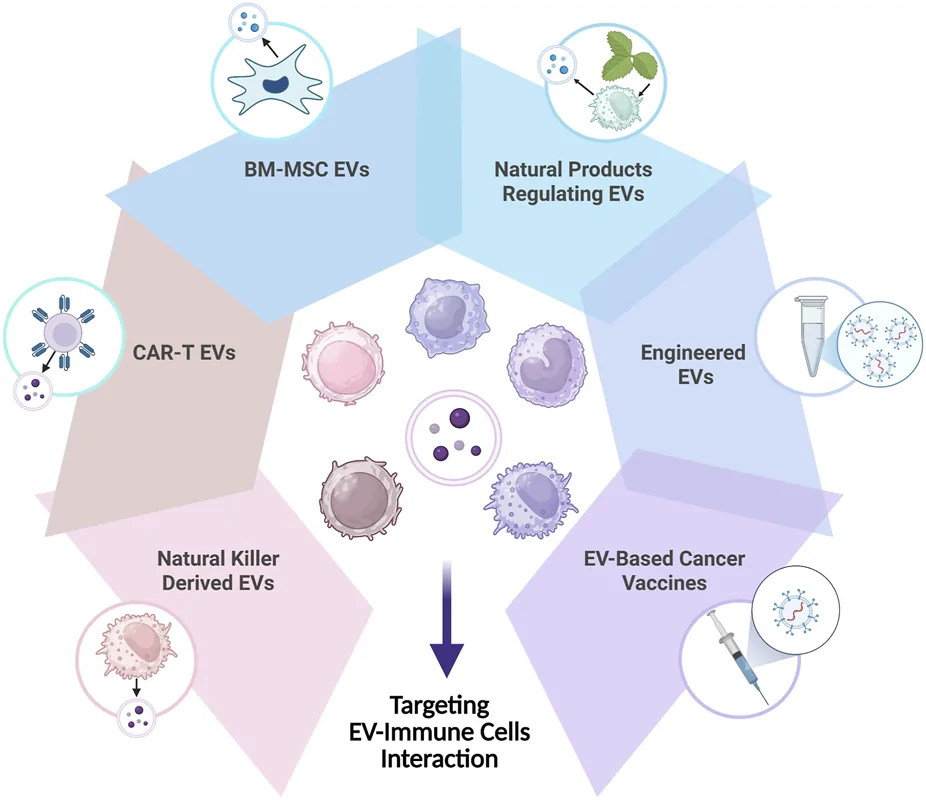
Emerging EV-based therapeutic strategies targeting immune cells, highlighting how engineered EVs, EV-based vaccines, CAR-T/NK-derived EVs, BM-MSC EVs, and natural product modulated EVs can reshape EV-immune cell interactions to enhance antitumor immunity or overcome immune suppression.

**TABLE 2:** Targeting EV interactions with immune cells in hematologic malignancies.

Source of EVs	Type of cancer	Cell lines used	Effect on immune cells	Model	Main results/Findings	Ref.
Red Blood Cell–derived Extracellular Vesicles (REVs) engineered with leukemia-targeting peptide P9	AML	Human AML: THP-1, HL-60; Mouse AML: C1498; AML primary blasts; Control: HS5 (stromal), K562 (CML)	• Induced immunogenic cell death (ICD) (↑CRT exposure, ↑ATP, ↑HMGB1) • Promoted dendritic cell (DC) maturation (↑CD80^+^, CD86^+^) • Enhanced CD8^+^ T-cell infiltration, activation, and IFN-γ/Ki67 expression • Decreased T-cell exhaustion markers (PD-1, TIM-3)	*In vitro*: AML cell lines and primary blasts *In vivo*: AML xenograft (THP-1/NCG mice) and allograft (C1498/C57BL/6 mice)	• Dual-loaded REVs-Dox/siIDO1-P9 specifically targeted CLL-1^+^ leukemia cells • Efficient delivery of Dox (ICD inducer) and siIDO1 (immune checkpoint silencer) • Activated antitumor immunity and reduced immunosuppression • Significantly decreased leukemia burden, prolonged survival, and showed no systemic toxicity	(Liu et al., 2025)
Genetically engineered exosomes from Expi293F cells expressing αCD3–αCLL-1–PD-1–CD70 (PRIME Exos	AML	AML: U937 (CLL-1+++), HL-60 (CLL-1++), KG1A (CLL-1−) T-cells: Jurkat (CD3^+^) PBMCs (human)	• Activated T cells (↑CD69, CD25, IFN-γ, IL-2) • Enhanced CD8^+^ T-cell infiltration and cytotoxicity • Reduced Tregs (↓CD4^+^CD25^+^FoxP3^+^) • Blocked PD-1/PD-L1 inhibitory signaling and stimulated CD27/CD70 costimulation	*In vitro*: Co-culture of AML cells + PBMCs *In vivo*: U937 AML xenograft in NSG mice with human PBMC reconstitution	• PRIME Exos selectively targeted CLL-1^+^ AML cells and activated T cells simultaneously • Significantly increased AML cell killing (EC_50_ ≈ 9.7 ng/mL) • Elevated and sustained IFN-γ/IL-2 secretion • Promoted strong anti-AML immune response with reduced leukemia burden and prolonged survival • No organ toxicity or systemic adverse effects	(Zhang et al., 2025)
Immature dendritic cell–derived exosomes (DC-Exos) genetically modified to express Lamp2b–iRGD fusion protein	DLBCL	OCI-Ly8, OCI-Ly10, SUDHL-10, RPMI-1788 (control B cell line)	Not directly on immune cells (focus on tumor targeting and gene silencing)	*In vitro*: OCI-Ly8 cells *In vivo*: Nude mice bearing OCI-Ly8 xenograft and metastasis models	• iRGD-modified DC-Exos efficiently targeted αvβ3^+^ DLBCL cells • Effective delivery of BCL6 siRNA reduced BCL6 expression and proliferation • Induced G0/G1 cell cycle arrest and increased active caspase-3 in tumors • Suppressed tumor growth and liver metastasis, prolonged survival, and caused no organ toxicity • Demonstrated exosome-mediated siRNA delivery as a safe and precise therapeutic approach for DLBCL	(Liu et al., 2022c)
Dendritic cell–derived exosomes (Dex) loaded with lysates of RAE-1γ–expressing CML cells (BCR-ABL or BCR-ABLT315I)	CML	BP210 (CML) and BP210-T315I (imatinib-resistant CML)	Simultaneously activated NK, CD4^+^, and CD8^+^ T cells via NKG2D/NKG2D-L (RAE-1γ) pathway → ↑CD69, CD137, perforin, granzyme B, IFN-γ, TNF-α, IL-2; ↑proliferation and cytotoxicity	*In vitro*: coculture with murine NK and T cells *In vivo*: Balb/c mouse CML models induced by BCR-ABL or BCR-ABLT315I	• CML-RAE-1γ-Dex strongly enhanced NK and T-cell effector functions • Induced robust cytotoxicity against both WT and T315I-mutant CML cells • Inhibited leukemogenesis, reduced hepatosplenomegaly, and prolonged survival • Generated long-term immune memory upon tumor rechallenge • Activation was NKG2D-dependent, confirming mechanistic specificity	(Du et al., 2022)
Leukemia cell-derived exosomes (LEXs) genetically modified to overexpress CD80 and CD86	Leukemia	Murine L1210 (lymphocytic leukemia) cells	LEX-CD8086 were efficiently taken up by CD4^+^ T cells, transferring exosomal CD80/CD86 and pMHC I; converted CD4^+^ T cells into antigen-presenting, Th1-type cells that secreted IL-2, IFN-γ, TNF-α and strongly stimulated CD8^+^ CTL proliferation and cytotoxicity	*In vitro* assays (CD4^+^ and CD8^+^ T cells) and *in vivo* DBA/2 mouse leukemia models (protective and therapeutic)	CD4^+^ TLEX-CD8086 vaccine markedly inhibited tumor growth, enhanced Th1 cytokines, increased perforin^+^/granzyme B^+^ CD8^+^ T cells, and prolonged survival versus LEX-CD8086 or controls; demonstrates that costimulatory-molecule-modified LEX-targeted CD4^+^ T cells elicit potent anti-leukemia immunity and can act as both APCs and helper T cells	(Hu et al., 2020)
Leukemia cell-derived exosomes (LEXs) genetically modified to overexpress CD80 and CD86 (LEX-CD8086)	ALL	L1210 (murine leukemia) and C1498 (control)	LEX-CD8086 promoted DC maturation, upregulated CD80/CD86 on DCs, and increased secretion of IL-12 and TNF-α. DCsLEX-8086 significantly enhanced CD4^+^ and CD8^+^ T-cell proliferation, increased Granzyme B^+^/Perforin^+^ CTLs, boosted M1 macrophage polarization, and decreased Tregs in tumors	*In vitro* co-culture assays and *in vivo* DBA/2 mouse leukemia models (prophylactic and therapeutic)	DCsLEX-8086 vaccine induced potent antigen-specific CTL responses, markedly inhibited tumor growth, increased T-cell infiltration and M1 macrophages, and prolonged survival. Demonstrated superior anti-leukemia immunity compared to LEX-CD8086 or unmodified DC vaccines	(Zhang et al., 2024)
Leukemia cell-derived exosomes (LEXs) from PD-L1–silenced L1210 cells (LEXPD-L1si)	ALL	Murine L1210 (ALL) and p388 (control)	Downregulation of exosomal PD-L1 enhanced DC maturation (↑CD80, CD86, MHC-II), increased IL-12p70 and TNF-α secretion, and promoted T-cell activation. LEXPD-L1si boosted CD4^+^ T-cell proliferation, Th1 cytokines (IL-2, IFN-γ), and tumor-specific CD8^+^ CTL cytotoxicity	*In vitro* (murine DC and T-cell co-cultures) and *in vivo* DBA/2 mouse leukemia models (preventive and therapeutic vaccination)	LEXPD-L1si vaccination markedly inhibited tumor growth, delayed leukemia progression, and prolonged survival. Protective and therapeutic effects were dependent on CD8^+^ T cells, with CD4^+^ T cells providing helper functions. The study identified exosomal PD-L1 blockade as an effective strategy to reverse immunosuppression a	(Huang et al., 2022)
DLBCL cell–derived exosomes (TEXs)	DLBCL	OCI-LY3, SU-DHL-16, Raji (Burkitt lymphoma comparison), A20 (murine model)	*In vitro*, TEXs promoted DC maturation (↑CD80, CD83, CD86, HLA-DR), enhanced T-cell proliferation, increased IL-6 and TNF-α, and reduced IL-4 and IL-10; however, they also induced T-cell apoptosis and upregulated PD-1, CTLA-4, BTLA, and TGF-β via Fas/FasL/TRAIL signaling	*In vitro* (human DCs, T cells, fibroblasts, HUVECs) and *in vivo* (BALB/c and NOD/SCID mouse models)	DLBCL-derived TEXs had dual effects: they enhanced DC vaccine efficacy and T-cell activation *in vitro*, but promoted tumorigenesis *in vivo* by stimulating stromal proliferation, angiogenesis, invasion, and immunosuppression. Targeting TEX production may improve safety and efficacy of DC-based lymphoma immunotherapy	(Chen et al., 2018)
EVs released from the DCOne allogeneic dendritic cell (DC)-like leukemia cell line	MM	DCOne (AML-derived DC-like line) co-cultured with MM patient PBMCs and autologous MM cells	DCOne-derived EVs transferred tumor antigens (MUC1, survivin) to patient antigen-presenting cells (APCs), stimulating CD8^+^ T-cell activation, IFN-γ and perforin production, and CTL-mediated killing of autologous MM cells, without increasing regulatory T cells or IL-10^+^ CD4^+^ T cells	*In vitro* (patient PBMCs, autologous MM cells)	Demonstrated that DCOne functions as an off-the-shelf allogeneic DC-based vaccine, eliciting cross-presentation of tumor antigens via EVs, leading to myeloma-specific CD8^+^ T-cell immunity and tumor cell killing while maintaining immune balance (no TH2 or Treg expansion)	(Leaf et al., 2017)
Tumor exosomes (TEX) derived from WEHI3B myeloid leukemia and RENCA renal carcinoma cells	Myeloid leukemia and renal cell carcinoma	WEHI3B (murine myeloid leukemia), RENCA (murine renal carcinoma)	TEX enhanced DC maturation (↑CD40, MHC II, IL-12, IFN-γ), promoted CD4^+^ and CD8^+^ T-cell activation, trogocytosis, CTL activity, and cytokine production (IL-2, IFN-γ); TEX uptake occurred efficiently via CD9/CD63/CD81/MFGE8 and supported prolonged MHC II antigen presentation	*In vitro* (murine DC–T-cell coculture) and *in vivo* (BALB/c mouse tumor models)	DC-TEX vaccination significantly prolonged survival and inhibited tumor growth more effectively than DC-lysate. TEX-loaded DCs induced stronger antigen-specific T-cell responses, prolonged antigen presentation, and increased tumor infiltration by T cells, macrophages, and NK cells. TEX were processed in the MHC II-loading compartment (MIIC), leading to superior Th and CTL activation. Findings support TEX as a potent, individualized antigen source for personalized DC-based cancer vaccines	(Gu et al., 2015)
Natural Killer (NK) cell-derived extracellular vesicles (EVs)	Broad range of cancers (solid tumors and hematologic malignancies)	NK92, KHYG-1, HANK1, NKL, NKT, NK-YS, YT (commonly used NK cell lines)	NK-EVs activate dendritic cells, macrophages, and T cells; secrete IFN-γ, TNF-α, CCL3, CCL4, CCL5; and deliver cytotoxic proteins (perforin, granzyme B, FASL) and miRNAs (miR-186, miR-1249-3p) to enhance antitumor immunity and induce tumor apoptosis	*In vitro* (human/murine NK and tumor co-cultures) and *in vivo* (xenograft mouse tumor models)	NK-EVs directly kill tumor cells, modulate the tumor microenvironment, and enhance immune activation. Engineered NK-EVs improve drug delivery, target specificity, and synergistic efficacy when combined with radiotherapy, chemotherapy, photodynamic, photothermal, and immunotherapies. Safe, stable, and highly modifiable, NK-EVs represent a next-generation platform for targeted cancer treatment	(Samara et al., 2023)
Extracellular vesicles (EVs) derived from the immortalized human NK3.3 cell line (NK3.3-LTV)	MM	NK3.3, NK3.3-LTV (hTERT-immortalized NK3.3), MM cell lines RPMI-8226, U266, ARD, ARP, MER, and drug-resistant lines 8,226/LR5 (melphalan-resistant), 8,226/B25 (bortezomib-resistant)	NK3.3-LTV EVs carry NK effector proteins (perforin, granzyme A/B, granulysin) that trigger caspase-3/7 activation and apoptosis in tumor cells. They selectively target and kill MM cells (including resistant subtypes) without harming normal fibroblasts or non-tumorigenic cells	*In vitro* assays using MM cell lines and primary CD38^+^ bone marrow MM cells from patients	NK3.3-LTV EVs significantly inhibited proliferation and induced apoptosis of both drug-sensitive and drug-resistant MM cell lines and primary patient MM cells, mediated via caspase-dependent cell death. EVs retained cytotoxic potency and marker profile of parental NK3.3 EVs but offered continuous, homogeneous EV production due to hTERT immortalization. Importantly, no toxicity was observed in normal cells, highlighting NK3.3-LTV EVs as a safe, reproducible, and scalable immunotherapeutic for refractory MM.	(Matchett and Kornbluth, 2023)
Extracellular vesicles (EVs) derived from healthy human donor NK cells (including both EV and microvesicles)	DLBCL	SU-DHL-4 human B-cell lymphoma	NKEVs carry cytotoxic proteins involved in NK-mediated cytotoxicity and apoptosis pathways; they induce tumor apoptosis (via Bax↑, Bcl-2↓), and alter tumor lipid metabolism, potentially enhancing sensitivity to immune therapies	*In vitro*: SU-DHL-4 co-cultures (24–48 h) *In vivo*: SCID mouse xenograft models (Co-NKEV and Post-NKEV treatment groups)	NKEVs significantly reduced tumor growth (−64% when co-administered; −44% when post-treated) and induced up to 60% apoptotic cell death *in vitro*. MRI/MRS showed increased lipid/lactate and taurine signals, markers of apoptosis, and altered fatty acid composition (↑saturated, ↓unsaturated FAs), indicating disrupted tumor metabolism. Immunofluorescence confirmed reduced CD19, Ki67, and Bcl-2, and increased apoptotic markers. The combined use of NK exosomes and microvesicles had the strongest cytotoxic effect. NKEVs represent a safe, acellular, and natural immunotherapeutic platform capable of restoring immune homeostasis and enhancing antitumor efficacy in lymphoma	(Cecchetti et al., 2024)
EVs derived from human NK cells and IL-2–activated NK cells (LAK-Exo)	AML	KG-1 (human AML cell line)	NK-Exo and LAK-Exo showed direct cytotoxicity against leukemia cells; not primarily studied for immune modulation but represent immune-derived nanovesicles with preserved cytotoxic NK effector molecules (FasL, perforin, granzymes)	*In vitro* (KG-1 co-culture with NK-Exo or LAK-Exo)	Both NK-Exo and LAK-Exo inhibited proliferation of AML cells in a dose- and time-dependent manner. At 50 µg/mL for 48 h, NK-Exo induced 34.6% apoptosis, and LAK-Exo induced 51.6% apoptosis. qPCR and Western blot showed upregulation of CASPASE-3, P38, CYTOCHROME C, and PTEN, along with increased cleaved-PARP and cytochrome C, and reduced p-AKT/p-ERK levels—indicating activation of intrinsic and extrinsic apoptotic pathways. LAK-Exo exhibited stronger antileukemic activity, suggesting NK-derived EVs as potent acellular immunotherapeutics for AML.	(Khatib et al., 2024)
CD19-directed CAR T cells (CAR^+^EVs)	B-cell lymphomas (DLBCL, PMBCL, MCL, etc.)	CD19^+^ K562 (target cells); 19BBζ.CAR T and 19BBζ.eGFP.CAR T cells	CAR^+^EVs mirror CAR T-cell activation; enriched in CD63, CD9, CD8β, PRF1, and ZAP70; lack CD45; indicate cytotoxic activation and tissue homing potential	*In vitro* co-culture (CAR T–target cells); iPSC-derived neural cells; clinical cohort (n = 100)	CAR^+^EVs released within 1 h of target engagement; early elevation (>132.8/μL at 1 h or >224.5/μL at day 1) predicted ICANS 4 days before onset; associated with IL-15 and CX3CL1 signaling; induced ENO2^+^ nanoparticle and miR-1246 release from neurons, marking neural stress	(Storci et al., 2024)
Lymphoma-derived extracellular vesicles (TEXs)	B-cell malignancies (Burkitt lymphoma, DLBCL, ALL)	Raji, Nalm-6, OCI-LY3	TEXs activate CD19-CAR T cells via exosomal CD19 but chronic exposure impairs CAR T effector function and promotes Treg-like phenotype through exosomal TGF-β	*In vitro* (CAR T – tumor co-culture); *in vivo* murine xenograft	Exosomal CD19 primes CAR T activation; prolonged TEX exposure leads to exhaustion and Treg conversion. Blocking TGF-β signaling (LY2109761) restores CAR T cytotoxicity and tumor clearance	(Hao et al., 2024)
Bone marrow mesenchymal stem cell (BM-MSC)-derived EVs	AML	HL60 (AML cell line); CD4^+^ T cells	Increased Th1/Th2 ratio by enhancing IFN-γ and reducing IL-4; promoted CD4^+^ T cell differentiation toward Th1 phenotype	*In vitro* (HL60 cells + CD4^+^ T cells) and *in vivo* (SCID mice AML model)	BM-MSC EV miR-222-3p targets IRF2 to upregulate Th1/Th2 ratio and caspase-3-mediated apoptosis in AML cells. IRF2 overexpression reverses these effects. Overexpression of miR-222-3p in BM-MSC exosomes enhances antitumor immunity and apoptosis, suggesting its therapeutic potential in AML.	(Yuan et al., 2023)
Guizhi Fuling Decoction	MM	U266 (human MM cell line), RAW264.7 (murine macrophage cell line)	MM-derived EVs promoted osteoclast differentiation, bone resorption, and increased RANKL expression in macrophage-derived osteoclast precursors	*In vitro* (RAW264.7 + MM EVs, GW4869 inhibition), *in vivo* (U266 xenograft in NOG mice), and clinical trial (MM patients with bone lesions)	GZFL (Guizhi Fuling Decoction) and its active compounds (paeoniflorin, quercitrin, kaempferol) targeted and suppressed exosomal ERK1 from MM cells, thereby inhibiting RANKL-induced ERK1 activation and osteoclastogenesis. In mice, GZFL reduced bone destruction and improved survival; clinically, GZFL + PD (bortezomib/dexamethasone) lowered ERK1/RANKL and bone pain, showing safety and efficacy	(Yu et al., 2025)
Sophora flavescens-derived extracellular vesicles (SF-EVs)	DLBCL	OCI-LY10, SU-DHL-2	Activated tumor-associated macrophages (TAMs) and promoted antigen presentation; enhanced CD8^+^ T-cell infiltration	*In vitro* and xenograft DLBCL mouse models	SF-EVs served as biocompatible carriers for oncolytic vaccinia virus (OVV) and NIR-II probe IR1061. The hybrid EVs (SF-EV@OVV/IR1061) enabled tumor-targeted accumulation, real-time NIR-II imaging, and synergistic oncolytic/immunogenic therapy. They inhibited tumor growth, reprogrammed TAMs toward M1 phenotype, and promoted CD8^+^ T-cell–mediated antitumor immunity	(Ji et al., 2025)

### 13.1 Engineered EVs

EVs play a pivotal role in hematological malignancies by altering the tumor microenvironment through immune suppression or activation, stromal reprogramming, and inducing or sustaining chemoresistance. Oncogenic or suppressive cargo they carry make them disease drivers and thus therapeutic targets as well. Engineered EVs can be used to directly deliver tumor-suppressive miRNAs or drug cargos to cancer cells, promote antitumor immunity through loading with costimulatory molecules such as CD80/CD86, or reverse chemoresistance by fusion inhibitor proteins that block EV uptake. EVs from CAR-T cell–engineered EVs (CAR-EVs) retain selective cytotoxicity and represent a potent cell-free immunotherapy with safety advantages (Van Morckhoven et al., 2023). Two recent AML studies used distinct EV-engineering strategies to combine leukemia targeting with immune activation. Red blood cell-derived EVs (REVs) were functionalized with the CLL-1-binding P9 peptide and loaded with IDO1-targeting siRNA and doxorubicin, an anthracycline topoisomerase-II inhibitor used here to induce immunogenic cell death. The combined formulation enhanced delivery to CLL-1-positive AML cells, reduced IDO1 expression and increased calreticulin exposure, ATP/HMGB1 release, DC maturation and CD8^+^ T-cell activity. In immunocompetent C1498 AML models, six intravenous doses containing 3 mg/kg doxorubicin and 5 nmol/kg siIDO1 reduced leukemic burden and prolonged survival relative to either cargo alone (Liu et al., 2025). The inclusion of single-cargo, scrambled-peptide and siRNA controls, primary AML blasts and an immunocompetent model supports both targeting and combination effects. However, most mechanistic experiments used cell lines, the number and clinical characteristics of primary samples were insufficiently reported, and the P9 platform was not tested across heterogeneous CLL-1 expression patterns. Safety assessment involved only five mice per group and short-term histology and laboratory measurements; therefore, the formulation cannot yet be described as having established biosafety, particularly given the potential cumulative cardiotoxicity of doxorubicin (Liu et al., 2025). PRIME Exos used a different design, displaying anti-CD3 and anti-CLL-1 single-chain antibodies together with PD-1 and CD70 to connect T cells with AML cells while modulating PD-1/PD-L1 and CD27 signaling. The preparations selectively activated PBMCs and killed CLL-1-positive U937 and HL60 cells, with little activity against CLL-1-negative KG1A cells. In NSG mice receiving human PBMCs and U937 cells, PRIME Exos reduced leukemia expansion, increased CD8^+^ T-cell proportions and decreased Treg frequencies (Zhang et al., 2025). Nevertheless, the *in vivo* experiments included only five mice per group, ended approximately 15 days after leukemia inoculation and did not assess survival or durable disease control. The xenograft system also lacks an intact human immune and marrow microenvironment. Moreover, transient co-transfection and bulk immunoblotting do not establish uniform co-display of all four functional molecules on every vesicle. Batch heterogeneity, cytokine-release risk, activity against normal CLL-1-expressing myeloid cells and large-scale manufacturing remain unresolved. These AML platforms therefore provide complementary but not directly comparable evidence. The REV formulation combines drug delivery with endogenous antitumor immunity in an immunocompetent murine model, whereas PRIME Exos function principally as multivalent immune-cell engagers in a humanized xenograft. The former provides stronger survival and immune-mechanistic evidence, while the latter demonstrates greater molecular programmability but has more limited *in vivo* follow-up. Neither has been independently reproduced or evaluated using patient-derived xenografts containing diverse AML subclones (Zhang et al., 2025). In DLBCL, murine DC-derived sEV-enriched preparations were engineered to display iRGD–Lamp2b and loaded with BCL6 siRNA by electroporation. The preparations preferentially entered αvβ3-high OCI-Ly8 cells, reduced BCL6 expression, induced G0/G1 arrest and inhibited proliferation. Repeated intravenous administration in nude-mouse OCI-Ly8 models reduced tumor burden and liver nodules and was associated with longer survival (Liu Q. et al., 2022). Targeted and non-targeted EV controls support an iRGD-dependent delivery advantage. However, efficacy was examined using only one DLBCL cell line, without primary tumors or immunocompetent models. αvβ3 dependence was inferred from expression and uptake rather than confirmed through receptor blockade or genetic loss of function. Furthermore, the preparations accumulated substantially in the liver and kidneys, and the claim of safety relied mainly on short-term organ histology. Electroporation may also cause siRNA aggregation, while the proportion of siRNA encapsulated within, rather than externally associated with, EVs was not rigorously established (Liu Q. et al., 2022). A vaccine-based strategy was evaluated in CML using DC-derived sEV-enriched preparations generated after pulsing DCs with lysates from RAE-1γ-expressing BP210 or T315I-mutant BP210 cells. These preparations activated murine NK, CD4^+^ and CD8^+^ T cells and increased cytotoxicity against the corresponding leukemia cells. NKG2D blockade attenuated, but did not eliminate, immune activation, supporting a partial contribution of the RAE-1γ–NKG2D pathway. In syngeneic mouse models, vaccination reduced CML manifestations, prolonged survival and protected surviving animals against rechallenge (Du et al., 2022). The combination of pathway blockade, wild-type and T315I models and rechallenge experiments strengthens preclinical evidence. Nevertheless, group sizes were not clearly reported in the main article, and the platform was entirely murine. RAE-1γ is a murine NKG2D ligand; translation would require redesign using human ligands such as MICA/B or ULBPs, whose prolonged presentation may also cause NKG2D downregulation rather than activation. The net effect may therefore depend on dose and exposure duration (Du et al., 2022). Leukemia-cell sEV-enriched preparations were also modified indirectly by silencing TGF-β1 in L1210 donor cells. Compared with unmodified or control-shRNA preparations, TGF-β1-reduced sEVs enhanced DC maturation, Th1 cytokine production and CTL and NK-cell activity. Preventive and therapeutic vaccination reduced tumor growth and prolonged survival in DBA/2 mice. Genetic cargo modification, control-shRNA preparations and ten-animal groups support a causal contribution of TGF-β1. However, TGF-β1 was reduced rather than eliminated, and other cargo changes caused by donor-cell transduction were not comprehensively assessed. Only 10% of treated tumor-bearing mice survived to the end of the 50-day observation period, showing that TGF-β1 reduction alone was insufficient for durable control. The findings are also limited to a single murine lymphocytic-leukemia model (Huang et al., 2017b). Together, engineered EVs can be divided into cargo-delivery systems, multivalent immune engagers and modified tumor-antigen vaccines. Across these approaches, targeting controls, genetic or molecular perturbations and animal experiments provide convincing proof of concept. However, the evidence remains exclusively preclinical and relies predominantly on cell lines and small mouse groups. Cross-study comparison is restricted by different EV sources, isolation methods, dosing units and disease models. No platform has established reproducible large-scale production, batch-to-batch molecular uniformity, storage stability, human pharmacokinetics, long-term toxicity or superiority over non-EV delivery systems. Consequently, these formulations should be presented as experimental therapeutic platforms rather than clinically validated EV therapies.

### 13.2 EV-based cancer vaccines

Bioengineered EVs have recently been identified as a next-generation class of cancer vaccine platforms, with unique advantages of natural biocompatibility, immunogenic versatility, and engineering flexibility. EVs that can be engineered to carry tumor-associated antigens, adjuvant molecules, and immunoregulatory cues in a single nanostructure are derived from a variety of cell types, including dendritic cells, macrophages, lymphocytes, or tumor cells. The EVs can then be further engineered either genetically or chemically. As delivery vehicles, immune activators, and antigen-presenting entities, these engineered EVs prime CD8^+^ cytotoxic and CD4^+^ helper T-cell responses (Ahn et al., 2025). In the murine L1210 leukemia model, donor cells were engineered to overexpress CD80 and CD86, producing sEV-enriched preparations termed LEX-CD8086. These preparations enhanced DC maturation and leukemia-specific T-cell responses. Hu et al. (Hu et al., 2020) demonstrated prophylactic activity, whereas Li et al. (Li et al., 2022) loaded LEX-CD8086 onto activated CD4^+^ T cells to generate hybrid CD4^+^ TLEX-CD8086 cells with helper and antigen-presenting properties. These cells increased Th1 cytokine production and CD8^+^ cytotoxicity in preventive and therapeutic models. However, the evidence that CD4^+^ T cells acquired functional peptide–MHC I and costimulatory complexes was based mainly on uptake and phenotypic assays. Both studies used the same L1210 platform and overlapping investigators and therefore should not be considered independent replication. Moreover, protection was incomplete, all therapeutically treated mice in the later study eventually died, and neither approach was tested using human leukemia cells (Hu et al., 2020; Li et al., 2022). Zhang et al. (Zhang et al., 2024) extended the same platform by loading LEX-CD8086 onto murine bone-marrow DCs. The resulting DCsLEX-CD8086 increased DC maturation, IL-12p70 and TNF-α secretion, and leukemia-specific CD8^+^ T-cell cytotoxicity. Preventive and therapeutic vaccination reduced tumor growth, increased CD4^+^ and CD8^+^ T-cell and M1-macrophage proportions, and decreased Tregs in established tumors (Zhang et al., 2024). Nevertheless, each animal group contained only five mice, the therapeutic median survival remained 33 days, and the experiments again relied on subcutaneous L1210 tumors rather than a disseminated leukemia model. Because the study used the same engineered donor-cell system as Hu et al. (Hu et al., 2020) and Li et al. (Li et al., 2022), it demonstrates extension to a DC vaccine rather than independent validation across leukemia models (Zhang et al., 2024). A complementary strategy involved removing inhibitory EV cargo. Huang et al. (Huang et al., 2022) silenced PD-L1 in L1210 donor cells and obtained sEV-enriched preparations with reduced PD-L1. These preparations enhanced DC maturation, Th1 cytokine production and leukemia-specific cytotoxicity and delayed tumor growth in preventive and therapeutic models. CD8^+^ depletion substantially reduced efficacy, supporting a role for cytotoxic T cells, with additional CD4^+^ T-cell assistance. The use of control shRNA preparations, direct PD-L1 measurement and lymphocyte-depletion experiments strengthens the mechanistic evidence. However, donor-cell PD-L1 silencing may have altered other cellular and vesicular components, and the preparations could also have transferred shRNA or siRNA to recipient cells. The study therefore does not isolate reduced vesicular PD-L1 as the only causal mechanism. It also originated from the same L1210 research platform and lacks human validation (Huang et al., 2022). DC loading may partly separate the antigenic and immunosuppressive functions of tumor EVs. In WEHI3B myeloid leukemia and RENCA renal carcinoma models, Gu et al. (Gu et al., 2015)found that DCs loaded with tumor-cell sEV-enriched preparations produced stronger survival and antigen-specific responses than lysate-loaded DCs. The preparations were efficiently internalized and showed prolonged association with MHC-II-loading compartments, providing a plausible explanation for enhanced CD4^+^ T-cell activation and subsequent CTL responses (Gu et al., 2015). Density-gradient purification and comparison across two tumor models strengthen the study. Nevertheless, only one model represented hematological malignancy, several mechanistic conclusions relied on fluorescent-label colocalization, and some *in vitro* CTL measurements did not clearly distinguish EV-loaded from lysate-loaded DCs (Gu et al., 2015). Zhenzhen Chen and colleagues (Chen et al., 2018) directly illustrated the context dependence of this approach. DLBCL-cell sEV-enriched preparations promoted maturation and T-cell-stimulatory activity when loaded onto DCs *in vitro*, whereas free preparations induced T-cell apoptosis, increased inhibitory signaling and promoted stromal-cell invasion, angiogenesis and tumor growth. The apparent contradiction is therefore biologically plausible: controlled uptake and antigen processing by mature DCs can favor antigen presentation, while unrestricted exposure of T cells and stromal cells can transmit suppressive or tumor-promoting cargo. However, the human experiments used cell lines rather than primary patient cells, and the immune-vaccination studies were performed with murine A20 lymphoma EVs and DC lines. Tumor-promotion experiments used immunodeficient mice, preventing assessment of adaptive immune suppression *in vivo*. The study consequently supports context-dependent activity but not the clinical safety of DLBCL EV-loaded vaccines (Chen et al., 2018). Another group of researchers evaluated a different concept using DCOne, an allogeneic AML-derived DC-like cell line expressing antigens shared with multiple myeloma, including MUC1 and survivin. Coculture with PBMCs from patients with MM increased IFN-γ- and perforin-positive CD8^+^ T cells, and stimulated PBMCs showed increased killing of autologous myeloma cells. EV-enriched preparations from DCOne contained MUC1 and survivin, supporting EV-mediated antigen transfer to recipient antigen-presenting cells (Leaf et al., 2017). However, DCOne was administered as a whole-cell vaccine in the functional experiments; isolated EVs were not independently tested as a vaccine. The principal cytotoxicity analysis involved only three patients, and EVs were obtained using polymer precipitation without comprehensive particle characterization. This study therefore provides human *ex vivo* evidence for EV-assisted antigen transfer, but not evidence for the efficacy of a DCOne EV vaccine in patients (Leaf et al., 2017). These studies support two complementary principles: increasing costimulatory cargo or reducing inhibitory cargo can enhance the immunogenicity of leukemia-cell EV preparations, and loading tumor EVs onto mature DCs can redirect antigenic material toward productive presentation. The strongest causal evidence comes from genetic modification and immune-cell-depletion experiments, but much of it originates from the same L1210 model and overlapping research groups. Gu et al. and Chen et al. provide broader support for DC-mediated antigen processing, while also showing that free tumor EVs may remain immunosuppressive or tumor-promoting. Leaf et al. add human *ex vivo* relevance but do not evaluate isolated EVs as the therapeutic product. No strategy has yet demonstrated reproducible clinical efficacy, standardized manufacturing or safety across independent hematological-malignancy models.

### 13.3 Natural killer EVs

NK cell-derived EVs exhibit potent anticancer activity by carrying cytotoxic molecules such as perforin, granzymes, and FASL, which directly induce apoptosis in tumor cells. In addition, they contain cytokines (IFN-γ, TNF-α), chemokines (CCL3, CCL4, CCL5), and miRNAs that modulate the tumor microenvironment, enhance dendritic cell and T-cell activation, and suppress immunosuppressive signaling pathways. Owing to their high safety, stability, and modifiability, NK-EVs can also serve as drug delivery platforms, engineered to target specific tumors and release therapeutic cargo precisely, often in synergy with radiotherapy, chemotherapy, or photodynamic therapy (Si et al., 2024). sEV-enriched preparations isolated from the NK92MI cell line demonstrated dose-dependent apoptosis and cytolysis across several leukemia cell lines. Activity was also observed in primary samples from patients with AML, ALL, CLL and CML, whereas an immortalized B-cell line and healthy-donor PBMCs were comparatively resistant. Caspase inhibition reduced cytotoxicity, and activity co-segregated with particle-containing density-gradient fractions, strengthening its attribution to EV-associated material. In an HL-60 xenograft model, only three consecutive administrations of the highest dose reduced circulating human CD45^+^ cells and modestly improved survival; lower-dose and single-dose regimens did not improve survival (Samara et al., 2023). EVs derived from the immortalized human NK cell line NK3.3-LTV are a novel therapeutic with high potential to treat multiple myeloma (MM). NK cell–based therapies can have some drawbacks, including donor-dependent cell variability, limited *in vivo* lifespan, and high risk of graft-versus-host disease and graft rejection for allogenic donor cells. The development of a standardized, acellular NK-EV therapy would enable consistent production and off-the-shelf availability, while NK3.3 EVs are enriched in NK cytotoxic proteins (perforin and granzymes). The study found that EVs derived from immortalized NK3.3 cells had a similar protein profile, purity, and tumor-killing activity as non-immortalized NK3.3 cells, while immortalization allows for continuous expansion and scalable manufacturing for off-the-shelf availability. NK3.3-LTV EVs reduced proliferation and induced caspase-dependent apoptosis in both drug-sensitive and drug-resistant MM cell lines (melphalan- and bortezomib-resistant) as well as primary patient MM cells with no toxicity on normal human cells (Matchett and Kornbluth, 2023). Cecchetti et al. (Cecchetti et al., 2024) separately collected 10,000 × g microvesicle-enriched and 100,000 × g sEV-enriched fractions from activated NK cells expanded from at least ten healthy donors. Both fractions reduced SU-DHL-4 lymphoma-cell viability, with the strongest effect after combined administration. In SCID mice, repeated intratumoral treatment with the combined preparation reduced tumor growth, while MRI, magnetic resonance spectroscopy and tissue analyses showed changes compatible with reduced proliferation and increased cell death. Use of primary donor NK cells, separate EV fractions and multimodal tumor assessment are strengths. However, the fractions were defined mainly by centrifugation speed and size and were not purified sufficiently to establish their biogenesis. The 10,000 × g fraction may contain organelle fragments or apoptotic material, while the 100,000 × g fraction may contain small plasma-membrane-derived EVs and protein aggregates. Animal groups contained seven mice, and the treatment was administered intratumorally; one group also received EVs simultaneously with tumor-cell implantation. These designs do not reproduce systemic treatment of established lymphoma. The reported lipid, lactate, taurine and fatty-acid changes are compatible with altered tumor homeostasis but are not specific evidence that EVs directly reprogrammed tumor metabolism. Survival, systemic toxicity and activity against primary lymphoma samples were not evaluated (Cecchetti et al., 2024). Another group of researchers compared preparations from resting peripheral-blood NK cells with those from IL-2-activated lymphokine-activated killer cells. Both reduced KG-1 AML-cell proliferation and induced apoptosis, with greater activity from activated-cell preparations. At 50 μg/mL, total apoptosis reached approximately 35% with resting NK-cell EVs and 52% with activated NK-cell EVs. Increased CASPASE-3, P38 and CYTOCHROME C transcripts, together with cleaved PARP and cytochrome-c protein changes, were consistent with apoptotic signaling. These findings suggest that donor-cell activation state influences EV potency. The mechanistic conclusions require caution. NK cells were obtained from only two donors, EVs were isolated with a commercial precipitation kit, and characterization relied primarily on morphology, dynamic light scattering and CD63 without an adequate panel of positive and negative EV markers. Only one AML cell line was studied, with no primary AML samples, normal-cell comparator or animal validation. Moreover, reductions in AKT and ERK transcripts were not statistically significant, p-AKT reduction was not confirmed by immunoblotting, and p38 transcript and protein results were discordant. The study therefore supports greater *in vitro* cytotoxicity after NK-cell activation but does not establish coordinated suppression of AKT/ERK signaling or simultaneous activation of fully defined intrinsic and extrinsic apoptotic pathways (Khatib et al., 2024).

Collectively, these studies consistently show that NK-cell EV-enriched preparations can induce apoptosis or cytolysis in leukemia, lymphoma and MM cells. Evidence is strongest for short-term, dose-dependent *in vitro* cytotoxicity and is supported by limited primary-cell experiments. Samara et al. and Cecchetti et al. add animal evidence, but efficacy depended on intensive high-dose or local treatment, and neither study established systemic safety in an immunocompetent model. Differences in donor source, activation conditions and isolation procedures probably explain variations in potency: preparations were derived from NK92MI cells, hTERT-immortalized NK3.3 cells, IL-2-activated primary NK cells or feeder-expanded primary NK cells. These products should not be treated as interchangeable. The available data do not yet demonstrate that NK-EV therapy overcomes the clinical limitations of NK-cell infusion. Scalable good-manufacturing-practice production, batch consistency, potency assays, tumor targeting, pharmacokinetics, biodistribution, repeated-dose toxicity and the exclusion of oncogenic cargo remain unresolved. No study reported administration to patients with a hematological malignancy.

### 13.4 CAR-T-EVs

CAR-T-derived extracellular vesicles (CAR-T-EVs), a new cell-free immunotherapy platform, inherit the tumor-targeting ability of CAR-Ts and show safety and delivery advantages. Loaded with cytotoxic cargo such as perforin and granzymes, CAR-T-EVs can cross tumor barriers, resist immunosuppression, and kill antigen-positive tumor cells. Unlike CAR-Ts, CAR-T-EVs do not cause cytokine release syndrome. CAR-T-EVs are more stable and have low immunogenicity. They can also be prepared off the shelf, making it a promising way to surpass CAR-T therapy limitations, especially in solid tumors (Pagotto et al., 2023). CAR-positive circulating EVs were evaluated as early biomarkers of immune effector cell-associated neurotoxicity syndrome (ICANS) in a prospective cohort of 100 patients with B-cell lymphoma receiving CD19-directed CAR T cells. Twenty-nine patients developed ICANS. CAR-positive EVs appeared within 1 h of antigen engagement *in vitro* and were detectable in plasma 1 h after infusion. Concentrations above 132.8 EVs/μL at hour +1 predicted ICANS with 89.3% sensitivity and 74.6% specificity, whereas concentrations above 224.5 EVs/μL at day +1 achieved 96.6% sensitivity and 80.4% specificity. These particles carried the CAR construct together with CD63, CD9, CD8β, ZAP70 and perforin, supporting their relationship to activated CAR T cells. Exposure of induced pluripotent stem cell-derived neural cells to sorted CAR-positive EVs caused limited cell death but stimulated the release of ENO2-positive nanoparticles and miR-1246, which were also increased in patients with ICANS. The prospective design, relatively large cohort and multimodal particle analysis strengthen the biomarker evidence. However, the predictive thresholds were derived and evaluated within the same single-center cohort and require external validation across CAR constructs, products and analytical platforms. Moreover, the neuronal experiments demonstrate cellular stress rather than clinical neurotoxicity, while associations with IL-15, CX3CL1 and ENO2-positive nanoparticles do not establish that CAR-positive EVs cause ICANS (Storci et al., 2024). Tumor-derived sEV-enriched preparations exerted time-dependent effects on CD19-specific CAR T cells. Short exposure to CD19-positive preparations from B-cell malignancy cell lines increased CAR-T activation, proliferation, cytokine release and cytotoxicity; CD19-knockout preparations weakened these responses, supporting antigen-dependent activation. In contrast, prolonged exposure increased apoptosis, PD-1, TIM-3 and LAG-3 expression, reduced naïve and central-memory phenotypes and impaired sustained cytotoxicity. Single-cell RNA sequencing of CAR T cells from three donors further identified loss of naïve/memory transcriptional programs and enrichment of differentiated or exhaustion-associated states. TGF-β was detected in the preparations, and inhibition of TGF-β–Smad3 signaling partially reversed FOXP3 induction, checkpoint expression and terminal differentiation while improving CAR-T activity in a Raji xenograft model. Nevertheless, the inhibitor was administered systemically and was not specific to EV-associated TGF-β. FOXP3 expression also does not by itself demonstrate conversion into stable, suppressive Tregs. Patient-plasma experiments involved limited samples and heterogeneous circulating EVs that could not be assigned exclusively to lymphoma cells. The study therefore supports a biphasic, exposure-dependent interaction between lymphoma-cell EV preparations and CAR T cells, but not a clinically established mechanism of CAR-T resistance (Hao et al., 2024). A related strategy used αCD3 × αPD-L1 bispecific T-cell engager-armed T cells to exploit PD-L1-positive tumor-cell EVs as potential trafficking signals. PD-L1-positive preparations enhanced T-cell migration in transwell and microfluidic assays, whereas PD-L1 knockout, antibody competition and GW4869 treatment reduced migration. Bispecific-engager-armed T cells showed increased tumor infiltration and antitumor activity in murine models, including a human multiple-myeloma xenograft. The approach was also applied to CD19 CAR T cells, improving their infiltration and activity in mice bearing MC38 cells engineered to express human CD19. However, the multiple-myeloma experiments used bispecific-engager-armed PBMCs rather than CAR T cells, and the CAR-T model involved an artificially modified solid-tumor cell line rather than a hematological malignancy. GW4869 is not an EV-specific inhibitor, and PD-L1 knockout affects both cellular and vesicular PD-L1. Thus, the evidence supports an experimental EV-associated trafficking mechanism but does not demonstrate improved CAR-T delivery in lymphoma or myeloma models representative of clinical disease (Cho et al., 2024). These studies illustrate three distinct functions of EVs in CAR-T therapy: circulating indicators of early CAR-T activation, tumor-derived modulators of CAR-T function and potential trafficking signals for engineered T cells. Evidence is strongest for the association between early plasma CAR-positive EV concentrations and subsequent ICANS, although the proposed thresholds require independent validation. Evidence for tumor-EV-mediated CAR-T dysfunction is mechanistically plausible but remains dependent on cell-line preparations, high experimental doses and non-EV-specific pathway inhibition. The trafficking strategy is conceptually promising but was tested mainly in engineered or solid-tumor models. None of studies establishes the safety or efficacy of administering CAR-T-derived EVs as an off-the-shelf therapeutic product.

### 13.5 Mesenchymal stem cell

Bone marrow mesenchymal stem cell (BM-MSC) EV-enriched preparations from healthy donors increased the reported Th1/Th2 ratio, IFN-γ production and HL60-cell apoptosis more strongly than preparations from patients with AML. MiR-222-3p was reduced in patient-derived preparations and directly targeted IRF2 in luciferase assays. MiR-222-3p mimics decreased IRF2 and increased caspase-3 expression and apoptosis, whereas inhibition of miR-222-3p produced the opposite effects. IRF2 overexpression partially reversed these responses, supporting a miR-222-3p–IRF2 mechanism. However, the MSC identity of the donor cells was not adequately verified, and precipitation-based isolation could have recovered non-vesicular material. More importantly, isolated preparations were incubated directly with miRNA mimics or inhibitors without demonstrating vesicular encapsulation or removing unbound oligonucleotides. The effects therefore cannot be attributed confidently to EV-mediated delivery. The *in vivo* findings are also difficult to interpret because SCID-beige mice lack normal adaptive immunity, no human T-cell reconstitution was described, and leukemia burden or survival was not measured. Thus, the study provides exploratory evidence for a miR-222-3p–IRF2 axis but does not establish that BM-MSC EVs suppress AML progression(Yuan et al., 2023).

### 13.6 Natural products in regulating EVs

Natural products can also be employed to regulate EV biogenesis, cargo loading, and secretion, which in turn modulates immune regulation in the cancer setting. Curcumin and other phytochemicals have been demonstrated to alter TDEV composition and downstream signaling functions, ultimately decreasing immunosuppression and restoring anti-tumor immunity. Natural compounds have been found to modulate miRNA and protein cargos in EVs involved in antigen presentation, cytokine signaling, and T-cell activation, for example, leading to restored immune surveillance and increased dendritic cell and T-cell responses (Xu et al., 2022). Guizhi Fuling decoction (GZFL), a traditional herbal formulation containing *Cinnamomi Ramulus*, *Cortex Moutan*, *Poria*, *Radix Paeoniae Rubra* and *Persicae Semen*, was evaluated for its effects on MM-associated bone destruction. MM-cell sEV-enriched preparations transferred ERK1-related signaling to osteoclast precursors and enhanced RANKL-dependent ERK activation, osteoclast differentiation and bone resorption. Pretreatment of MM cells with GZFL-containing serum reduced ERK1 abundance in the resulting preparations. Three identified constituents, paeoniflorin, quercitrin and kaempferol, also bound ERK1 and reproduced parts of this effect, although binding alone does not establish direct enzymatic inhibition. Preparations collected from treated MM cells consequently produced weaker osteoclastogenic responses. In myeloma-bearing mice, GZFL reduced osteoclast formation, preserved bone microarchitecture and decreased bone-marrow ERK1 and RANKL expression, supporting activity within the myeloma–bone microenvironment (Yu et al., 2025). The human findings in study (Yu et al., 2025) require more cautious interpretation. Bone pain and ERK1/RANKL expression decreased in a small single-arm cohort receiving GZFL alongside bortezomib- and dexamethasone-based treatment, bisphosphonates and, in some patients, additional antimyeloma agents. Without a comparator group, the clinical improvement cannot be attributed specifically to GZFL or suppression of EV-associated ERK1. Moreover, the main functional experiments used U266 cells and murine osteoclast precursors. Although differential centrifugation and ultracentrifugation, TEM and particle-size analysis supported enrichment of vesicular material, exclusive exosomal biogenesis was not demonstrated. The findings therefore support a preclinical mechanism involving MM-cell sEV-associated ERK1 but do not establish an EV-directed clinical therapy (Yu et al., 2025). Omega-3 polyunsaturated fatty acids were examined as another means of altering the immunological activity of MM-cell EVs. EV-enriched preparations from untreated L363 cells reduced NK-92-cell cytotoxicity against K562 targets and decreased NKG2D expression, despite increasing IFN-γ production. In contrast, preparations obtained after donor-cell treatment with eicosapentaenoic acid (EPA) or docosahexaenoic acid (DHA) produced less suppression of NK-cell killing and restored NKG2D expression and CD107a-associated degranulation. These results suggest that exposing MM cells to EPA or DHA changes the functional properties of the material they release rather than acting directly on the recipient NK cells. However, lipid, protein or RNA cargo responsible for the altered NK-cell response were not identified. Proposed mechanisms involving membrane lipid rafts, ceramide metabolism or EV biogenesis were not experimentally tested. The preparations were isolated using polymer precipitation, which may co-isolate soluble proteins and other extracellular particles, and all functional experiments relied on L363, NK-92 and K562 cell lines. No primary patient-derived MM cells, primary NK cells, animal models or clinical supplementation experiments were included. Thus, the evidence does not demonstrate that dietary EPA or DHA can restore NK-cell immunity in patients through EV modulation (Moloudizargari et al., 2020). In a novel multimodal therapeutic system, SFOVV@IR1061 that researchers developed to target DLBCL, they conjugated Sophora flavescens (SF)-derived extracellular vesicles (SFNPs), an oncolytic vaccinia virus (OVV), and an NIR-II fluorescent dye (IR1061). OVV exploits tumor endocytosis to selectively infect tumor cells, initiating oncolysis and ICD by releasing CRT, HSP70, HMGB1, and ATP, thereby maturing DCs and activating both innate and adaptive immunity. SFNPs, on the other hand, act as a natural, biocompatible nanocarrier for OVV, stabilizing the virus, improving its tumor-targeting capacity, and reducing its systemic toxicity. Moreover, the IR1061 dye enabled real-time tumor PAI and PTT under NIR-II laser irradiation. As such, it not only visualizes tumors in real time but also generates localized hyperthermia, which can augment the replication of OVV and the activation of innate and adaptive immunity. The combined oncolytic and immunogenic action of OVV, with the enhanced antitumor effect of IR1061 under PTT, synergistically promotes tumor apoptosis, increases M1 macrophage polarization, and reprograms the tumor immune microenvironment (Ji et al., 2025). Therefore, natural products may influence EV-mediated communication through different mechanisms. GZFL and EPA/DHA altered the abundance or functional effects of MM-cell EV-associated material, whereas Sophora flavescens-derived particles served as engineered therapeutic carriers. Evidence is strongest for the GZFL–ERK1–osteoclast pathway because it included compound-binding experiments and *in vivo* evaluation, although its clinical component was uncontrolled. The EPA/DHA findings remain cell-line-based, and the active EV cargo was not identified. The Sophora platform showed multimodal antitumor activity in mice but should not be interpreted as direct regulation of tumor-cell EVs. Overall, none of these approaches has yet demonstrated clinically validated restoration of antitumor immunity through EV modulation.

### 13.7 Practical barriers to clinical translation

Despite their therapeutic potential, the approaches reviewed do not yet define a standardized or interchangeable EV product. Proposed platforms include tumor-cell-, red-blood-cell-, dendritic-cell-, NK-cell-, CAR-T-cell- and mesenchymal-stromal-cell-derived EVs, as well as plant-derived EV-like nanoparticles. Their biological properties are affected by donor-cell origin, culture and activation conditions, cargo-loading procedures and surface engineering (Welsh et al., 2024; Rankin-Turner et al., 2021). This heterogeneity is particularly evident among NK-cell-derived preparations generated from cell lines, immortalized cells or differently activated primary cells. Direct comparison has demonstrated source- and stimulation-dependent differences in EV yield, molecular cargo and cytotoxic activity (Samara et al., 2023; Matchett and Kornbluth, 2023; Cecchetti et al., 2024; Khatib et al., 2024; Silva et al., 2021) (Samara et al., 2023; Matchett and Kornbluth, 2023; Cecchetti et al., 2024; Khatib et al., 2024; Kassi et al., 2024). Patient-derived preparations introduce additional biological and pre-analytical variability related to disease context, treatment exposure and sample handling, while blood contains EVs originating from malignant, immune, platelet, endothelial and stromal cells (Welsh et al., 2024; Nieuwland and Siljander, 2024). Consequently, the efficacy and safety of one preparation cannot be extrapolated directly to another.

Isolation and quality control remain major technological barriers. Ultracentrifugation, density-based separation, polymer precipitation, filtration and differential centrifugation recover preparations with different yields, purity and EV-subpopulation composition. These methods may also co-isolate soluble proteins, lipoproteins and particle aggregates, while the endosomal origin of the complete functional preparation is rarely established (Welsh et al., 2024; Silva et al., 2021; Rohde et al., 2019; Nguyen et al., 2020; Brennan et al., 2020). Clinical development therefore requires scalable and reproducible manufacturing processes with predefined critical quality attributes. Release criteria should assess particle identity, concentration and size; purity; sterility; endotoxin and *mycoplasma* contamination; residual host-cell DNA and non-EV protein; cargo-loading efficiency; unencapsulated therapeutic cargo; stability; and mechanism-linked functional potency (Silva et al., 2021; Rohde et al., 2019; Nguyen et al., 2020; Brennan et al., 2020; Paolini et al., 2022). Tumor-cell-derived products require particular attention because their intended antigenic activity may be accompanied by oncogenic, tumor-promoting or immunosuppressive cargo (Huang et al., 2017b; Huang et al., 2022; Chen et al., 2018). Engineered products also require assessment of residual loading reagents, unintended cargo, free therapeutic molecules and off-target biological activity (Liu et al., 2025; Zhang et al., 2025; Liu Q. et al., 2022; Rankin-Turner et al., 2021; Silva et al., 2021).

Dose determination is similarly unresolved because experimental exposure has been expressed as particle number, protein mass, preparation volume or donor-cell equivalents, preventing straightforward comparisons between studies (Nguyen et al., 2020). Administration routes, treatment schedules and exposure durations also differ substantially, and some of the reviewed preclinical effects required repeated or high-dose administration (Liu et al., 2025; Liu Q. et al., 2022; Samara et al., 2023; Cecchetti et al., 2024). Particle number or total protein alone cannot demonstrate equivalent biological activity between batches or platforms. Translation therefore requires standardized dose-ranging studies, pharmacokinetic and biodistribution analyses, evaluation of tissue retention and clearance, repeated-dose toxicity testing and potency assays linked to the proposed mechanism of action (Silva et al., 2021; Nguyen et al., 2020; Wiklander et al., 2015).

Finally, clinical-scale production will require qualified cell banks, controlled culture media, closed manufacturing systems, reproducible upstream and downstream processing, batch-to-batch consistency and validated storage and transport conditions. Regulatory classification and evidence requirements will depend on the cellular source, degree of engineering, incorporated cargo and intended clinical use. Within the therapeutic evidence set summarized in this review, no proposed EV product was administered to patients with a hematological malignancy. Early-phase clinical trials should therefore proceed only after product identity, manufacturing reproducibility, mechanism-linked potency and product-specific safety have been established (Silva et al., 2021; Rohde et al., 2019; Nguyen et al., 2020; Brennan et al., 2020; Paolini et al., 2022; Wiklander et al., 2015; Lener et al., 2015).

## 14 EV isolation technologies and methodological heterogeneity

Across the reviewed studies, differential ultracentrifugation was the predominant EV-enrichment method, commonly using a final centrifugal force of approximately 100,000–120,000 × g. However, protocols differed substantially in pre-clearing steps, filtration, washing, centrifugation time and repeat pelleting, even when described using the same methodological term. Some studies instead examined pellets collected at 10,000–25,000 × g, which are more likely to contain mixed populations of medium/large EVs, cellular fragments and protein aggregates. High-speed centrifugation enriches smaller EVs but does not completely separate EV subtypes or establish their endosomal origin (Welsh et al., 2024; Ling et al., 2025; Shao et al., 2024; Liu et al., 2020; Dubois et al., 2025; Du et al., 2023; Tripathi et al., 2023; Razi et al., 2023; Pucci et al., 2021; Reiners et al., 2013; Wang D. et al., 2024; Wang Q-M. et al., 2024; Smallwood et al., 2016; Du et al., 2025; Peng et al., 2021; Yao et al., 2014; Tohumeken et al., 2020). Polymer precipitation and commercial isolation kits were also frequently used, particularly for serum, plasma and small-volume culture samples. These methods are simple and provide relatively high particle recovery, but they may co-isolate soluble proteins, lipoproteins, nucleoprotein complexes and polymer-associated aggregates. This limitation is especially important for circulating preparations, which contain EVs from malignant, immune, platelet, endothelial and stromal cells rather than an exclusively tumor-derived population (Ling et al., 2022; Tian et al., 2021; Batista et al., 2021; Weng et al., 2023; Hagiwara et al., 2025; Benites et al., 2019; Li et al., 2025; Mizuhara et al., 2023; Wang et al., 2016; Wang et al., 2015). Density-gradient purification, size-exclusion chromatography, ultrafiltration and tangential-flow filtration were used less frequently. These approaches can improve purity or scalability, although they may reduce yield and still cannot completely resolve vesicles from similarly sized contaminants. Immunoaffinity capture increased enrichment of selected CD30^−^, CD34^−^or CAR-positive particles but recovered only marker-positive subsets and did not independently prove cellular origin or exosomal biogenesis (Garbin et al., 2023; Black et al., 2016; Hong et al., 2014; Yao et al., 2014; Tohumeken et al., 2020; Storci et al., 2024; Cho et al., 2024). EV characterization was similarly inconsistent. Some studies combined particle sizing, electron microscopy, multiple positive and negative protein markers, density analysis and functional controls, whereas others relied primarily on particle size or one or two tetraspanins. Neither particle size nor CD9, CD63 or CD81 expression alone demonstrates EV purity, tumor-cell origin or endosomal biogenesis. Isolation methodology therefore represents an important source of between-study heterogeneity and may partly contribute to divergent biological findings. Most functional isolates should consequently be described as EV-enriched, sEV-enriched or medium/large-EV-enriched preparations rather than as purified exosomes. Future investigations should standardize pre-analytical handling, report complete isolation protocols and use orthogonal characterization and purity controls appropriate to the sample type and intended application (Welsh et al., 2024).

## 15 Conclusion and future directions

EVs are important mediators of communication between malignant, immune and stromal cells in hematological malignancies. Across lymphoma, leukemia and multiple myeloma, EV-associated proteins, lipids and nucleic acids have been linked to macrophage polarization, MDSC expansion, dendritic-cell dysfunction, lymphocyte exhaustion, immune-checkpoint regulation and treatment resistance. Nevertheless, the evidence remains predominantly preclinical and is heterogeneous in experimental design, EV isolation, dosing and recipient-cell models. Many reported mechanisms rely on cell lines, pharmacological inhibitors or associations between EV cargo and immune phenotypes, while comparatively few have been confirmed through cargo-specific loss-of-function, rescue experiments, independent replication and clinically relevant animal models. EVs should therefore be considered context-dependent regulators of immunity rather than uniformly immunosuppressive disease drivers. The dominant EV-mediated interactions also differ among malignancies. In lymphoma, the available evidence emphasizes checkpoint-related suppression, macrophage reprogramming, antigen or antibody decoy activity and, in selected settings, preservation of immunogenic tumor antigens. In leukemia, EVs appear particularly relevant to bone-marrow niche remodeling, myeloid-cell reprogramming and metabolic or functional impairment of T and NK cells. In multiple myeloma, EVs connect immune suppression with stromal signaling, angiogenesis, osteoclast activation and therapy-induced changes in the bone-marrow microenvironment. These differences argue against treating EV-mediated immune regulation as a single mechanism shared uniformly across hematological malignancies.

Across the immune-cell populations examined, only a limited number of recurrent signaling nodes emerge. STAT3/JAK–STAT signaling is implicated in macrophage polarization, MDSC expansion, monocyte activation and checkpoint-related responses, whereas NF-κB and PI3K/Akt/mTOR contribute mainly to inflammatory reprogramming, myeloid-cell survival and immunosuppressive differentiation. TGF-β and PD-1/PD-L1 signaling recur in the suppression of NK- and T-cell activity and in the induction of tolerogenic or regulatory phenotypes. However, these shared nodes should not be interpreted as a single conserved EV-mediated network. Their upstream EV cargos, recipient-cell contexts and downstream consequences differ across leukemia, lymphoma and multiple myeloma. Furthermore, the supporting evidence ranges from pathway associations to pharmacological inhibition and genetic perturbation, and most mechanisms have not been independently reproduced under standardized EV-isolation, dosing and functional-assay conditions. The apparent convergence may therefore represent the reuse of common downstream immune-signaling machinery rather than a universal EV-specific mechanism.

Several biological questions must be resolved before these mechanisms can be translated. First, the cellular origin of functionally relevant EV populations remains uncertain because tumor-, immune-, platelet-, endothelial- and stromal-cell-derived vesicles coexist in blood and bone marrow. Second, it is unclear whether individual cargos are necessary and sufficient for immune reprogramming or whether combinations of surface ligands, proteins, metabolites and RNAs act cooperatively. Third, the contribution of EV-associated material must be separated from that of soluble cytokines, lipoproteins and direct cell–cell interactions. Fourth, EV activity may vary with disease subtype, clonal evolution, anatomical compartment, treatment exposure and disease stage. Longitudinal studies are therefore required to determine whether EV-mediated immune changes precede progression or resistance or merely reflect established disease. Finally, the biodistribution, uptake specificity, intracellular fate and clearance of endogenous and therapeutically administered EVs remain inadequately defined, particularly within the bone-marrow niche.

Technological limitations currently prevent reliable comparison across studies. Differences in blood-collection tubes, processing intervals, platelet depletion, storage, freeze–thaw exposure and starting sample volume can alter EV yield and cargo measurements. Ultracentrifugation, polymer precipitation, size-based separation and immunocapture also recover different vesicle populations and amounts of non-vesicular material. Future studies should use MISEV-aligned reporting, include appropriate purity and negative-marker controls and avoid classifying preparations as exosomes unless endosomal biogenesis is demonstrated. Standardized normalization to particle number, sample volume, donor-cell number or defined reference materials is also needed. Single-vesicle phenotyping, high-sensitivity flow cytometry, immunoaffinity capture and multi-omics analysis may improve resolution, but these methods require interlaboratory benchmarking, reproducibility testing and consensus thresholds. Physiologically relevant EV doses and orthogonal labeling or tracking approaches are equally important because lipophilic dyes, protein-normalized dosing and high experimental concentrations can produce misleading uptake or functional results.

Biomarker development should follow a staged validation framework. Most EV-associated miRNAs, proteins and surface markers discussed in this review remain exploratory candidates identified in small, retrospective or cross-sectional cohorts. Discovery studies should be followed by analytical validation of assay precision, stability, limits of detection and normalization, and then by testing in independent cohorts with clinically relevant comparator groups. Diagnostic or prognostic studies should prespecify thresholds and report sensitivity, specificity, calibration and incremental value over established clinical and molecular variables. Multicenter prospective cohorts should collect serial samples before, during and after treatment to determine whether EV signatures predict response, relapse, toxicity or immune-cell recovery. A candidate should not be considered clinically validated until its performance is reproduced using a standardized assay and shown to influence a defined clinical decision. At present, none of the reviewed EV-associated candidates fulfils all of these requirements for routine clinical implementation.

Translational research should prioritize mechanisms supported by convergent causal evidence rather than associations alone. Candidate cargos should be tested through selective depletion from donor-cell EVs, receptor-specific inhibition in recipient cells and rescue experiments. These studies should progress from standardized cell systems to primary patient-derived cells, multicellular bone-marrow models, organoids and immunocompetent or humanized animal models. Early-phase clinical trials should incorporate pharmacokinetic, biodistribution, pharmacodynamic and immune-monitoring endpoints rather than evaluating tumor response alone. Trials of engineered or immune-cell-derived EVs will also require predefined identity, purity and potency assays; reproducible manufacturing; stability during storage and transport; and evidence that the administered dose can be produced consistently under good manufacturing practice conditions.

Combination immunotherapy represents a particularly relevant opportunity. Selective neutralization of EV-associated PD-L1, TGF-β or other suppressive cargos could potentially improve checkpoint blockade, while inhibition of CD20-bearing EV decoys may restore the activity of anti-CD20 antibodies in lymphoma. Preventing EV-driven MDSC expansion, macrophage polarization or NK- and T-cell exhaustion may enhance CAR-T-, CAR-NK- or bispecific-antibody therapies. Conversely, dendritic-cell-, NK-cell- and CAR-T-cell-derived EVs could be developed as cell-free vaccines or cytotoxic platforms and combined with agents that induce immunogenic tumor-cell death. In multiple myeloma, EV-directed interventions may be most rational when combined with BCMA-directed or NK-cell-based therapies rather than assuming that checkpoint inhibition will be effective across all settings. These combinations should be tested with appropriate single-treatment controls, rational treatment sequencing and biomarkers demonstrating that the EV-directed component modifies its intended pathway.

Clinical implementation will nevertheless present substantial challenges. Global inhibition of EV biogenesis or uptake could disrupt physiological communication and remove beneficial immune-cell-derived EVs; consequently, tumor-selective targeting is preferable to nonspecific EV suppression. Engineered products must also be evaluated for off-target tissue accumulation, immunogenicity, complement or coagulation activation, transfer of oncogenic material and batch-to-batch variability. Regulatory classification, scalable purification, sterile production, storage stability, cost and release criteria remain unresolved for many EV-based products. These considerations are especially important for off-the-shelf therapies, for which consistency and safety must be demonstrated without losing the functional advantages of the donor cells.

The immediate priorities for the field are therefore to define the causal EV populations and cargos operating in patients, standardize pre-analytical and analytical methods, establish reproducible disease-specific biomarkers and conduct mechanism-informed clinical trials. Progress will depend less on cataloguing additional EV cargos than on determining which EV populations are biologically necessary, measurable with robust assays and therapeutically actionable. Addressing these priorities will clarify whether EVs can move from promising experimental mediators to reliable biomarkers and clinically useful components of combination immunotherapy.

## Glossary

ALCL: Anaplastic large cell lymphoma
ALL: Acute lymphoblastic leukemia
AML: Acute myeloid leukemia
APC: Antigen-presenting cell
ApoV: Apoptotic vesicle
ATP: Adenosine triphosphate
BAG6: BCL2-associated athanogene 6
BCR: B-cell receptor
BCR-ABL: Breakpoint cluster region–Abelson tyrosine kinase fusion protein
BM: Bone marrow
BM-MSC: Bone marrow mesenchymal stem/stromal cell
BTLA: B- and T-lymphocyte attenuator
CAR: Chimeric antigen receptor
**CAR** ^**+**^ **EV**: Chimeric antigen receptor-positive extracellular vesicle
CAR-EV: Chimeric antigen receptor-engineered extracellular vesicle
CAR-T: Chimeric antigen receptor T cell
CAR-T-EV: Chimeric antigen receptor T-cell-derived extracellular vesicle
CCL: C-C motif chemokine ligand
CD: Cluster of differentiation
cHL: Classical Hodgkin lymphoma
CLL: Chronic lymphocytic leukemia
CLL-1: C-type lectin-like molecule 1
CML: Chronic myeloid leukemia
CMML: Chronic myelomonocytic leukemia
CRT: Calreticulin
CTL: Cytotoxic T lymphocyte
CTLA-4: Cytotoxic T-lymphocyte-associated protein 4
DC: Dendritic cell
DC-EV: Dendritic cell-derived extracellular vesicle
DC-Exo: Dendritic cell-derived exosome
DCleu: Leukemia-derived dendritic cell
DC-TEX: Tumor-exosome-loaded dendritic cell
DHA: Docosahexaenoic acid
DLBCL: Diffuse large B-cell lymphoma
DNA: Deoxyribonucleic acid
Dox: Doxorubicin
EBV: Epstein–Barr virus
ELISA: Enzyme-linked immunosorbent assay
EPA: Eicosapentaenoic acid
ER: Endoplasmic reticulum
EV: Extracellular vesicle
FasL/FASL: Fas ligand
FL: Follicular lymphoma
FOXP3: Forkhead box P3
GPI: Glycosylphosphatidylinositol
GZFL: Guizhi Fuling decoction
HLA: Human leukocyte antigen
HLA-DR: Human leukocyte antigen-DR isotype
HMGB1: High-mobility group box 1
HPSE: Heparanase
HSC: Hematopoietic stem cell
HSP70: Heat shock protein 70
ICANS: Immune effector cell-associated neurotoxicity syndrome
ICD: Immunogenic cell death
IDO1: Indoleamine 2,3-dioxygenase 1
IFN-γ: Interferon gamma
IL: Interleukin
JAK: Janus kinase
LAK: Lymphokine-activated killer
LAK-Exo: Exosomes derived from lymphokine-activated killer cells
LAG3: Lymphocyte activation gene 3
LEX: Leukemia cell-derived exosome
LEX-CD8086: Leukemia cell-derived exosomes engineered to overexpress CD80 and CD86
lncRNA: Long non-coding RNA
M0: Unpolarized macrophage
M1: Classically activated macrophage
M2: Alternatively activated macrophage
MAPK: Mitogen-activated protein kinase
MCL: Mantle cell lymphoma
MDSC: Myeloid-derived suppressor cell
MHC: Major histocompatibility complex
MHC-II: Major histocompatibility complex class II
MICA: Major histocompatibility complex class I polypeptide-related sequence A
MIIC: MHC class II-loading compartment
MISEV2023: Minimal Information for Studies of Extracellular Vesicles 2023
miRNA: MicroRNA
MLC: Mixed lymphocyte culture
MM: Multiple myeloma
M-MDSC: Monocytic myeloid-derived suppressor cell
MMP-9: Matrix metalloproteinase 9
mRNA: Messenger RNA
MSC: Mesenchymal stem/stromal cell
mTOR: Mechanistic target of rapamycin
MUC1: Mucin 1
**NAD** ^**+**^: Nicotinamide adenine dinucleotide
NAMPT: Nicotinamide phosphoribosyltransferase
NF-κB: Nuclear factor kappa B
NIR-II: Second near-infrared window
NK: Natural killer
NK-EV: Natural killer cell-derived extracellular vesicle
NK-Exo: Natural killer cell-derived exosome
NKG2D: Natural killer group 2 member D
NKG2DL: Natural killer group 2 member D ligand
NLC: Nurse-like cell
NTA: Nanoparticle tracking analysis
NVEP: Non-vesicular extracellular particle
OVV: Oncolytic vaccinia virus
PAI: Photoacoustic imaging
PBMC: Peripheral blood mononuclear cell
PD: Bortezomib plus dexamethasone regimen
PD-1: Programmed cell death protein 1
PD-L1: Programmed death-ligand 1
**PGE**_**2**_: Prostaglandin E_2_
PI3K: Phosphoinositide 3-kinase
PMBCL: Primary mediastinal B-cell lymphoma
pMHC: Peptide–major histocompatibility complex
PRIME Exos: Programmed immune-engaging exosomes
PTT: Photothermal therapy
qPCR: Quantitative polymerase chain reaction
RANKL: Receptor activator of nuclear factor-κB ligand
REV: Red blood cell-derived extracellular vesicle
RNA: Ribonucleic acid
ROS: Reactive oxygen species
S1P: Sphingosine-1-phosphate
sEV: Small extracellular vesicle
SF: *Sophora flavescens*
SF-EV: *Sophora flavescens*-derived extracellular vesicle
SFNP: *Sophora flavescens*-derived nanoparticle
shRNA: Short hairpin RNA
siRNA: Small interfering RNA
SPHK1: Sphingosine kinase 1
STAT: Signal transducer and activator of transcription
TAM: Tumor-associated macrophage
TDE: Tumor-derived extracellular vesicle
TEM: Transmission electron microscopy
TEV: Tumor-derived extracellular vesicle
TEX: Tumor-derived exosome
TGF-β: Transforming growth factor beta
Th: T helper
Th1: Type 1 T helper cell
Th2: Type 2 T helper cell
Th17: Type 17 T helper cell
TIGIT: T-cell immunoreceptor with immunoglobulin and ITIM domains
TIM-3: T-cell immunoglobulin and mucin-domain containing-3
TLR: Toll-like receptor
TME: Tumor microenvironment
TNF-α: Tumor necrosis factor alpha
TRAIL: TNF-related apoptosis-inducing ligand
Treg: Regulatory T cell
TSG101: Tumor susceptibility gene 101 protein
ULBP: UL16-binding protein
